# Impact of the Magnitude and Duration of Weight Loss Procedures in Combat Sports Athletes: A Systematic Review

**DOI:** 10.3390/nu18213474

**Published:** 2026-10-22

**Authors:** Román Illescas-Quiroga, Alireza Naderi, Juan José Ramos-Álvarez, Alberto Pérez-López, Raúl Domínguez

**Affiliations:** 1 Facultad de Medicina y Ciencias de la Salud, Departamento de Ciencias Biomédicas, Área de Educación Física y Deportiva, University of Alcala, 28805 Alcalá de Henares, Spain; rillescas@gmail.com (R.I.-Q.); alberto.perezl@uah.es (A.P.-L.); 2 Department of Sport Physiology, Boroujerd Branch, Islamic Azad University, Boroujerd 6915136335, Iran; naderi.alireza1366@gmail.com; 3 School of Sport Medicine, Faculty of Medicine, Complutense University of Madrid, 28040 Madrid, Spain; jjramosa@ucm.es; 4 Grupo de Optimización del Entrenamiento Deportivo (GOPED), Department of Human Motor and Sports Performance, Sevilla University, 41013 Sevilla, Spain

**Keywords:** body composition, decision-making, hydration, nutrition, physical performance

## Abstract

Background: Weight manipulation is standard practice in combat sports to compete in lighter divisions, but the thresholds of duration and magnitude impairing performance remain unclear. Objectives: To evaluate the effects of progressive (PWL) versus rapid weight loss (RWL) across three magnitudes of body mass (BM) reduction, moderate (<5%), aggressive (≥5% to <10%), and extreme (≥10% BM), on performance in combat sport athletes. Methods: PubMed, Medline, Scopus, Scielo, and SportDiscus were searched for interventions published from January 2000 to 30 September 2025. Two independent researchers performed screening, data extraction, and quality assessment (PEDro scale). Results: Twenty reports corresponding to 18 independent studies met the inclusion criteria (mean PEDro score 5.30; fair quality), comprising 363 unique athletes (322 men, 41 women). Extreme weight loss (≥10% BM) via PWL was associated with impaired technical skill, decision-making, and mood profile, although this evidence was derived from only three independent samples. Aggressive RWL (≥5% to <10% BM) through acute dehydration reduced maximal strength, power, and sport-specific fitness in several but not all studies, with deficits still present 24 h after the weigh-in. Moderate reductions yielded heterogeneous findings, with impairments of mood, balance, and resting maximal voluntary contraction in some reports and no significant effects in others. Conclusions: Effects appeared graded by the magnitude of BM reduction, but the evidence is unevenly distributed, and no study examined extreme RWL. Findings should be interpreted with caution.

## 1. Introduction

Combat sports are individual modalities with great relevance worldwide, with amateur boxing, taekwondo, judo, wrestling, and karate being part of the Olympic Games, while professional boxing and mixed martial arts are practiced and followed by millions of people [1].

Every combat sport modality has its regulations, which describe the sport-specific characteristics, define actions that athletes are allowed to perform to win, and detail competition duration (i.e., number of bouts, bout duration, time of recovery between bouts). A fundamental feature of combat sports is the classification of athletes into body mass (BM)-based weight divisions, allowing competitors to face opponents with broadly similar physical (e.g., strength, speed, and power) and anthropometric (e.g., limb length) characteristics [2]. Accordingly, athletes must undergo an official pre-competition weigh-in, commonly referred to as *making weight*, to verify eligibility for their designated weight category. To gain a competitive advantage by facing lighter opponents, many combat sports athletes intentionally compete in a weight category below their habitual training BM [3].

Following this rationale, combat athletes often implement *weight loss* (WL) strategies hours, days, or even weeks before the official weigh-in [4,5]. However, acute reductions in BM may impair exercise performance depending on several factors, including the magnitude of WL, the recovery time between weigh-in and competition, the specific WL strategy employed, and the athlete’s physiological adaptation to these practices [6,7]. Based on the timing of BM reduction, WL strategies are generally classified as *rapid WL* (RWL) when initiated within the final seven days before competition, and *progressive WL* (PWL) when implemented over longer periods [5,8,9]. Regarding magnitude, most athletes reduce less than 5% of their BM, whereas a substantial proportion lose between 5% and less than 10%, and athletes competing in sports such as mixed martial arts, Muay Thai, and Sambo may occasionally exceed 10% BM reduction [10,11]. These observations emphasize the need to better understand how both the duration and magnitude of WL influence competitive performance.

The physiological consequences of WL largely depend on the methods used to achieve the desired BM. Dietary energy restriction may impair cardiovascular function, increase perceived fatigue, disturb electrolyte balance, and reduce satiety [12]. In contrast, short-term reductions in dietary fiber intake (2–7 days) decrease intestinal content and facilitate BM reduction without compromising exercise performance [3,13]. Manipulation of body water content through fluid restriction or dehydration strategies has more pronounced physiological consequences. Reductions in body water exceeding approximately 3% may impair thermoregulation, oxidative capacity, and subsequent rehydration while increasing thirst and physiological strain [6,13,14]. Likewise, combining dehydration with carbohydrate restriction may reduce glycogen availability and compromise high-intensity glycolytic performance unless adequate post-weigh-in nutritional recovery is achieved [1,15]. Dehydration induced through active sweating (e.g., exercising in the heat or wearing impermeable clothing) primarily increases fatigue and thermal discomfort, whereas passive methods (e.g., sauna or hot-water immersion) have been associated with greater plasma osmolality, muscle damage, and inflammatory responses [14].

Despite these well-described physiological effects, the influence of WL on competitive performance remains controversial. Some studies have reported that greater pre-competition WL and subsequent BM regain are associated with improved competitive success [16], whereas others have found no relationship between weight manipulation and competition outcomes [17,18]. Similarly, experimental studies have yielded conflicting findings, with some reporting impaired repeated high-intensity performance following WL [4,18], while others observed no detrimental effects [19,20]. These inconsistencies may be explained, at least in part, by the considerable heterogeneity in the duration and magnitude of WL protocols evaluated across studies. To facilitate a more meaningful interpretation of the available evidence, WL interventions should be considered according to both their duration (RWL vs. PWL) and magnitude.

Consistent with previous clinical observations, the present review classifies WL magnitude into three functional tiers: moderate (<5% BM), aggressive (≥5% to <10% BM), and extreme (≥10% BM). This magnitude-based stratification was defined a priori for data-extraction purposes, informed by descriptive reports indicating that most combat sport athletes reduce less than 5% of BM, whereas markedly larger reductions (≥10% BM) are reported almost exclusively in disciplines such as mixed martial arts, Muay Thai, and Sambo [10,11]. Whether these operational categories also correspond to meaningful differences in recovery feasibility and performance outcomes is explored descriptively in Section 4.5. By adopting this framework, we move beyond the temporal binary of “rapid vs. gradual” loss to focus on the biological severity of the intervention and its specific impact on competitive output.

Therefore, the aims of this systematic review are to: (i) compare the effects of RWL and PWL on sport-specific performance in combat sport athletes; (ii) examine the influence of WL magnitude (moderate, aggressive, and extreme) on sport-specific performance; (iii) explore patterns in performance outcomes according to the combination of WL duration and magnitude; and (iv) map how the available evidence is distributed across the resulting magnitude × duration categories in order to identify the conditions for which empirical evidence is currently lacking. A clearer understanding of these factors may inform evidence-based recommendations for safer and more effective weight-management practices in combat sports.

## 2. Materials and Methods

### 2.1. Design of the Systematic Review

The present systematic review was performed according to the PRISMA 2020 statement [21] and following PICOS (population, intervention, comparison, outcome, and setting) criteria [22] (see Table 1). This protocol was registered in the International Prospective Register of Systematic Reviews (PROSPERO) database (CRD420261467473) on 5 August, 2026. Registration was therefore retrospective, as it took place after the literature search and the study selection had been completed (see PRISMA 2020 checklist in the Supplementary Table S1). Generative artificial intelligence tools were used during manuscript preparation for language editing and for verifying the internal consistency of the reported data against the original publications. Study selection, data extraction, methodological quality assessment, and the interpretation of the findings were performed exclusively by the authors, who take full responsibility for the content of the manuscript.

### 2.2. Eligibility Criteria

Based on the background described in the introduction, the research question of this systematic review was as follows: “Do the duration (PWL vs. RWL) and the magnitude (moderate vs. aggressive vs. extreme) of the WL procedure influence sport-specific performance in trained combat sport athletes?” To answer this question, and according to the PICOS criteria summarized in Table 1, the following inclusion criteria were defined: (i) criteria related to the participants: to include a sample formed only by participants defined as trained or competitive combat sport athletes; (ii) criteria related to the intervention: to include an a priori WL procedure, which specifies the duration and the magnitude of the WL protocols. RWL was operationally defined as WL implemented within ≤7 days before competition, and PWL as WL implemented over a longer period (>7 days). This classification was applied according to the duration of the protocol actually reported in each study, irrespective of the terminology used by the original authors, so that some studies which describe their intervention as RWL were classified as PWL because the reduction was implemented over more than 7 days. Likewise, WL magnitude was categorized according to the reduction targeted by each protocol; when the reduction actually achieved was reported, it is stated in Section 3 and in, so that a protocol designed to reduce 5% BM that achieved 4.8 ± 1.1% BM was retained within the aggressive category; (iii) criteria related to the control: to include a comparison between different experimental conditions (crossover studies) or over the time (parallel group studies); (iv) criteria related to the outcome: to include some outcome related to physical and/or cognitive performance in combat sport modalities; (v) criteria related to the studies: to be qualified as an intervention study, which includes a comparison with a control group and/or experimental condition.

### 2.3. Information Sources and Literature Search Strategy

The literature was searched using PubMed, Scielo, Medline, Scopus, and SportDiscus databases. The search was restricted to studies published between 1 January 2000, and 30 September 2025, and restricted to documents published in English, Spanish, or Portuguese. The search was conducted cumulatively through successive updates, and the last search in all five databases was run on 30 September 2025, which is therefore the date up to which the evidence base is complete. The year 2000 was established as the lower limit because the weight categories, the competition rules, and the weigh-in procedures of the main combat sport modalities have been modified over the previous decades, so that studies published before that date would not reflect the current competitive context in which WL procedures are implemented. The restriction to documents published in English, Spanish, or Portuguese was based on the languages managed by the researchers who performed the screening and the data extraction, which allowed them to assess the full text of each record without depending on translations. Grey literature sources, including preprints and unpublished studies, were not included; only peer-reviewed published articles were considered. Records retrieved from the five databases were exported to a spreadsheet, and duplicates were identified in a Microsoft Excel spreadsheet by sorting and comparing author names, article title, journal, and year of publication, and they were removed by two researchers (R.I.-Q. and R.D.) before title and abstract screening; no automated deduplication software was used.

The search strategy used in all the databases was as follows: (“Combat sport” OR judo OR taekwondo OR “Mixed martial arts” OR wrestlers OR boxing OR “martial arts” OR karate) (concept 1) AND (nutrition OR “weight cutting” OR “making weight” OR “rapid weight loss” OR “weight reduction” OR “body composition” OR “body mass reduction” OR dehydration OR rehydration OR “weight making strategies” OR “weight loss methods” OR “body fat” OR “body fluid” OR “energy restriction” OR “dietary intake” OR recovery) (concept 2) AND (competition OR “Olympic games” OR elite OR cognition OR abilities OR physiology OR injuries OR performance OR “emotional intelligence”) (concept 3) (information extended in Supplementary Table S2). Two researchers (R.I.-Q and R.D.) independently checked all the search results and performed the screening and eligibility assessment of studies. After contrasting outcomes between these two researchers, results showing discrepancies were evaluated by a third researcher (A.L.-R.). All documents that did not meet the established PICOS criteria were discarded.

### 2.4. Data Extraction

Two authors (R.I.-Q and R.D.) independently extracted data from each study using a predefined data sheet and discussed and resolved by consensus any potential discrepancy afterward.

The following information was compiled from each study: information related to the sample (sample size, sex, sport modality practiced by the participants and maximal competitive level), experimental conditions (randomized crossover designs) or groups (designs based on parallel groups), WL protocol (magnitude of the WL, expressed as % BM, strategies used for WL and duration of the intervention), outcomes (sport performance variables analyzed) and results (for all variables analyzed irrespective of the direction or statistical significance of the findings). When a significant time effect was reported, variables were presented according to the increase observed at post-intervention (expressed as %), compared to pre-intervention, which was calculated using the following equation: (mean value at post-intervention—mean value at pre-intervention)/mean value at pre-intervention × 100. Otherwise, when a significant effect for the group or condition was reported, results were expressed as the % of the difference between the experimental conditions (EC) or between experimental (EG) and control (CG) groups, calculated using the following equation: (EG − CG1)/CG1 × 100. All significant time × group interactions were also specified in the trials, which used an analysis of variance for repeated measures (ANOVA-RM). In addition, the *p*-value was included in all the results presented.

### 2.5. Quality Assessment of Studies

PEDro scale was used to assess the methodological quality of the studies. This scale comprises 11 items and was previously validated [23]. The PEDro scale was developed primarily for randomized controlled trials, so its application to the non-randomized designs included in this review should be regarded as an approximation of methodological quality rather than as a formal risk-of-bias assessment. Blinding of participants and of those administering the intervention (items 5 and 6) is rarely feasible in WL interventions, whereas blinding of outcome assessors (item 7) is feasible in some designs; for instance, when outcomes are scored from video recordings by independent evaluators. Tools developed specifically for exercise-training studies, such as TESTEX, have been proposed for this type of design [24]; the PEDro scale was nevertheless retained for comparability with previous reviews in combat sports. Questions were answered with a “yes” if the criteria were satisfied and with a “no” otherwise, so the maximal score possible for one study was 10 since the first item of the scale is not included in the final score. Based on final scores, studies were classified as showing excellent (10 points), good (7–9 points), fair (5–6 points), or poor (<5 points) methodological quality [25,26]. Each study’s quality assessment was independently performed by two authors (R.I. and R.D.). After that, these two researchers discussed and resolved by consensus any potential discrepancy, taking into consideration the opinion of a third author (A.L.-R.) if no consensus was reached.

### 2.6. Data Synthesis

A meta-analysis was considered inappropriate because of the heterogeneity of the WL protocols (magnitude, duration, and methods used to reduce BM) of the outcomes assessed and of the study designs, and because several reports were derived from the same cohort and therefore could not be treated as independent samples. The findings were consequently synthesized narratively and classified according to the magnitude of BM reduction (moderate, aggressive, or extreme) and the duration of the procedure (PWL vs. RWL). To avoid relying exclusively on statistical significance when interpreting the available evidence, the findings of each study were classified as unfavorable when a significant impairment of any outcome was reported, as favorable when a significant improvement was reported, and as null when no significant differences were detected in any of the outcomes assessed, and the three categories were considered in the synthesis.

## 3. Results

### 3.1. Literature Search and Study Selection

**Figure 1:**
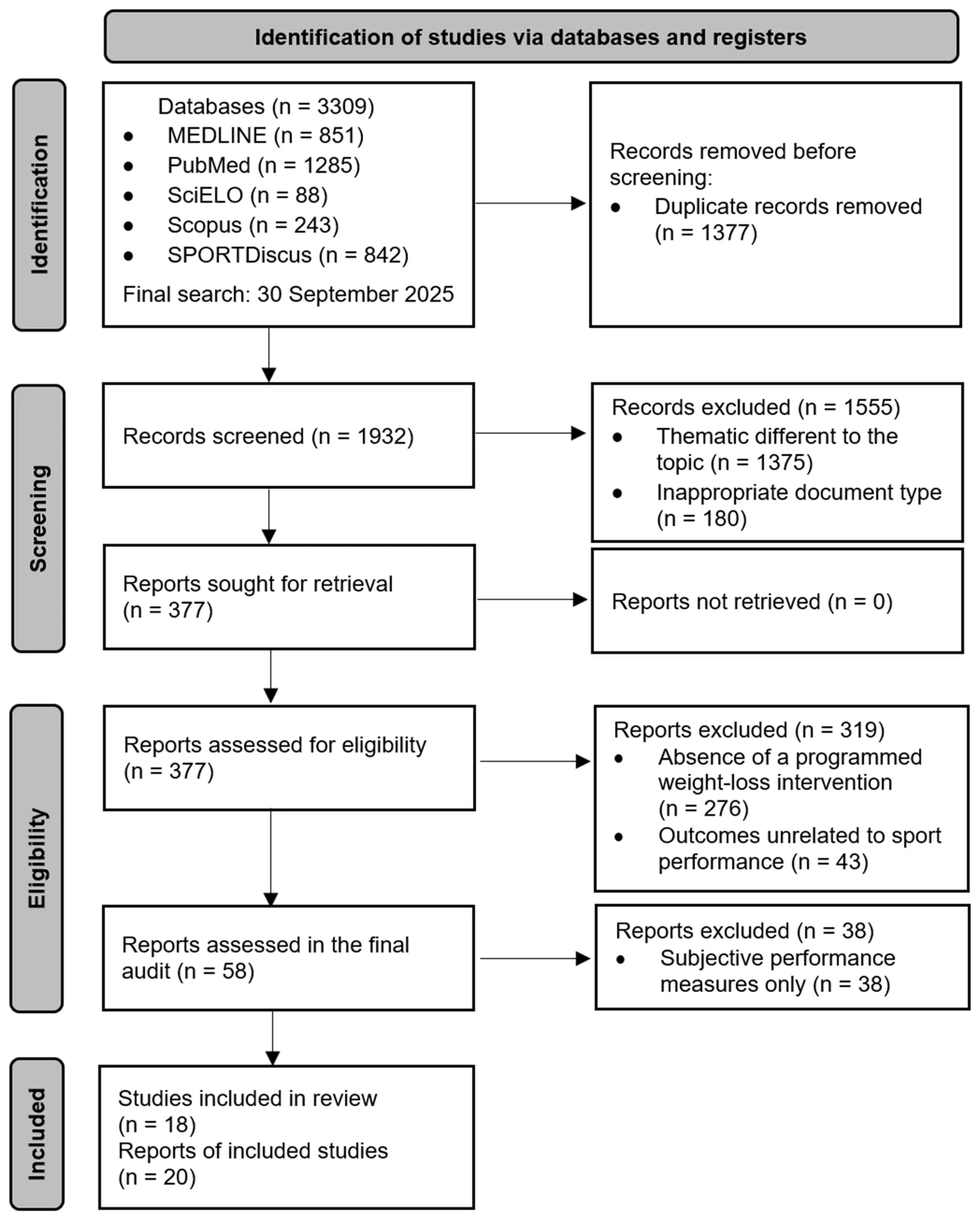
PRISMA 2020 flow diagram of the study selection process. The identification phase included a systematic search across five databases and updated metadata through September 2025. The final qualitative synthesis comprised 18 independent studies (20 reports) following a multi-stage screening for thematic relevance and objective performance metrics.

The selection process is detailed in the PRISMA flow diagram (Figure 1). The initial database search and metadata identification yielded 3309 records. Following the removal of duplicates (*n* = 1377), 1932 unique studies remained for title and abstract screening. During this first stage, 1555 documents were discarded: 1375 were thematically different from the topic, and 180 were excluded due to inappropriate document type. This left a pool of 377 manuscripts for further assessment. From this group, 319 articles were excluded (*n* = 276 for the absence of a programmed WL intervention and *n* = 43 for reporting outcomes unrelated to sport performance). A final refined audit of the remaining 58 reports led to the removal of 38 additional reports in which sport performance had been assessed only by subjective measures. Consequently, 20 reports corresponding to 18 independent studies met all PICOS criteria and were finally included in this systematic review.

### 3.2. Characteristics of the Studies Included in the Systematic Review

A total of 20 reports corresponding to 18 independent studies were selected for this systematic review to assess the effects of different WL strategies on combat sports performance, published up to 30 September, 2025. The final sample comprised 363 unique athletes, of whom 322 (88.7%) were men and 41 (11.3%) were women. Three reports [27,28,29] correspond to a single cohort of 39 male judokas (experimental group *n* = 20; control group *n* = 19) and were therefore counted only once for the calculation of participant totals, although each report was treated as a separate source of outcomes in the qualitative synthesis. According to the combat sports discipline, participants were grouped into 218 judokas, 49 wrestlers, 34 taekwondo athletes, 44 mixed martial arts fighters, 15 boxers, 2 Jiu-Jitsu, and 1 karateka. Regarding the time for making weight, 9 reports analyzed PWL [27,28,29,30,31,32,33,34,35] and 11 reports analyzed RWL [4,36,37,38,39,40,41,42,43,44,45], one of which also included a PWL group [41]. Regarding the magnitude of WL, 5 reports [27,28,29,30,32] analyzed an extreme WL corresponding to ≥10% BM using a PWL of a duration of 2 weeks in 4 reports [27,28,29,30] and 29 days in another report [32]. Eight reports [4,36,37,38,40,42,44,45] analyzed an aggressive WL (≥5% to <10% BM), all of them through RWL, and seven reports [31,33,34,35,39,41,43] analyzed a moderate WL (<5% BM).

### 3.3. Effects of WL Strategies in Combat Sport Athletes

The effects of the different WL strategies included in the reviewed studies are summarized in Table 2 and described below (Supplementary Table S3 includes information on all eligible outcomes of the included reports irrespective of statistical significance).

### 3.4. Effects of Extreme WL Strategies in Combat Sport Athletes

Of the five reports applying extreme WL, all five reported at least one statistically significant unfavorable finding, either as an impairment within the WL group or as a significant difference with respect to the athletes who maintained their BM, and none reported an overall null result. A progressive reduction of 10% BM over 2 weeks using a non-standardized WL protocol reduced the total number of throws during the Special Judo Fitness Test (SJFT) in male judokas compared with athletes who maintained their habitual BM [28]. Similarly, another study using the same WL protocol reported a deterioration in technical performance during a simulated taekwondo match, as reflected by a lower sport-specific skill execution index than in the control group [30]. Likewise, decision-making performance improved in athletes who maintained their BM over the same period, but not in those who performed an extreme PWL [27]. When a standardized protocol combining energy restriction with additional WL methods was used to achieve the same magnitude of BM reduction (10% over 2 weeks), athletes reported greater stress symptoms and poorer psychophysiological recovery [29]. Finally, an even longer extreme PWL intervention (29 days), combining energy restriction with additional WL methods before competition, increased total mood disturbance [32].

### 3.5. Effects of Aggressive WL Strategies in Combat Sport Athletes

Among aggressive WL interventions (≥5% to <10% BM), all reports investigated RWL protocols. Within this category, three reports described statistically significant performance impairments [4,37,44], four reported no significant impairment in any of the outcomes assessed [36,38,40,45], and one reported an improvement in a single outcome [42]. Acute reductions of approximately 5% BM achieved within 3 h through exercise in hot environments or sauna exposure combined with fluid restriction impaired handgrip strength [4,37], sport-specific grip endurance assessed by the Judogi Grip Strength Test (JGST), SJFT performance [37], medicine ball throw distance, and sled-push performance [4]. Similarly, a 5% BM reduction achieved over 2 days reduced both the total number of throws and the SJFT index relative to the values measured after 15 h of recovery in male judokas, although the change in BM was not statistically significant [44], whereas another study reported a higher velocity at the 4 mmol·L^−1^ blood lactate threshold following aggressive RWL [42]. In contrast, other investigations using non-standardized WL protocols found no changes in handgrip strength after 3 days of WL [45] or intermittent upper-body anaerobic performance assessed by an all-out arm-crank test [40]. In the only study that incorporated a 4 h recovery period with ad libitum refeeding and rehydration, a reduction of 4.8 ± 1.1% BM did not impair simulated judo combat or repeated upper-body Wingate performance; power output improved from pre- to post-intervention in both the WL and the control groups (main effect of time, with no group or interaction effects) [36]. Finally, a standardized reduction of approximately 5.3% BM achieved over 28 to 30 h through energy restriction (~2.6% BM) combined with passive dehydration by hot-water immersion (~2.7% BM) did not affect countermovement jump height, handgrip strength, maximal isometric force, or performance during a 3 min all-out cycling test [38].

### 3.6. Effects of Moderate WL Strategies in Combat Sport Athletes

Evidence regarding moderate WL (<5% BM) is less consistent. In this category, one report showed no significant changes in any of the outcomes assessed [34], whereas four reported significant impairments: in mood in the men who reduced 3.4% BM [35], in the number of tokui-waza over 30 s [33], in reaction time and static balance [41], and in maximal voluntary contraction and time to exhaustion [43]; one report described mixed findings [39]; and one reported a more favorable mood profile in the WL group than in the control group [31].

The effects of moderate PWL were also heterogeneous. Yoshioka et al. [35] compared national-level judokas who reduced BM over a 3-week intervention (men, 3.4%; women, 4.9%) with judokas who did not, reporting significant changes in tension, fatigue, vigor, and total mood disturbance only in the men who reduced 3.4% BM, whereas no significant changes were observed in the women who reduced 4.9% BM. Similarly, in a personalized dietary intervention producing a 3.5% BM reduction, the WL group reduced fatigue and total mood disturbance and increased vigor, while the control group, which did not receive any dietary guidelines, increased tension, depression, anger, fatigue, and total mood disturbance [31]. In contrast, a PWL intervention of approximately 20 days resulting in a 3.7% BM reduction did not alter maximal isokinetic strength of the knee or shoulder musculature [34]. Likewise, reducing BM by 3.9% over approximately 4 weeks did not impair vertical jump performance or short-duration judo-specific performance, although performance during a longer judo-specific test was reduced [33]. Interestingly, Isacco et al. [39] reported mixed findings after a 3% BM reduction achieved through RWL: total work performed and maximal strength were relatively preserved, whereas the mood profile was less favorable and both the rating of perceived exertion and the perceived training load were higher than in athletes who maintained BM.

### 3.7. Quality Assessment of Studies

Quality scores for each study are shown in Table 3. The first finding is that none of the reviewed reports was considered of excellent methodological quality. Two reports were qualified as good (7 points) [27,30], 13 reports as fair, reaching 6 points [4,28,29,38,39,41,43] or 5 points [31,33,37,40,42,44], and 5 [32,34,35,36,45] as of poor methodological quality, with a score lower than 5 points. Therefore, the average score of this systematic review is 5.30 points on the PEDro scale, corresponding to a “fair” quality level.

## 4. Discussion

The systematic review aimed to analyze the impact of RWL versus PWL procedures, the magnitude of WL (moderate, aggressive, or extreme), and patterns according to the combination of WL duration and magnitude on specific sport performance in combat athletes. This approach helps identify conditions under which WL affects athletes’ cognitive and physical performance, including sport-specific technical execution.

### 4.1. Effects of Extreme WL Strategies in Combat Sport Athletes

Regarding extreme WL, the results of the studies included in the systematic review suggest an impairment of specific sport performance [27,28,30] and mood [29,32]. It should be noted that all the studies analyzing extreme reductions applied PWL protocols implemented over 2–4 weeks, so no inference can be drawn regarding the duration of the WL procedure. It should also be noted that these protocols combined energy restriction with dehydration-based methods (sauna exposure, training in plastic suits, and the use of laxatives), so the impairments observed cannot be attributed to energy restriction alone, and that one of these reports achieved a BM reduction of approximately 9.9% (from 72.56 to 65.41 kg) against a 10% target [29]. In these studies, the energy reduction was not standardized, except in one of them, in which it was fixed at 6.7 MJ/day [29], in combination with plastic clothes during training, laxatives, and saunas. De Sousa et al. [30] reported a significant improvement in skill execution during a simulated three-round combat in the control group, but not in national-level male taekwondo athletes who reduced 10% BM through a 2-week PWL protocol. The improvement was reported in athletes who did not experience any change in their BM. Similar results were reported in another study, which reported an increased decision-making performance in a game performance assessment instrument in a control group, but not in the group that underwent a 10% BM reduction over 2 weeks in national-level male judokas [27]. In the SJFT, which is considered the most common specific judo performance test used in the scientific literature [46], Fortes et al. [28] reported a negative effect of a 5% BM reduction weekly over 2 weeks. Discrepancies between the enhancement of athletes who maintain their BM and athletes who reduced 10% BM could be attributed to the absence of training adaptations in the group that trained using plastic suits and saunas [29]. In addition, when extreme PWL protocols include dehydration-based methods, hypohydration may add to the effects of the energy deficit, and other potential consequences described for WL, such as reduced bone mineral density [47], were not assessed in the included studies. Dehydration could also impair cardiorespiratory endurance through a reduction in plasma volume, a plausible mechanism that was not assessed in the included studies either. Nevertheless, skills reflect physical and cognitive performance. Therefore, in addition to a deteriorated physical condition after extreme PWL procedures, impaired cognitive performance could contribute to the negative results reported previously [27,28,29,30]. In fact, De Sousa et al. [27] showed an increased decision-making performance assessed in a game performance assessment instrument in a control group, but not in the extreme PWL group. From a psychological viewpoint, Fortes et al. [29] reported a negative effect on the Daily Analysis of Life Demands for Athletes and the Recovery-Stress Questionnaire for Athletes in national-level male judokas after a PWL 10% BM in comparison with a control group. In agreement with these results, Brandt et al. [32] reported that a PWL of 10% BM over 29 days increased total mood disturbance between the pre- and post-official weigh-in in national-level male MMA athletes. A possible explanation of this negative cognitive alteration is that extreme PWL procedures that include dehydration-based methods may induce hypohydration, which has been shown to affect brain structure and function [48]. Changes in cerebral perfusion and oxygenation could therefore be involved, although these mechanisms were not assessed in the included studies, with potential implications for the attentional and anticipatory processes that underpin skill execution and decision-making [49].

Taken together, the studies assessing the implications of a 10% BM PWL procedure suggest that it may affect both physical performance and, consequently, training adaptations, as well as the perception of stress and psychophysiological recovery [29]. In this way, stress symptomatology has been related to poorer sport performance, although this relationship was not tested in the included studies. The marked energy restriction involved in PWL may reduce muscle mass and glycogen stores, thereby impairing physical performance and increasing fatigue [50]. Nevertheless, these findings should be interpreted with caution: even in the only study that specified the magnitude of the energy restriction [29], the intervention was neither individualized nor adjusted to the participants’ BM, and the macronutrient distribution was not controlled, all of which are factors that may have influenced the results. In summary, a PWL of 10% BM, even when implemented over longer periods such as 4 weeks, appears to impair sport-specific performance at both the physical and the cognitive level, as well as the psychological state of competitive combat sport athletes.

### 4.2. Effects of Aggressive WL Strategies in Combat Sport Athletes

Regarding aggressive RWL, two studies analyzed the impact of endurance exercise (cycling) over 3 h using plastic clothing in hot conditions (40 °C) [4,37] for reducing 5% BM. In one of them, the control condition was cycling at the same intensity, but in thermoneutral conditions with free consumption of fluids [37]. The first finding was that judokas of the control group did not alter their BM. This allowed the specific effect of exercise-induced dehydration to be isolated, which implied a decreased neuromuscular performance, including a specific neuromuscular performance in a judogi grip strength test and a specific judo combat test (SJFT). A second study aimed to analyze the impact of a RWL of 5% BM after 3 h and after 24 h in comparison with the control group, which cycled in thermoneutral conditions with free consumption of fluids and experienced minimal changes in BM (~1% BM reduction) [4]. That study showed that dehydration decreased handgrip strength and sled push performance (loaded at 75% BM) immediately after the protocol, whereas the medicine ball throw was impaired only 24 h later, a time point at which the sled push performance had also not recovered. A third study, which analyzed a 5% BM reduction achieved over 48 h, reported that SJFT performance after RWL did not differ from baseline but was lower than after the 15 h of recovery allowed by the current weigh-in rule, whereas urine specific gravity remained elevated after recovery in these highly trained judo athletes [44]. Therefore, it is suggested that an aggressive RWL focused on dehydration as the method for reducing BM affects sport performance in sport combat athletes with effects that, in one study, were still present 24 h later [4]. A reduced plasma volume and an altered muscle excitability are plausible mechanisms for both the acute impairment and the delayed recovery, although they were not measured in the included studies (see Section 4.4).

In a longer aggressive RWL protocol (5 days) that included a weight-regain period with fluid and food intake, no effect was observed on a high-intensity interval test (8 × 15 s all-out arm-crank efforts interspersed with 20 s of recovery) in male combat athletes with and without experience in weight loss-and-regain cycles [40]. Similarly, Artioli et al. [36] did not report any effect of a 5% BM reduction achieved over 5 days on either simulated judo combat or repeated upper-body Wingate performance in competitive male judokas, although the authors reported a reduction in resting blood glucose. In contrast, Reljic et al. [42] reported a slightly higher running velocity at 4 mmol·L^−1^ of blood lactate after a 5% BM reduction, although this was accompanied by adverse physiological changes, including decreased erythropoietin and reduced free testosterone.

A standardized reduction of approximately 5.3% BM achieved over 28 to 30 h through energy restriction (22 kcal·kg^−1^ BM) combined with hot water immersion, a method that combines short hot baths (~20 min at ~40 °C) with subsequent wrapping in warm clothing (~40 min) [51], was analyzed in MMA athletes. That study compared two hot water immersions (2 × 20 min separated by 40 min in warm clothing) in salt and in fresh water, followed by a 24 h recovery period with high carbohydrate intake (8 g·kg^−1^) and rehydration (150% of the BM lost), and found no differences in handgrip strength, maximal isometric force, countermovement jump, or high-intensity exercise (3 min all-out test) [38]. Blood urea nitrogen and creatinine concentrations increased after the protocol, reaching values compatible with criteria for acute kidney injury in some participants [52], although they returned to baseline values after recovery [38].

Although Rossi et al. [45] showed that an isolated post-hoc difference in handgrip strength was reported after an aggressive RWL combined with high-intensity sport-specific training, the absence of benefit in the remaining studies, together with the potential physiological and health risks, leads us to advise against aggressive WL procedures in combat sport athletes. Aggressive WL procedures based on dehydration should be avoided in particular, since the impairments were still present 24 h after the protocol in one study [4] and may be accompanied by health risks.

### 4.3. Effects of Moderate WL Strategies in Combat Sport Athletes

Regarding moderate WL, Zubac et al. [43] reported that in international-level male boxers, a 3% BM reduction achieved through RWL decreased the time to exhaustion (16 s) at 70% maximal isometric contraction in leg extension in combination with a 12% lower maximal voluntary isometric contraction (condition effect *p* = 0.001), whereas the reduction in voluntary activation did not reach statistical significance (*p* = 0.058), and the change in the rate of force development of the evoked doublet showed only a time effect. That study also reported an attenuated BLa response after RWL, with the change in maximal voluntary contraction correlating with peak blood lactate in the control condition, suggesting that reduced skeletal muscle glycogen concentrations may affect glycolytic metabolism. Nevertheless, glycogen depletion could contribute to a reduced excitation-contraction coupling [53], with implications for the reduced maximal isometric contraction.

Balance is a complex sensorimotor function depending on muscular, cutaneous, and articular mechanoreceptors, and it is considered a key factor in tactical and technical movements in combat sport athletes [41,54]. Morales et al. [41] found that a moderate (3% BM) RWL without a standardization of the WL protocol negatively affected reaction time and static balance with open and closed eyes. Since impaired balance may compromise perceptual-motor skills in combat sports, moderate RWL could interfere with sport-specific technical and tactical performance in these athletes.

Yoshioka et al. [35] compared national-level judokas who reduced BM over 3 weeks (men, 3.4%; women, 4.9%) with judokas who maintained BM. Significant increases in tension, fatigue, and total mood disturbance and a reduction in vigor were observed only in the men who reduced 3.4% BM, which may reflect a stress response to the energy intake restriction, whereas no significant changes were observed in the women who reduced 4.9% BM. These findings suggest that the psychological response to WL may differ between sexes, although the small number of women (*n* = 8) precludes firm conclusions. A different response was reported in a sample formed by male Greco–Roman Wrestling athletes of international level who reduced 3.5% BM over 23 days with a 15–20% energy intake reduction personalized by a dietitian [31]. In this way, the athletes who followed the personalized WL procedure reduced fatigue and total mood disturbance and increased vigor, while the athletes of the control group, who did not receive any dietary guidelines, increased tension, depression, anger, fatigue, and total mood disturbance. Therefore, it could be suggested that the individualization of the energy restriction by a dietitian could attenuate the psychological cost of a moderate WL procedure.

Analyzing the impact of moderate (3.7% BM) PWL (~20 days), Miranda et al. [34] did not detect any effect on the neuromuscular response in concentric knee flexion/extension and internal/external shoulder rotation at different isokinetic velocities (60º/s and 180º/s). Contraction velocities during isokinetic contractions are indicators of the motor unit pattern of recruitment [55]. Therefore, it could be speculated that PWL does not affect maximal isokinetic strength in movement with recruitment of type I and type II muscle fibers. However, maximal isokinetic strength is considered a potential key in combat sport modalities [56] because performance in combat includes repetitive actions combined with fatigue and uncertainty about the movements of the opponent. In agreement with these results, Koral et al. [33] did not detect any effect of a PWL over approximately 4 weeks (3.9% BM) on high explosive actions as vertical jump ability, and total number of tokui-waza during 5 s, but reported a reduced number of tokui-waza during 30 s in males and females judokas of national and international level. These results strengthen the hypothesis that PWL affects repetitive or longer-duration efforts. Therefore, glycogen depletion could act as a limiting factor [57] and a reduced glycolytic activity, as can be inferred from the lower BLa reported by Zubac et al. [43]. However, the effect of balance on sport-specific skills in combat athletes [41] and the alteration of the mood profile reported in some of the studies [31,35] should also be considered, because mood is a key factor of performance in high-intensity sports that involve open skills, particularly when performance is assessed through self-reference criteria [58]. Nevertheless, although no differences were detected in the total number of tokui-waza during 5 s, caution is needed regarding the impact of a 3% BM reduction in PWL procedures because the characteristics of the test used could make it difficult to find statistically significant differences.

In contrast, the study of Isacco et al. [39] reported a dissociation between physical and psychological outcomes after a 3% BM reduction with RWL. Over five simulated combats, maximal strength in a 30 s isometric rowing test was relatively better preserved in the WL group, which also sustained maximal strength for longer than the control group (11.5 and 9.8 s after the 1st and 5th combats, vs. 3.5 and 3.6 s). However, the WL group reported a higher rating of perceived exertion and a higher perceived training load, together with a less favorable mood profile, with time × group interactions for depression, anger, vigor, fatigue, and confusion after the fourth and fifth combats. Therefore, the relative preservation of force production was obtained at a higher perceived cost. Moreover, force production is not always related to sport performance, which also includes technical-tactical aspects. Finally, the athletes recruited in that study (male national judokas) reported being chronic weight-cyclers, so not changing their dietary habits before competition in the control condition could have affected their performance during the experimental sessions.

The results of the studies included in the systematic review suggest that moderate WL procedures did not impair maximal neuromuscular performance in the lower or the upper body in most studies, with the exception of the reduced maximal voluntary contraction reported after a 3-day RWL [43]; but when the demand of the effort increases, including non-specific and specific (simulated combat) exercise tests, physical performance is progressively diminished. Additionally, in male combat athletes, a decreased mood profile for sport performance was reported after both RWL and PWL, although in the only study in which the energy restriction was personalized by a dietitian, the mood profile was not impaired but improved; however, this pattern of psychological response could be different in females. Additionally, balance is affected negatively. Additionally, it should be considered that energy restriction combined with intense exercise training over time increases creatine kinase concentration, an indicator of risk of injury in the muscle tissue [59]. For that, although the evidence relative to sport performance is not clear in favor of PWL or RWL for moderate WL, the selection of PWL procedures that do not exceed 0.5 to 1.5 kg per week could be suitable for athletes for a more selective reduction of adipose tissue and minimal changes to muscle mass.

### 4.4. Physiological Interpretation of the Observed Effects

The results of the studies included in the systematic review suggest that WL procedures based on dehydration affect sport performance more consistently than WL procedures based on a progressive energy restriction. A possible explanation is that in procedures based on dehydration, the reduction of BM is obtained from body water, first from the extracellular compartment and, when the magnitude of WL is higher, also from the intracellular compartment. In this way, a reduction of 5% BM obtained within a few hours implies a marked decrease in plasma volume, which could increase blood viscosity and reduce stroke volume, requiring a higher heart rate to maintain cardiac output and limiting muscle and cutaneous perfusion and thermoregulation; these cardiovascular responses were not measured in the included studies. Additionally, the loss of intracellular water modifies cell volume, osmolality, and the transmembrane ionic gradients, factors related to the excitability of the sarcolemma, which could contribute to an impaired excitation–contraction coupling, although this was not assessed in the included studies. Nevertheless, it should be considered that the restoration of plasma volume after marked dehydration may require more than 24 h of fluid and sodium intake [5]. Accordingly, the deficits reported in the sled push and the medicine ball throw test were maintained 24 h after rehydration [4], and, after a 5% BM reduction over 48 h, SJFT performance was better after 15 h of recovery than immediately after RWL, although urine-specific gravity remained elevated [44]. In contrast, in PWL procedures, a similar magnitude of WL is distributed over several weeks, so euhydration could be maintained, a higher proportion of the WL corresponds to adipose tissue, and plasma volume and neuromuscular function could be adjusted progressively. Therefore, the physiological cost of PWL procedures may be related less to fluid balance than to substrate availability, which could explain a more selective impairment of performance.

A second finding of the studies included in the systematic review is that the mood profile was affected at magnitudes and time points at which physical performance was still not affected [32,35]. Two explanations could be considered. From a physiological viewpoint, the central nervous system detects the energy deficit before substrate availability becomes a limiting factor for contractile function. Therefore, the reduction in blood glucose, leptin, and triiodothyronine, together with the increased cortisol concentrations and sympathetic activity, are early signals of a negative energy balance that modulate serotonergic and dopaminergic neurotransmission, related to tension, fatigue, vigor, and total mood disturbance [31,35]. Additionally, hypohydration has been shown to affect brain structure and function [48], and changes in cerebral perfusion and oxygenation, which were not measured in the included studies, could affect attentional and anticipatory processes [49] before maximal force production is affected. From a methodological viewpoint, it should be considered that the questionnaires used for assessing mood are continuous and multidimensional, while most of the physical tests used were maximal, of short duration and dependent on the effort of the athlete, so a higher activation and motivation could compensate for the impairment of performance, as it was reported in the study carried out by Isacco et al. [39], in which the WL group relatively preserved maximal strength over five simulated combats but reported a higher perceived exertion and a less favorable mood profile. Therefore, the alteration of the mood profile should be considered an early and sensitive marker of the physiological cost of WL, and not a secondary finding, because mood is a key factor of performance in sport modalities that involve open skills [58].

Regarding moderate WL procedures, the preservation of physical performance reported in several studies could be explained because a reduction lower than 5% BM does not exceed the capacity of the athlete for restoring homeostasis. In this way, when the magnitude of WL does not exceed ~5% BM, the fluid component could be restored within the usual post-weigh-in recovery windows with an adequate intake of fluids and electrolytes [5], muscle glycogen could be resynthesized with a high availability of carbohydrate, and, when the rate of WL does not exceed 0.5 to 1.5 kg per week, a higher proportion of the WL corresponds to adipose tissue with minimal changes in contractile proteins. Additionally, it should be considered that maximal strength and power in a single effort depend mainly on neural drive and on the availability of phosphagens, factors which would not be expected to be substantially affected by a moderate reduction of the glycogen stores [60]. This could explain the absence of effects reported in maximal isokinetic strength, vertical jump ability, and the total number of tokui-waza during 5 s [33,34]. Nevertheless, performance was impaired in tests in which glycolytic metabolism acquires a higher contribution, a terminology used here with the conceptual caveats discussed by Chamari and Padulo for the “aerobic” and “anaerobic” labels [61], or in which the effort is repeated [60], as the time to exhaustion at a submaximal load, in which an attenuated BLa was also reported [43], and the number of tokui-waza during 30 s [33]. Therefore, it is suggested that in moderate WL procedures, the limiting factor is substrate availability and not the contractile or the neural capacity. Nevertheless, the preservation of performance depends on the characteristics of the test and on the time available for recovery, and it is not extended to balance, reaction time, or the mood profile [31,35,41], nor does it imply that the procedure is safe.

### 4.5. Heterogeneity and Certainty of the Evidence

The studies included in this systematic review were markedly heterogeneous across several dimensions that limit the strength of the conclusions. Combat sport disciplines differed substantially in their physiological and technical demands (e.g., judo, wrestling, MMA, boxing, taekwondo). For example, taekwondo alternates high-intensity periods with pauses in a work-to-rest ratio between 1:2 and 1:7, imposing high demands on the lower limbs, in athletes with a somatotype combining moderate musculoskeletal development and relative body linearity [62]; judo matches alternate 20 to 30 s of high-intensity actions with 5 to 10 min of recovery and require greater explosive strength in the upper than in the lower limbs, in athletes with a predominantly mesomorphic somatotype [60]; whereas in MMA, the work-to-rest ratio ranges from 1:2 to 1:4, imposing high demands on both the upper and the lower limbs [63]. Additionally, the combat sport discipline has been shown to shape distinct profiles of perceptual–motor performance [64]. The competitive level of the samples also varied, from national to international athletes, which plausibly modulates the physiological reserve available to tolerate WL. This is relevant because international athletes have been reported to outperform national-level athletes in maximal strength [65], in repeated high-intensity sport-specific tests [66], in tests assessing glycolytic capacity such as the Wingate test [65,67], and in muscular endurance [68], but not in high-velocity neuromuscular power such as vertical jump performance [69]. In this systematic review, sex was unevenly represented (88.7% male), which is consistent with the persistent sex gap reported in sport performance research [70] and in sport nutrition research, including studies on the ergogenic properties of dietary supplements [71]. Female athletes have been shown to display specific responses to dietary interventions [72], and biological and sociological factors have been reported to influence sport performance differently in men and women [73]. Therefore, psychological and possibly physiological responses to WL may differ between sexes. The methods used for reducing BM were also heterogeneous, including exercise in hot conditions with plastic clothing [4,37], saunas, plastic clothing and laxatives [29], hot water immersion [38], and energy restriction [29,31,35], which were frequently combined within the same protocol and, in most of the studies, were neither standardized nor individualized to the characteristics of the athletes, so the relative contribution of dehydration and of the energy deficit to the reported effects cannot be determined. Post-weigh-in recovery windows before outcome assessment varied widely across studies, from immediate post-dehydration testing to assessments performed 24–48 h later, which is a critical source of variability given that several outcomes (e.g., strength, sled-push performance) were shown to only partially recover within this timeframe. Finally, the outcome measures themselves were heterogeneous, ranging from generic strength and power tests to sport-specific instruments (e.g., SJFT, JGST, simulated combats), which are not directly comparable in sensitivity or ecological validity. Given this heterogeneity, the fair average methodological quality (PEDro = 5.30), and the practical challenges of conducting this type of intervention in elite combat sports, where the blinding of participants and of those delivering the intervention (items 5 and 6 of the PEDro scale), is not feasible because athletes must consciously adhere to the WL protocols. Whereas the blinding of outcome assessors (item 7) may be feasible in some designs, confidence in the evidence for most outcomes should be considered limited, an informal judgement based on the risk of bias of the included studies, on the inconsistency of the results reported between studies, and on the imprecision associated with the small samples recruited, being relatively higher for objectively measured physical performance outcomes assessed shortly after WL, and lower for psychological, cognitive, and longer-term recovery outcomes, where fewer studies, smaller samples, and greater methodological variability were observed. Beyond descriptive characterization, the distribution of the available evidence is consistent with the magnitude-based classification adopted in this review. None of the included RWL protocols (≤7 days) achieved a BM reduction ≥10%; extreme-magnitude reductions were reported exclusively under PWL protocols implemented over 2–4 weeks [27,28,29,30,32], suggesting that a 10% BM reduction approximates a practical ceiling for what is achievable within a short timeframe in the reviewed literature. Similarly, aggressive RWL protocols centered around 5% BM were associated, in one study, with performance deficits still present 24 h after the protocol [4], in contrast to moderate WL (<5% BM), for which effects were comparatively inconsistent. Taken together, these patterns suggest that the 5% and 10% cut-offs, although selected a priori for descriptive purposes, coincide with points at which the feasibility of rapid attainment and the persistence of performance impairment change appreciably within the available evidence. These observations should nonetheless be interpreted as hypothesis-generating rather than as confirmed physiological thresholds, given the observational nature of the underlying comparisons. The distribution of null findings across categories was also considered in the synthesis: no report applying extreme WL reported an overall null result, whereas null findings, or findings limited to an isolated favorable outcome, accounted for four of the eight aggressive-WL reports [36,38,40,45] and for one of the seven moderate-WL reports [34]. The proportion of null findings therefore did not decrease consistently with the magnitude of WL, which argues against a simple dose–response interpretation; moreover, most of the studies reporting no significant differences were also among those with the smallest samples and the lowest PEDro scores, so a lack of statistical power cannot be distinguished from a genuine absence of effect. A formal and structured assessment of the certainty of the evidence for each outcome was beyond the scope of the present review and is recommended for future updates of this evidence base. Additionally, it should be considered that the restriction of the search to documents published from 2000 onwards and in English, Spanish, or Portuguese could have excluded eligible studies published before that date or in other languages, so a time and language bias cannot be ruled out. In addition, because deduplication was performed manually rather than with automated tools, the possibility that a duplicate record was inadvertently retained or removed cannot be completely excluded. Finally, the heterogeneity described above is compounded by a markedly uneven distribution of the available evidence across the categories examined. Mapping the 20 included reports onto the magnitude × duration framework (Table 4) shows that the evidence is concentrated in the least severe conditions and that two cells are, in practice, uninformative: no included report examined an aggressive reduction implemented through PWL, since the largest progressive reduction reached 4.9% BM [35], and no study achieved an extreme reduction within ≤7 days. Conversely, the extreme category is supported by five reports that correspond to only three independent samples, since three of them describe a single cohort of 39 male judokas. Accordingly, the comparisons across duration for a given magnitude, and the inferences regarding extreme reductions, rest on a substantially narrower evidence base than the total number of included studies would suggest, and this asymmetry—rather than the number of studies—is the main factor limiting the certainty of the present synthesis.

### 4.6. Practical Applications

A limitation of the present systematic review is that most participants were male combat sport athletes (88.7% of the total sample). Given the well-established sex differences in physiological and psychological responses to exercise [74], as well as in the response to nutritional strategies aimed at optimizing health, performance, and recovery [72], the practical implications of the present findings should be interpreted with caution when applied to women athletes. Accordingly, future research should prioritize investigating sex-specific responses to WL strategies in combat sports.

**Figure 2:**
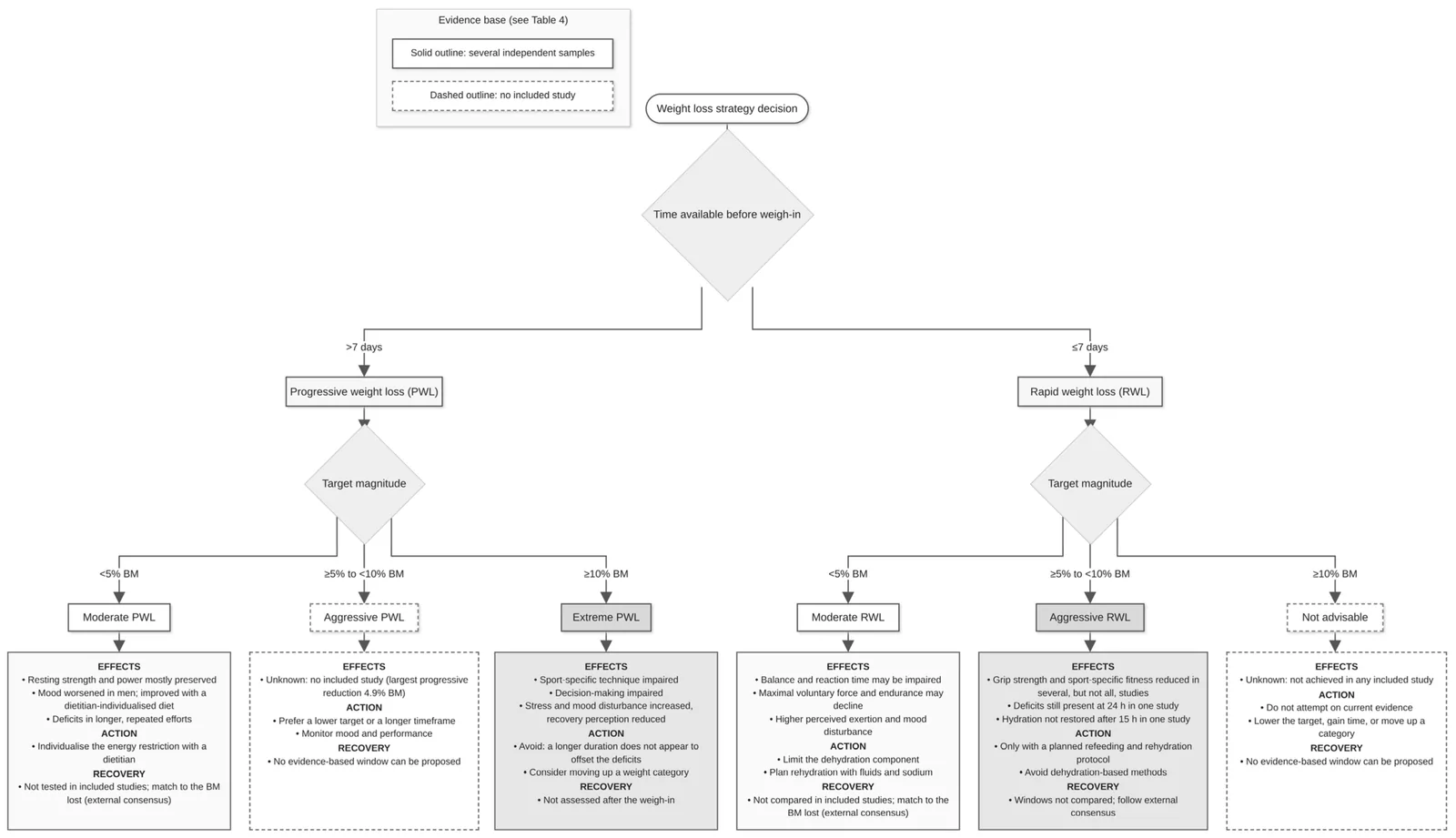
Decision tree for weight loss strategy selection based on time availability and target magnitude in combat sports. Branches are not supported by equivalent evidence (see Table 4): solid outlines indicate cells supported by several independent samples, and dashed outlines indicate cells without evidence from the included studies. No included report examined an aggressive reduction implemented progressively, the extreme PWL branch derives from three independent samples, and no included study achieved a reduction ≥10% BM within ≤7 days; these conditions are presented as evidence gaps rather than as recommendations. Recovery recommendations that were not tested in the included studies derive from external consensus [5]. The framework is therefore provisional and requires validation in future studies.

Despite this limitation, the findings of the present review provide practical guidance for selecting WL strategies according to the time available before competition and the magnitude of BM reduction required to achieve the target weight category. Overall, aggressive RWL protocols compromised physical and sport-specific performance in several but not all studies, and extreme protocols were associated with impaired technical and psychological outcomes, although this evidence originates from a limited number of overlapping PWL cohorts and does not extend to RWL. Based on these findings, a practical decision-making framework (Figure 2) was developed to assist sports nutritionists, dietitians, coaches, and athletes in selecting the most appropriate WL strategy.

## 5. Practical Guidelines for High-Performance Environments

- The evidence synthesized in the present systematic review, together with the proposed decision-making framework (Figure 2), provides several practical recommendations to help optimize weight-class attainment while minimizing performance impairments.
- Prioritize limiting the magnitude of WL. The available evidence suggests that the absolute magnitude of BM reduction may play an important role in performance outcomes. Although PWL may attenuate some of the physiological stress associated with weight reduction, it does not appear sufficient to offset the detrimental effects of extreme reductions (≥10% BM). Therefore, whenever possible, athletes should avoid strategies requiring such drastic BM reductions.
- Moderate WL (<5% BM). Both PWL and RWL may be appropriate for moderate reductions in BM, provided that the time available for post-weigh-in rehydration and nutritional recovery is sufficient for the magnitude of BM lost. Under these circumstances, the primary objectives should be restoring fluid balance, replenishing glycogen stores, and minimizing psychological stress through adequate nutritional planning.
- Aggressive WL (≥5% to <10% BM). Within the included studies, deficits in sled-push and medicine ball throw performance were still present 24 h after an aggressive dehydration protocol in one study [4], whereas in another study, the SJFT index improved after 15 h of recovery despite a persistently elevated urine specific gravity [44]; no included study compared different recovery windows directly. Based on broader sports nutrition consensus outside this review, the magnitude of the acute BM loss should be matched to the recovery time actually available after the weigh-in, prioritizing plasma volume restoration and neuromuscular recovery [5]. During this period, external guidelines suggest that high carbohydrate intake (e.g., ~5–10 g·kg^−1^·day^−1^), and individualized rehydration protocols should be prioritized [5,13], with particular attention when the weigh-in and the competition take place under hot ambient conditions [75].
- Extreme WL (≥10% BM). Extreme WL should be avoided whenever possible. The reviewed PWL evidence indicates that reductions of this magnitude frequently compromise technical skill execution, decision-making, and mood state. Notably, there is a lack of empirical evidence regarding extreme RWL (≥10% BM in ≤7 days) in the current review, highlighting the severe difficulty and potential health risks associated with attempting such acute mass reductions.
- Female athletes. Given that approximately 88% of participants included in the available literature are male, these recommendations should be extrapolated to female athletes with caution. Further research is required to determine how menstrual cycle phase and sex-specific physiological responses influence the effectiveness and safety of different WL strategies.

## 6. Conclusions

The findings of this systematic review based on 20 reports from 18 independent intervention studies of predominantly fair methodological quality suggest that the effects of WL on combat sport performance may be influenced by the magnitude of BM reduction, although duration and magnitude were not compared experimentally and, as Table 4 shows, several magnitude × duration combinations are supported by very limited or no evidence. While moderate WL strategies (<5% BM) yielded heterogeneous findings, with one of the seven reports showing no significant effects, four reporting significant impairments, one a more favorable mood profile, and one mixed findings, aggressive protocols (≥5% to <10% BM) yielded mixed results, with impairments of physical and sport-specific performance reported in several but not in all of the studies. Extreme protocols (≥10% BM) were associated with impaired technical skills and a less favorable mood profile, even when implemented progressively, although this observation is based on a small number of studies that applied PWL protocols exclusively.

The proposed decision-making framework is presented as a provisional practical tool to guide the selection of WL strategies, which should be validated in future studies. Nevertheless, successful implementation requires sufficient time for post-weigh-in recovery. Additionally, the available evidence, which for extreme reductions was restricted to PWL protocols, suggests that extending the duration of WL does not seem to fully mitigate the detrimental effects associated with extreme reductions (≥10% BM).

These conclusions must be interpreted with caution due to the limited number of studies included, their predominantly fair methodological quality, and the substantial heterogeneity in combat sport disciplines, testing protocols, and recovery windows. Finally, the current evidence base lacks representation of extreme RWL protocols and is heavily dominated by male athletes, highlighting the need for future research. Priority should be given to the conditions for which evidence is currently absent or derived from a single cohort, namely reductions ≥10% BM, which derive from three independent samples, and aggressive reductions implemented progressively, for which no included report provides evidence, and to designs that manipulate magnitude and duration within the same sample so that their combined effect can be estimated rather than inferred across studies. Female athletes, disciplines other than judo, recovery windows longer than 24 h, and the long-term health consequences of repeated weight cycling remain equally under-represented.

## Figures and Tables

**Table 1:** PICOS criteria for the inclusion of studies in the systematic review.

Parameter	Criteria
Population	Combat sport athletes
Intervention	Programmed strategies for WL, including RWL and PWL, of the next magnitudes: (i) <5% BM; (ii) ≥5% to <10% BM; (iii) ≥10% BM
Comparison	<5% BM vs. ≥5% to <10% BM vs. ≥10% BM vs. control (maintenance of BM)
Outcomes	Variables related to combat sport performance
Setting	Controlled intervention studies (randomized or non-randomized), including crossover and parallel-group designs; blinding of participants and/or outcome assessors was not required as an eligibility criterion given its limited feasibility in exercise and sports nutrition interventions

**Table 2:** Summary of the studies included in the systematic review.

Cite	Sample	Experimental Conditions	Weight Loss Protocol	Outcomes	Results
De Sousa et al., 2017 [30]	31 Male taekwondo athletes at the national level	EG1 (*n* = 15): PWL (10% BM) over 2 weeks EG2 (*n* = 16): control	EG1: 5% BM reduction weekly (no standardization of WL protocol) EG2: maintenance of BM	Pre- and post- intervention: - SEI on a three-round combat	SEI: - Total score: ↑ EG2 in post (19.51%; from 0.41 to 0.49; *p* = 0.01) Group × time interaction (*p* = 0.001) - Round 2 post: ↑ EG2 vs. EG1 (27.5%; 0.51 vs. 0.40; *p* = 0.02) ↓ EG1 Round 2 vs. EG1 Round 1 in post (11.11%; 0.45 vs. 0.40; *p* = 0.03) - Round 3: ↑ EG2 vs. EG1 (14.28%; 0.48 vs. 0.42; *p* = 0.01)
Fortes et al., 2019 [29]	39 Male judokas at the national level	EG1 (*n* = 20): PWL (10% BM) over 2 weeks EG2 (*n* = 19): control	EG1: 5% BM reduction weekly based on an energy reduction (6.7 MJ/day) combined with other methods (plastic clothes during training, laxatives and saunas) EG2: maintenance of BM	Pre- and post-intervention: - DALDA - RESTQ-Sport	- DALDA (part B): ↑ EG1 in post (15.43%; from 34.23 to 39.51; *p* = 0.01) ↑ EG1 vs. EG2 (15.56%; 39.51 vs. 34.19; *p* = 0.01) - RESTQ-Sport: ↓ EG1 vs. EG2 (24.73%; 87.63 vs. 109.30; *p* = 0.001) ↓ EG1 in post (13.58%; from 101.40 to 87.63; *p* = 0.001) ↑ EG2 in post (8.25%; from 100.97 to 109.30; *p* = 0.01)
De Sousa et al., 2017 [27]	39 Male judokas at the national level	EG1 (*n* = 20): PWL (10% BM) over 2 weeks EG2 (*n* = 19): control	EG1: 5% BM reduction weekly (no standardization of WL protocol) EG2: maintenance of BM	Pre- and post-intervention: - GPAI	GPAI: - Skill execution index: ↑ EG2 in post (10.64%; from 0.47 to 0.52; *p* = 0.01) Group × time interaction (*p* = 0.01)
Fortes et al., 2017 [28]	39 Male judokas at the national level	EG1 (*n* = 20): PWL (10% BM) over 2 weeks EG2 (*n* = 19): control	EG1: 5% BM reduction weekly (no standardization of WL protocol) EG2: maintenance of BM	Pre- and post-intervention: - SJFT	- Total throws: ↓ EG1 in post (5.91%; from 20.3 to 19.1; *p* = 0.01) ↑ EG2 in post (5%; from 20.0 to 21.0; *p* = 0.01). Group × time interaction (*p* = 0.001) - SJFT index: ↓ EG2 in post (5.34%; from 13.1 to 12.4; *p* = 0.01) Group × time interaction (*p* < 0.01)
Morales et al., 2018 [41]	38 Judokas at the national level (males: 26; females: 12)	EG1 (*n* = 16): RWL (3% BM) over 48 h EG2 (*n* = 13): PWL (3% BM) over >1 week EG3 (*n* = 9): control	EG1: 3% BM reduction over 48 h (no standardization of WL protocol) EG2: 3% BM reduction in >1 week (no standardization of WL protocol) EG3: maintenance of BM	Pre- and post-intervention: - Trunk strength - Handgrip strength - Reaction time - Balance test (open and closed eyes)	- Reaction time: ↑ EG1 in post (*p* < 0.05) - Balance test (open eyes): Anteroposterior Velocity: ↑ EG1 in post (*p* < 0.05) - Balance test (close eyes): Ellipse area: Group × time interaction (*p* = 0.023) ↑ EG1 (*p* < 0.05) - Anteroposterior Velocity: ↑ EG1 in post (*p* < 0.05) Group × time interaction (*p* = 0.042) - Mediolateral velocity: ↑ EG1 in post (*p* < 0.05) Group × time interaction (*p* = 0.027)
Isacco et al., 2020 [39]	20 Male judokas at the national level	EG1 (*n* = 10): RWL (3% BM) over 1 week EG2 (*n* = 10): control	EG1: reduction based on an energy intake reduction (no standardization of WL protocol) EG2: maintenance of BM	Pre- and post-intervention (Post-5 simulated combat interspersed 30 min): - Perceived training load (combat duration × RPE) - RPE - Handgrip strength - 30 s horizontal isometric rowing test - POMS	- Perceived training load: ↑ EG1 vs. EG2 (group effect *p* < 0.05) Group × time interaction at post-combat 5 (*p* < 0.001) 30-s horizontal isometric rowing test: - RPE: ↑ EG1 vs. EG2 (group effect *p* < 0.05) Group × time interaction at post-combat 4 (*p* < 0.05) and post-combat 5 (*p* < 0.0001) - Maximal strength: Group × time interaction at post-combat 4 and post-combat 5 (*p* < 0.05), with maximal strength better preserved in EG1. - Time sustained at maximal strength: longer in EG1 than in EG2 (group effect *p* = 0.001); group × time interaction at post-combat 3 (*p* < 0.01), post-combat 4 (*p* < 0.001) and post-combat 5 (*p* < 0.001) - Hand grip strength (left hand): Group × time interaction at post-combat 5 (*p* = 0.04) - Hand grip strength (right hand): group × time interaction at post-combat 5 (*p* = 0.05) 5 simulated combats: - POMS: Group × time interaction for depression, anger, vigour, fatigue, and confusion at post-combat 4 and post-combat 5 (*p* < 0.05), with a less favorable mood profile in EG1
Barley et al., 2018 [4]	14 Male MMA fighters at the professional and amateur level	EC1: RWL (5% BM) over 3 h EC2: control	EC1: reduction based on cycling (60 W) in hot conditions (~40 °C) with plastic sweat suit and deprivation of hydration EC2: ~1% BM reduction based on cycling (60 W) in thermoneutral conditions (25 °C) with free consumption of fluids	Pre-3, post-3 h and post-24 h intervention: - Handgrip strength - Sled push test (75% BM) - Medicine ball throw - Vertical jump	- Hand grip strength: ↓ EC1 vs. EC2 in post-3h (3.9%; 51 ± 8 vs. 53 ± 8 kg; *p* = 0.044) - Sled push test (75% BM): ↓ EC1 vs. EC2 (slower mean effort time) in post-3 h (≈19% slower; 6.68 ± 1.48 vs. 5.23 ± 0.66 s; *p* = 0.001) and post-24h (≈14% slower; 5.94 ± 1.42 vs. 5.15 ± 0.77 s; *p* = 0.012) - Medicine ball chest throw: ↓ EC1 vs. EC2 in post-24 h (5.3%; 449 ± 44 vs. 474 ± 52 cm; *p* = 0.016)
Reljic et al., 2016 [42]	28 Male combat athletes at the regional, national, and international levels (boxers: 6; wrestlers: 13; taekwondoists: 3; karatekas 1; judo: 5)	EG1 (*n* = 14): RWL (5% BM) over 1 week EG2 (*n* = 14): control	EG1: no standardization of WL protocol EG2: maintenance of BM	Ordinary training period (T1)—pre-competition (T2)—post-competition (T3): —Running GET	- Running GET in T2: ↑ EC1 vs. Pre velocity at 4 mmol of BLa (4.6%; from 13.1 to 13.7 km/h; *p* < 0.05)
Ceylan et al., 2022 [37]	9 Male judokas at the international level	EC1 (*n* = 9): RWL (5% BM) over 3 h EC2 (*n* = 9): control	EG1: reduction based on cycling (60 W) in a sauna (40 °C and 40% relative humidity) over 3 h EG2: cycling (60 W) in thermoneutral conditions with free consumption of fluids	- Handgrip strength - SJFT - JGST	- Hand grip strength (right hand): ↓ EC1 vs. EC2 (−4.0%; 47.6 ± 3.8 vs. 49.5 ± 4.5 kg; *p* = 0.004) - Hand grip strength (left hand): ↓ EC1 vs. EC2 (−4.4%; 45.7 ± 7.4 vs. 47.8 ± 4.6 kg; *p* = 0.037) - SJFT (total throws): ↓ EG1 in set 1 (−8.0%; *p* < 0.05) and set 3 (−7.9%; *p* < 0.05) - JGST (isometric): ↓ EC1 vs. EC2 (%; 45.8 ± 10.6 vs. 53.6 ± 11.5 s; *p* < 0.001) - JGST (dynamic): ↓ EC1 vs. EC2 (−23.8%; 16 ± 4 vs. 21 ± 3 repetitions; *p* = 0.005)
Brandt et al., 2018 [32]	12 Male MMA fighters at the national level	EG1 (*n* = 9): PWL (10% BM) over 29 days EG2 (*n* = 3): control	EG1: reduction based on an energy intake reduction (50 g/day) combined over the last 24 h with other methods (warmer clothes during training, hot baths, and sauna) EG2: maintenance of BM	Pre- (30 days), official weight in, pre-competition and post-competition: - BRUMS	- Total mood disturbance: ↑ EG1 in official weight in (*p* = 0.008), pre-competition (*p* = 0.008) and post-competition (*p* = 0.011)
Koral et al., 2009 [33]	20 Judokas of national or international level (males: 10; females: 10)	EG1 (*n* = 10): PWL (4% BM) over ~4 weeks EG2 (*n* = 10): control	EG1: reduction based on an energy intake reduction (4 MJ/day) personalized by a dietitian combined with a plastic sweat suit in 6 judo training (2–3 h) and conditioning sessions (45 min) during the last 6 days (≈4 weeks in total) EG2: maintenance of BM	Pre- and post- intervention - CMJ - SJ - Number of tokui-waza during 5 s - Number of tokui-waza during 30 s	Number of tokui-waza during 30 s: ↓ EG1 in post in males (−11.9%; from 48.4 ± 5.4 to 42.6 ± 5.1; *p* < 0.01) and females (−8.0%; from 40.0 ± 2.3 to 36.8 ± 3.8; *p* < 0.01) Group × time interaction (*p* < 0.01)
Connor et al., 2022 [38]	13 Male MMA fighters of competitive level	EC1: RWL (~5.3% BM) over ~28–30 h (FWB) EC2: RWL (~5.3% BM) over ~28–30 h (SWB)	EG1: reduction based on hot water immersion using freshwater bathing combined with an energy intake reduction (22 kcal/kg) and hydration (15 mL/kg) EG2: hot water immersion using saltwater bathing with an energy intake reduction (22 kcal/kg) and hydration (15 mL/kg)	Pre-, post-24h intervention and post-24h RWG: - Handgrip strength - Maximal isometric mid-thigh pull - CMJ - 3 min all-out cycle ergometer exercise	It was not detected any statistical differences
Ceylan et al., 2022 [44]	18 Male judokas at the national and international level	EG1 (*n* = 9): RWL (5% BM) over 2 days EG2 (*n* = 9): control	EG1: no standardization of WL protocol EG2: maintenance of BM	Baseline (48 h before the simulated weigh-in), after RWL (simulated weigh-in) and after 15-h RWG: - SJFT	- Total throws: ↓ EG1 after RWL vs. after 15-h RWG (−6.0%; 79 vs. 84 throws summed over three consecutive SJFTs; *p* < 0.05); no difference vs. baseline - SJFT index: Higher (worse) index in EG1 after RWL vs. after 15-h RWG (*p* < 0.05)
Mendes et al., 2013 [40]	21 Male combat athletes (judokas: 12; Jiu-Jitsu: 2; MMA: 5; wrestlers: 2)	Pre- and post- intervention EG1 (*n* = 10): weight cyclers, RWL (≤5% BM) over 5 days EG2 (*n* = 8): non-weight cyclers, RWL (≤5% BM) over 5 days	EG2: no standardization of WL protocol EG1: no standardization of WL protocol	- 8 × 15 s all-out arm–crank exercise interspersed over 20 s	It was not detected any statistical differences
Rossi et al., 2022 [45]	10 Male Greco- Roman wrestlers at the national level	EC1: baseline EC2: RWL (5% BM) over 3 days EC3: 7 days after EC2, without further weight loss	EC1: maintenance of BM EC2: no standardization of WL protocol EC3: without any guidelines	- Handgrip strength	- Handgrip strength (left hand): ↑ EC2 vs. EC3 (6.7%; 57.6 ± 4.4 vs. 54.0 ± 4.0 kg; *p* = 0.039)
Miranda et al., 2021 [34]	8 Wrestlers at the national or international level (males: 5; females: 3)	EC1: baseline EC2: PWL (3.7% BM) over ~20 days	EC1: maintenance of BM EC2: no standardization of WL protocol over ~20 days	- Concentric knee extension (60º/s and 180º/s) - Concentric knee flexion (60º/s and 180º/s) - Concentric internal shoulder rotation (60º/s and 180º/s) - Concentric external shoulder rotation (60º/s and 180º/s)	It was not detected any statistical differences
Yoshioka et al., 2006 [35]	43 Judokas at the national level (males: 27; females: 16)	EG1 (*n* = 22): males PWL (3.4% BM) over 3 weeks EG2 (*n* = 5): males control EG3 (*n* = 8): females PWL (4.9% BM) over 3 weeks EG4 (*n* = 8): female control	EG1: 3.4% BM reduction based on exercise with and without a plastic sweat suit, caloric restriction, and fluid restriction personalized by a coach EG2: maintenance of BM EG3: 4.9% BM reduction with the same procedure that EG1 EG4: The same that EG2	Pre- and post- intervention: - POMS	Fatigue: ↑ EG1 in post (43.8%; from 5.7 ± 4.1 to 8.2 ± 6.0; *p* < 0.01) Tension: ↑ EG1 in post (12.6%; from 9.5 ± 3.9 to 10.7 ± 5.7; *p* < 0.05) Vigor: ↓ EG1 in post (−21.9%; from 9.6 ± 4.7 to 7.5 ± 4.9; *p* < 0.05) Total mood disturbance: ↑ EG1 in post (9.4%; from 122.6 ± 14.6 to 134.1; *p* < 0.05)
Zubac et al., 2020 [43]	9 Male boxers at the international level	EC1: RWL (3% BM) over 3 days EC2: control	EC1: reduction based on an energy intake reduction and hydration (no standardization of WL protocol) EG2: maintenance of BM	Pre- and post-fatiguing test (TTE at 70% MIC): - Voluntary MIC - Induced MIC (20 Hz and 80 Hz)	TTE at 70% MIC: - Time: ↓ EC1 vs. EC2 (*p* = 0.020) Voluntary MIC: - Force: ↓ EC1 vs. EC2 (condition effect *p* = 0.001; greater MVC loss in EC1, post hoc *p* = 0.001) Resting doublet (100 Hz), peak force: Greater decline in EC1 (post hoc *p* = 0.050; interaction tested at *p* < 0.10) Induced MIC (20 Hz): - RFD: Group × time interaction (*p* = 0.090; significant only at the *p* < 0.10 threshold used by the authors)
Artioli et al., 2010 [36]	14 Male judokas at the competitive level	EG1 (*n* = 7): RWL (5% BM) over 5 days EG2 (*n* = 7): control	EG1: no standardization of WL protocol EG2: maintenance of BM	- 3 × uchi-komi Simulated judo combat - 3 × upper body Wingate	3 × upper body Wingate: - Absolute total work: ↑ EG1 in post (*p* < 0.01) ↑ EG2 in post (*p* < 0.01)-Relative total work: ↑ EG1 in post (*p* < 0.01) ↑ EG2 in post (*p* < 0.01)
Ayar et al., 2023 [31]	16 Male Greco–Roman Wrestling at the international level	EG1 (*n* = 8): PWL (3.5% BM) over 23 days EG2 (*n* = 8): control (1.6% BM) over 23 days	EG1: 15–20% energy intake reduction personalized by a dietitian EG2: without any guidelines	Pre- and post-intervention: - POMS	- Tension: ↑ EG2 in post (60.6%; from 11.13 to 17.88; *p* = 0.012) Group × time interaction (*p* = 0.001) - Depression: ↑ EG2 in post (109.3%; from 9.38 to 19.63; *p* = 0.012) Group × time interaction (*p* = 0.002) - Anger: ↑ EG2 in post (140.8%; from 9.50 to 22.88; *p* = 0.012) Group × time interaction (*p* = 0.001) - Fatigue: ↓ EG1 in post (−35.5%; from 5.63 to 3.63; *p* = 0.041) ↑ EG2 in post (98.3%; from 7.63 to 15.13; *p* = 0.012) Group × time interaction (*p* = 0.001) - Vigor: ↑ EG1 in post (15.4%; from 18.75 to 21.63; *p* = 0.021) - Total mood disturbance: ↓ EG1 in post (−65.9%; from 34.50 to 11.75; *p* = 0.017) ↑ EG2 in post (151.1%; from 28.63 to 71.88; *p* = 0.012) Group × time interaction (*p* = 0.001)

Abbreviations: BM: body mass; BRUMS: Brunel Mood Scale; CMJ: countermovement jump; CRT: two-choice reaction/movement-time test; DALDA: Daily analysis profile demands in athletes; EC: experimental condition; EG: experimental group; FTP: functional threshold power; FWB: Fresh Water Bathing; GET: gradual exercise test; GPAI: Game Performance Assessment Instrument; HGS: handgrip strength; IMTP: Isometric mid-thigh pull; JGST: Judogi Grip Strength Test; MIC: maximal isometric contraction; MHST: Maximal Handgrip Strength Test; MMA: mixed martial arts; MP: mean power; MVC: Maximal voluntary contraction; PANAS: Positive and Negative Affect Schedule; PF: Punching force; POMS: Profile of Mood States; PT: Peak torque; RESTQ-Sport: Recovery-Stress Questionnaire for Athletes; RPE: the rating of perceived exertion; PWL: Progressive Weight Loss > 7 days; RWG: rapid weight gain; RWL: Rapid Weight Loss ≤ 7 days; SEI: skill execution index; SJ: squat jump; SJFT: Special judo fitness test; SWB: Salt Water Bathing; TTE: Time-To-Exhaustion; TW-30: tokui-waza during 30 s.; TW-5: tokui-waza during 5 s; WL: Weight Loss; s: seconds.

**Table 3:** Quality of the studies included in the systematic review according to PEDro scale.

Cite	1	2	3	4	5	6	7	8	9	10	11	Total
De Sousa et al., 2017 [30]	YES	YES	YES	YES	NO	NO	NO	YES	YES	YES	YES	7
Fortes et al., 2019 [29]	YES	YES	NO	YES	NO	NO	NO	YES	YES	YES	YES	6
De Sousa et al., 2017 [27]	YES	YES	YES	YES	NO	NO	NO	YES	YES	YES	YES	7
Fortes et al., 2017 [28]	YES	YES	NO	YES	NO	NO	NO	YES	YES	YES	YES	6
Morales et al., 2018 [41]	YES	YES	NO	YES	NO	NO	NO	YES	YES	YES	YES	6
Isacco et al., 2020 [39]	YES	YES	NO	YES	NO	NO	NO	YES	YES	YES	YES	6
Barley et al., 2018 [4]	YES	YES	NO	YES	NO	NO	NO	YES	YES	YES	YES	6
Reljic et al., 2016 [42]	YES	NO	NO	YES	NO	NO	NO	YES	YES	YES	YES	5
Ceylan et al., 2022 [37]	YES	NO	NO	YES	NO	NO	NO	YES	YES	YES	YES	5
Brandt et al., 2018 [32]	YES	NO	NO	NO	NO	NO	NO	YES	YES	YES	YES	4
Koral et al., 2009 [33]	YES	NO	NO	YES	NO	NO	NO	YES	YES	YES	YES	5
Connor et al., 2022 [38]	YES	YES	NO	YES	NO	NO	NO	YES	YES	YES	YES	6
Ceylan et al., 2022 [44]	YES	NO	NO	YES	NO	NO	NO	YES	YES	YES	YES	5
Mendes et al., 2013 [40]	YES	NO	NO	YES	NO	NO	NO	YES	YES	YES	YES	5
Rossi et al., 2022 [45]	YES	NO	NO	NO	NO	NO	NO	YES	YES	YES	YES	4
Miranda et al., 2021 [34]	YES	NO	NO	NO	NO	NO	NO	YES	YES	YES	YES	4
Yoshioka et al.,2006 [35]	YES	NO	NO	NO	NO	NO	NO	YES	YES	YES	YES	4
Zubac et al., 2020 [43]	YES	YES	NO	YES	NO	NO	NO	YES	YES	YES	YES	6
Artioli et al., 2010 [36]	YES	NO	NO	NO	NO	NO	NO	YES	YES	YES	YES	4
Ayar et al., 2023 [31]	YES	NO	NO	NO	YES	NO	NO	YES	YES	YES	YES	5

**Table 4:** Distribution of the available evidence across the weight loss magnitude × duration framework.

WL Magnitude	PWL (>7 Days)	RWL (≤7 Days)
Moderate (<5% BM)	6 conditions/6 independent samples [31,33,34,35,41]	3 conditions/3 independent samples [39,41,43]
Aggressive (≥5% to <10% BM)	No study	8 conditions/8 independent samples [4,36,37,38,40,42,44,45] a
Extreme (≥10% BM)	5 reports/3 independent samples [27,28,29,30,32]	No study

Cells report the number of experimental conditions and the number of independent participant samples. Conditions rather than publications are counted because two studies contributed more than one WL arm: Yoshioka et al. [35] applied reductions of 3.4% BM in men and 4.9% BM in women over 3 weeks (both moderate PWL), and Morales et al. [41] applied a 3% BM reduction over 48 h (moderate RWL) and over more than 1 week (moderate PWL) in different athletes. Connor et al. [38] is counted as aggressive RWL according to the reduction achieved (≈5.3% BM over 28–30 h); its two hot-water immersion conditions were applied to the same athletes and are counted once. Three reports [27,28,29] describe a single cohort of 39 male judokas and are counted as one sample; the extreme cell is expressed as reports to make that overlap explicit. Rossi et al. [45] applied a 5% BM reduction over 3 days, followed by a 7-day phase without further WL; only the WL phase is counted. BM, body mass; PWL, progressive weight loss; RWL, rapid weight loss; WL, weight loss.

## Data Availability

The data extracted from the included studies are provided in the article and in Supplementary Tables S1–S3. Further information is available from the corresponding author upon reasonable request.

## Supplementary Materials

The following supporting information can be downloaded at: https://www.mdpi.com/article/10.3390/nu18213474/s1. Table S1: PRISMA 2020 checklist; Table S2: Complete database-specific search strategies in each database used; Table S3: Outcome-level results of all eligible outcomes reported in the included studies, significant or not.

## Abbreviations

The following abbreviations are used in this manuscript:

BLa	Blood Lactate concentration
BM	Body Mass
BMI	Body Mass Index
BRUMS	Brunel Mood Scale
CHO	Carbohydrates
CMJ	Countermovement Jump
CRT	Two-choice reaction/movement-time test
DALDA	Daily Analysis of Life Demands in Athletes
EC	Experimental Condition
EG	Experimental Group
FWB	Fresh Water Bathing
GET	Gradual Exercise Test
GPAI	Game Performance Assessment Instrument
HGS	Handgrip Strength
IMTP	Isometric Mid-thigh Pull
JGST	Judogi Grip Strength Test
LEA	Low Energy Availability
MHST	Maximal Handgrip Strength Test
MIC	Maximal Isometric Contraction
MMA	Mixed Martial Arts
MP	Mean Power
MVC	Maximal Voluntary Contraction
PANAS	Positive and Negative Affect Schedule
PEDro	Physiotherapy Evidence Database scale
PF	Punching Force
PICOS	Population, Intervention, Comparison, Outcomes, and Setting
POMS	Profile of Mood States
PWL	Progressive Weight Loss > 7 days
RESTQ-Sport	Recovery-Stress Questionnaire for Athletes
RPE	Rating of Perceived Exertion
RWG	Rapid Weight Gain
RWL	Rapid Weight Loss ≤ 7 days
SEI	Skill Execution Index
SJ	Squat Jump
SJFT	Special Judo Fitness Test
SWB	Salt Water Bathing
TTE	Time-To-Exhaustion
TW-5	Tokui-waza during 5 s
TW-30	Tokui-waza during 30 s
WL	Weight Loss
