# Supplementary material for: Impact of the Magnitude and Duration of Weight Loss Procedures in Combat Sports Athletes: A Systematic Review
Source: Nutrients. 2026 Oct 22;18(21):3474. doi: 10.3390/nu18213474 (PMC13765038; doi:10.3390/nu18213474)
Supplement: Supplementary file 1 [file nutrients-18-03474-s001.zip › nutrients-4500221-supplementary.pdf]

Supplementary Table S1. PRISMA 2020 checklist

Section and Topic	Item #	Checklist item	Location where item is reported
		**TITLE**	
Title	1	Identify the report as a systematic review.	p.1
		**ABSTRACT**	
Abstract	2	See the PRISMA 2020 for Abstracts checklist.	p.1
		**INTRODUCTION**	
Rationale	3	Describe the rationale for the review in the context of existing knowledge.	p.3–4
Objectives	4	Provide an explicit statement of the objective(s) or question(s) the review addresses.	p.4
		**METHODS**	
Eligibility criteria	5	Specify the inclusion and exclusion criteria for the review and how studies were grouped for the syntheses.	Section 2.2 + Table 1
Information sources	6	Specify all databases, registers, websites, organisations, reference lists and other sources searched or consulted to identify studies. Specify the date when each source was last searched or consulted.	Section 2.3 (databases, language and date restrictions, last search on 30 September 2025, deduplication procedure) + Supplementary Table S1
Search strategy	7	Present the full search strategies for all databases, registers and websites, including any filters and limits used.	Section 2.3 + Supplementary Table S1
Selection process	8	Specify the methods used to decide whether a study met the inclusion criteria of the review, including how many reviewers screened each record and each report retrieved, whether they worked independently, and if applicable, details of automation tools used in the process.	Section 2.3 (independent screening by two

Section and Topic	Item #	Checklist item	Location where item is reported
			reviewers, third reviewer for discrepancies)
Data collection process	9	Specify the methods used to collect data from reports, including how many reviewers collected data from each report, whether they worked independently, any processes for obtaining or confirming data from study investigators, and if applicable, details of automation tools used in the process.	Section 2.4
Data items	10a	List and define all outcomes for which data were sought. Specify whether all results that were compatible with each outcome domain in each study were sought (e.g. for all measures, time points, analyses), and if not, the methods used to decide which results to collect.	Section 2.4
	10b	List and define all other variables for which data were sought (e.g. participant and intervention characteristics, funding sources). Describe any assumptions made about any missing or unclear information.	Section 2.4
Study risk of bias assessment	11	Specify the methods used to assess risk of bias in the included studies, including details of the tool(s) used, how many reviewers assessed each study and whether they worked independently, and if applicable, details of automation tools used in the process.	Section 2.5
Effect measures	12	Specify for each outcome the effect measure(s) (e.g. risk ratio, mean difference) used in the synthesis or presentation of results.	Section 2.6
Synthesis methods	13a	Describe the processes used to decide which studies were eligible for each synthesis (e.g. tabulating the study intervention characteristics and comparing against the planned groups for each synthesis (item #5)).	Section 2.6
	13b	Describe any methods required to prepare the data for presentation or synthesis, such as handling of missing summary statistics, or data conversions.	Sections 2.4 and 2.6
	13c	Describe any methods used to tabulate or visually display results of individual studies and syntheses.	Section 2.6
	13d	Describe any methods used to synthesize results and provide a rationale for the choice(s). If meta-analysis was performed, describe the model(s), method(s) to identify the presence and extent of statistical heterogeneity, and software package(s) used.	Section 2.6
	13e	Describe any methods used to explore possible causes of heterogeneity among study results (e.g. subgroup analysis, meta-regression).	Not applicable (no formal exploration of heterogeneity was planned);

Section and Topic	Item #	Checklist item	Location where item is reported
			sources of heterogeneity are discussed narratively in Section 4.5
	13f	Describe any sensitivity analyses conducted to assess robustness of the synthesized results.	Not applicable (no sensitivity analyses were performed)
Reporting bias assessment	14	Describe any methods used to assess risk of bias due to missing results in a synthesis (arising from reporting biases).	Not applicable
Certainty assessment	15	Describe any methods used to assess certainty (or confidence) in the body of evidence for an outcome.	Sections 2.6 and 4.5. No formal or structured certainty assessment was performed; only an informal judgement of confidence in the evidence is reported
		**RESULTS**	
Study selection	16a	Describe the results of the search and selection process, from the number of records identified in the search to the number of studies included in the review, ideally using a flow diagram.	Section 3.1 + Figure 1
	16b	Cite studies that might appear to meet the inclusion criteria, but which were excluded, and explain why they were excluded.	Section 3.1 + Figure 1 (number of reports excluded at each stage and reasons for exclusion)

Section and Topic	Item #	Checklist item	Location where item is reported
Study characteristics	17	Cite each included study and present its characteristics.	Section 3.2 + Table 2 + Supplementary Table S3
Risk of bias in studies	18	Present assessments of risk of bias for each included study.	Section 3.7 + Table 3
Results of individual studies	19	For all outcomes, present, for each study: (a) summary statistics for each group (where appropriate) and (b) an effect estimate and its precision (e.g. confidence/credible interval), ideally using structured tables or plots.	Table 2 + Supplementary Table S3
Results of syntheses	20a	For each synthesis, briefly summarise the characteristics and risk of bias among contributing studies.	Sections 3.3–3.6 and 4.1–4.4
	20b	Present results of all statistical syntheses conducted. If meta-analysis was done, present for each the summary estimate and its precision (e.g. confidence/credible interval) and measures of statistical heterogeneity. If comparing groups, describe the direction of the effect.	Sections 3.3–3.6 and 4.1–4.4 (narrative synthesis; no meta-analysis was performed, see Section 2.6)
	20c	Present results of all investigations of possible causes of heterogeneity among study results.	Section 4.5 (heterogeneity across disciplines, protocols, competitive level and recovery windows)
	20d	Present results of all sensitivity analyses conducted to assess the robustness of the synthesized results.	Not applicable
Reporting biases	21	Present assessments of risk of bias due to missing results (arising from reporting biases) for each synthesis assessed.	Not applicable. No formal assessment of reporting bias was performed;

Section and Topic	Item #	Checklist item	Location where item is reported
			time and language bias are discussed in Section 4.5
Certainty of evidence	22	Present assessments of certainty (or confidence) in the body of evidence for each outcome assessed.	Section 4.5 (informal judgement; confidence in the evidence for most outcomes is limited)
		**DISCUSSION**	
Discussion	23a	Provide a general interpretation of the results in the context of other evidence.	Sections 4.1–4.4
	23b	Discuss any limitations of the evidence included in the review.	Section 4.5
	23c	Discuss any limitations of the review processes used.	Section 4.5
	23d	Discuss implications of the results for practice, policy, and future research.	Sections 4.6, 5 and 6
		**OTHER INFORMATION**	
Registration and protocol	24a	Provide registration information for the review, including register name and registration number, or state that the review was not registered.	Section 2.1 — registered in PROSPERO on 5 August 2026 (CRD420261467473); registration was retrospective
	24b	Indicate where the review protocol can be accessed, or state that a protocol was not prepared.	Section 2.1 — the protocol is available in the PROSPERO record CRD420261467473
	24c	Describe and explain any amendments to information provided at registration or in the protocol.	Not applicable

Section and Topic	Item #	Checklist item	Location where item is reported
Support	25	Describe sources of financial or non-financial support for the review, and the role of the funders or sponsors in the review.	Back matter, Funding statement
Competing interests	26	Declare any competing interests of review authors.	Back matter, Conflicts of Interest statement
Availability of data, code and other materials	27	Report which of the following are publicly available and where they can be found: template data collection forms; data extracted from included studies; data used for all analyses; analytic code; any other materials used in the review.	Back matter, Data Availability Statement; data extracted from the included studies are provided in Supplementary Tables S1–S3

From: Page MJ, McKenzie JE, Bossuyt PM, Boutron I, Hoffmann TC, Mulrow CD, et al. The PRISMA 2020 statement: an updated guideline for reporting systematic reviews. BMJ 2021;372:n71. doi: 10.1136/bmj.n71. This work is licensed under CC BY 4.0. To view a copy of this license, visit https://creativecommons.org/licenses/by/4.0/

Supplementary Table S2. Complete database-specific search strategies in each database used

Database	Complete Database-Specific Search Strategies
PubMed	((("Combat sport"[Title/Abstract] OR "judo"[Title/Abstract] OR "taekwondo"[Title/Abstract] OR "Mixed martial arts"[Title/Abstract] OR "wrestlers"[Title/Abstract] OR "boxing"[Title/Abstract] OR "martial arts"[Title/Abstract] OR "karate"[Title/Abstract]) AND ("nutrition"[Title/Abstract] OR "weight cutting"[Title/Abstract] OR "making weight"[Title/Abstract] OR "rapid weight loss"[Title/Abstract] OR "weight reduction"[Title/Abstract] OR "body composition"[Title/Abstract] OR "body mass reduction"[Title/Abstract] OR "dehydration"[Title/Abstract] OR "rehydration"[Title/Abstract] OR "weight making strategies"[Title/Abstract] OR "weight loss methods"[Title/Abstract] OR "body fat"[Title/Abstract] OR "body fluid"[Title/Abstract] OR "energy restriction"[Title/Abstract] OR "dietary intake"[Title/Abstract] OR "recovery"[Title/Abstract]) AND ("competition"[Title/Abstract] OR "Olympic games"[Title/Abstract] OR "elite"[Title/Abstract] OR "cognition"[Title/Abstract] OR "abilities"[Title/Abstract] OR "physiology"[Title/Abstract] OR "injuries"[Title/Abstract] OR "performance"[Title/Abstract] OR "emotional intelligence"[Title/Abstract]))) AND (2000/01/01:2025/09/30[Date - Publication]) AND (English[Language] OR Spanish[Language] OR Portuguese[Language]) Limits applied: Publication Year: 2000-2025; Languages: English, Spanish, Portuguese. Thematic restriction achieved via Title/Abstract keyword syntax
MEDLINE (via Ovid)	(("Combat sport" OR judo OR taekwondo OR "Mixed martial arts" OR wrestlers OR boxing OR "martial arts" OR karate).ti,ab. AND (nutrition OR "weight cutting" OR "making weight" OR "rapid weight loss" OR "weight reduction" OR "body composition" OR "body mass reduction" OR dehydration OR rehydration OR "weight making strategies" OR "weight loss methods" OR "body fat" OR "body fluid" OR "energy restriction" OR "dietary intake" OR recovery).ti,ab. AND (competition OR "Olympic games" OR elite OR cognition OR abilities OR physiology OR injuries OR performance OR "emotional intelligence").ti,ab.) Limits applied: Publication Year: 2000-2025; Languages: English, Spanish, Portuguese. Thematic restriction achieved via Title/Abstract keyword syntax
Search strategy used in Scopus	TITLE-ABS-KEY(("Combat sport" OR judo OR taekwondo OR "Mixed martial arts" OR wrestlers OR boxing OR "martial arts" OR karate) AND (nutrition OR "weight cutting" OR "making weight" OR "rapid weight loss" OR "weight reduction" OR "body composition" OR "body mass reduction" OR dehydration OR rehydration OR "weight making strategies" OR "weight loss methods" OR "body fat" OR "body fluid" OR "energy restriction" OR "dietary intake" OR recovery) AND (competition OR "Olympic games" OR elite OR cognition OR abilities OR physiology OR injuries OR performance OR "emotional intelligence")) AND PUBYEAR > 1999 AND PUBYEAR < 2026 AND (LIMIT-TO(LANGUAGE, "English") OR LIMIT-TO(LANGUAGE, "Spanish") OR LIMIT-TO(LANGUAGE, "Portuguese")) AND (LIMIT-TO(SUBJAREA, "MEDI") OR LIMIT-TO(SUBJAREA, "HEAL") OR LIMIT-TO(SUBJAREA, "NURS")) Limits applied: Publication Year: 2000-2025; Languages:

	English, Spanish, Portuguese; Subject Areas restricted to Medicine (MEDI), Health Professions (HEAL), and Nursing (NURS)
Search strategy used in SPORTDiscus (via EBSCOhost)	(TI("Combat sport" OR judo OR taekwondo OR "Mixed martial arts" OR wrestlers OR boxing OR "martial arts" OR karate) OR AB("Combat sport" OR judo OR taekwondo OR "Mixed martial arts" OR wrestlers OR boxing OR "martial arts" OR karate)) AND (TI(nutrition OR "weight cutting" OR "making weight" OR "rapid weight loss" OR "weight reduction" OR "body composition" OR "body mass reduction" OR dehydration OR rehydration OR "weight making strategies" OR "weight loss methods" OR "body fat" OR "body fluid" OR "energy restriction" OR "dietary intake" OR recovery) OR AB(nutrition OR "weight cutting" OR "making weight" OR "rapid weight loss" OR "weight reduction" OR "body composition" OR "body mass reduction" OR dehydration OR rehydration OR "weight making strategies" OR "weight loss methods" OR "body fat" OR "body fluid" OR "energy restriction" OR "dietary intake" OR recovery)) AND (TI(competition OR "Olympic games" OR elite OR cognition OR abilities OR physiology OR injuries OR performance OR "emotional intelligence") OR AB(competition OR "Olympic games" OR elite OR cognition OR abilities OR physiology OR injuries OR performance OR "emotional intelligence")) Limits applied: Published Date: 20000101-20250930; Language: English, Spanish, Portuguese; Peer Reviewed: True. Database content inherently restricted to sports and physical education disciplines
Search strategy used in SciELO	((ti:("Combat sport" OR judo OR taekwondo OR "Mixed martial arts" OR wrestlers OR boxing OR "martial arts" OR karate)) OR (ab:("Combat sport" OR judo OR taekwondo OR "Mixed martial arts" OR wrestlers OR boxing OR "martial arts" OR karate))) AND ((ti:("nutrition" OR "weight cutting" OR "making weight" OR "rapid weight loss" OR "weight reduction" OR "body composition" OR "body mass reduction" OR "dehydration" OR "rehydration" OR "weight making strategies" OR "weight loss methods" OR "body fat" OR "body fluid" OR "energy restriction" OR "dietary intake" OR "recovery")) OR (ab:("nutrition" OR "weight cutting" OR "making weight" OR "rapid weight loss" OR "weight reduction" OR "body composition" OR "body mass reduction" OR "dehydration" OR "rehydration" OR "weight making strategies" OR "weight loss methods" OR "body fat" OR "body fluid" OR "energy restriction" OR "dietary intake" OR "recovery"))) AND ((ti:("competition" OR "Olympic games" OR "elite" OR "cognition" OR "abilities" OR "physiology" OR "injuries" OR "performance" OR "emotional intelligence")) OR (ab:("competition" OR "Olympic games" OR "elite" OR "cognition" OR "abilities" OR "physiology" OR "injuries" OR "performance" OR "emotional intelligence"))) Limits applied: year_cluster:("2000" TO "2025"); la:("en" OR "es" OR "pt"); Subject Areas restricted to Health Sciences and Biological Sciences

Supplementary Table S3. Outcome-level results of all eligible outcomes reported in the included studies, significant or not

One row per outcome and per group or experimental condition, as named in each original article. Test names are kept as in the source (no harmonisation across studies). Rows are ordered by reference number; shading alternates between studies.

Conventions:

- "not reported" (NR): the item does not appear in the article. No value was estimated from graphs; "Figure only" means the data are shown exclusively in a figure.
- Pre/Post values are mean ± SD unless stated otherwise (SE, median [Q1; Q3], 95% CI). † = SD derived from SE × √n, applied only where the article states the exact n.
- For crossover designs without a pre-intervention test, the weight-loss condition row carries the between-condition statistics; the control/reference condition row is marked "n/a (reference)".
- Statistically significant: "Yes/No" refers to the comparison stated in brackets. When a main effect or interaction was not significant but a post hoc comparison was, both are shown (e.g., "No (main effect) / Yes (post hoc P1 vs P2)"), and the specific comparison is given in the statistic column.
- Direction: effect on the athlete's performance or psychological state for the row's significant comparison (Favourable = improvement; Unfavourable = impairment; Null = no significant difference; Unclear = significant change in a physiological or contractile marker without an evident performance direction, or conflicting information in the source).
- Values in square brackets within p values (Isacco et al. [39]) are digits displaced in the PDF text layer; minus signs rendered as other characters were reconstructed where flagged (Brandt et al. [32]; Koral & Dosseville [33]).
- Studies used different significance thresholds: Zubac et al. [43] used p < 0.10 for interaction effects; Rossi et al. [45] and Ceylan et al. [44] treated p ≤ 0.05 as significant; Barley et al. [4] report 90% CIs.
- Variables not considered eligible performance/psychological outcomes (body mass and composition, hydration markers, blood biochemistry, dietary intake, disordered-eating subscales) were not extracted.

Study (first author, year) [ref]	Group / condition	Outcome	Time point	Pre value (mean ± SD)	Post value (mean ± SD)	Change (absolute and/or %)	Test statistic and p value	Effect size (95% CI if reported)	Statistically significant (Yes / No)	Direction (Favourable / Unfavourable / Null)	Notes
Barley et al., 2018 [4]	DHY (dehydration: 3 h cycling at 60 W, ~40 °C, sweat suit, no fluids; n = 14)	Repeat sled push test (75% BM) – mean effort time (s)	3 h post-intervention	not reported	6.68 ± 1.48	not reported	Two-way RM ANOVA + Holm–Bonferroni post hoc; DHY vs CONT p = 0.001; 90% CI of difference (0.88, 2.02)	Hedges g = 1.229	Yes	Unfavourable	Crossover: no pre-intervention performance test (familiarisation only); comparison is DHY vs CONT at the same time point. CIs are 90%, not 95%. Abstract: sled push times 19 ± 15% slower.
Barley et al., 2018 [4]	CONT (thermoneutral control, 25 °C, fluids allowed; n = 14)	Repeat sled push test (75% BM) – mean effort time (s)	3 h post-intervention	not reported	5.23 ± 0.66	not reported	Reference condition (comparison reported in DHY row)	see DHY row	n/a (reference)	n/a (reference)	—
Barley et al., 2018 [4]	DHY (dehydration: 3 h cycling at 60 W, ~40 °C, sweat suit, no fluids; n = 14)	Repeat sled push test (75% BM) – mean effort time (s)	24 h post-intervention	not reported	5.94 ± 1.42	not reported	DHY vs CONT p = 0.012; 90% CI (0.42, 1.18)	Hedges g = 0.671	Yes	Unfavourable	Crossover: no pre-intervention performance test (familiarisation only); comparison is DHY vs CONT at the same time point. CIs are 90%, not 95%. Abstract: 14 ± 15% slower.
Barley et al., 2018 [4]	CONT (thermoneutral control, 25 °C, fluids allowed; n = 14)	Repeat sled push test (75% BM) – mean effort time (s)	24 h post-intervention	not reported	5.15 ± 0.77	not reported	Reference condition (comparison reported in DHY row)	see DHY row	n/a (reference)	n/a (reference)	—

Study (first author, year) [ref]	Group / condition	Outcome	Time point	Pre value (mean ± SD)	Post value (mean ± SD)	Change (absolute and/or %)	Test statistic and p value	Effect size (95% CI if reported)	Statistically significant (Yes / No)	Direction (Favourable / Unfavourable / Null)	Notes
Barley et al., 2018 [4]	DHY (dehydration: 3 h cycling at 60 W, ~40 °C, sweat suit, no fluids; n = 14)	Repeat sled push test – peak effort time (s)	3 h post	not reported	4.22 ± 0.58	not reported	p = 0.1; 90% CI (0.06, 0.36)	Hedges g = 0.362	No	Null	Crossover: no pre-intervention performance test (familiarisation only); comparison is DHY vs CONT at the same time point. CIs are 90%, not 95%.
Barley et al., 2018 [4]	CONT (thermoneutral control, 25 °C, fluids allowed; n = 14)	Repeat sled push test – peak effort time (s)	3 h post	not reported	4 ± 0.6	not reported	Reference condition (comparison reported in DHY row)	see DHY row	n/a (reference)	n/a (reference)	—
Barley et al., 2018 [4]	DHY (dehydration: 3 h cycling at 60 W, ~40 °C, sweat suit, no fluids; n = 14)	Repeat sled push test – peak effort time (s)	24 h post	not reported	4.18 ± 0.73	not reported	p = 0.042; 90% CI (0.13, 0.44)	Hedges g = 0.434	Yes	Unfavourable	Crossover: no pre-intervention performance test (familiarisation only); comparison is DHY vs CONT at the same time point. CIs are 90%, not 95%.
Barley et al., 2018 [4]	CONT (thermoneutral control, 25 °C, fluids allowed; n = 14)	Repeat sled push test – peak effort time (s)	24 h post	not reported	3.9 ± 0.5	not reported	Reference condition (comparison reported in DHY row)	see DHY row	n/a (reference)	n/a (reference)	—
Barley et al., 2018 [4]	DHY (dehydration: 3 h cycling at 60 W, ~40 °C, sweat suit, no fluids; n = 14)	Repeat sled push test – fatigue index (s)	3 h post	not reported	3 ± 2.21	not reported	p = 0.075; 90% CI (0.8, 3.01)	Hedges g = 1.112	No	Null	Crossover: no pre-intervention performance test (familiarisation only); comparison is DHY vs CONT at the same time point. CIs are 90%, not 95%.

Study (first author, year) [ref]	Group / condition	Outcome	Time point	Pre value (mean ± SD)	Post value (mean ± SD)	Change (absolute and/or %)	Test statistic and p value	Effect size (95% CI if reported)	Statistically significant (Yes / No)	Direction (Favourable / Unfavourable / Null)	Notes
Barley et al., 2018 [4]	CONT (thermoneutral control, 25 °C, fluids allowed; n = 14)	Repeat sled push test – fatigue index (s)	3 h post	not reported	1.1 ± 0.79	not reported	Reference condition (comparison reported in DHY row)	see DHY row	n/a (reference)	n/a (reference)	—
Barley et al., 2018 [4]	DHY (dehydration: 3 h cycling at 60 W, ~40 °C, sweat suit, no fluids; n = 14)	Repeat sled push test – fatigue index (s)	24 h post	not reported	1.4 ± 1.06	not reported	p = 0.426; 90% CI (0.09, 1.03)	Hedges g = 0.681	No	Null	Crossover: no pre-intervention performance test (familiarisation only); comparison is DHY vs CONT at the same time point. CIs are 90%, not 95%.
Barley et al., 2018 [4]	CONT (thermoneutral control, 25 °C, fluids allowed; n = 14)	Repeat sled push test – fatigue index (s)	24 h post	not reported	0.84 ± 0.39	not reported	Reference condition (comparison reported in DHY row)	see DHY row	n/a (reference)	n/a (reference)	—
Barley et al., 2018 [4]	DHY (dehydration: 3 h cycling at 60 W, ~40 °C, sweat suit, no fluids; n = 14)	Repeat sled push test – total test time (s)	3 h post	not reported	811 ± 62	not reported	p = 0.028; 90% CI (27, 77)	Hedges g = 1.068	Yes	Unfavourable	Crossover: no pre-intervention performance test (familiarisation only); comparison is DHY vs CONT at the same time point. CIs are 90%, not 95%.
Barley et al., 2018 [4]	CONT (thermoneutral control, 25 °C, fluids allowed; n = 14)	Repeat sled push test – total test time (s)	3 h post	not reported	759 ± 25	not reported	Reference condition (comparison reported in DHY row)	see DHY row	n/a (reference)	n/a (reference)	—

Study (first author, year) [ref]	Group / condition	Outcome	Time point	Pre value (mean ± SD)	Post value (mean ± SD)	Change (absolute and/or %)	Test statistic and p value	Effect size (95% CI if reported)	Statistically significant (Yes / No)	Direction (Favourable / Unfavourable / Null)	Notes
Barley et al., 2018 [4]	DHY (dehydration: 3 h cycling at 60 W, ~40 °C, sweat suit, no fluids; n = 14)	Repeat sled push test – total test time (s)	24 h post	not reported	788 ± 52	not reported	p = 0.046; 90% CI (12, 42)	Hedges g = 0.640	Yes	Unfavourable	Crossover: no pre-intervention performance test (familiarisation only); comparison is DHY vs CONT at the same time point. CIs are 90%, not 95%. DHY 24 h also differed from DHY 3 h (* p < 0.05).
Barley et al., 2018 [4]	CONT (thermoneutral control, 25 °C, fluids allowed; n = 14)	Repeat sled push test – total test time (s)	24 h post	not reported	760 ± 30	not reported	Reference condition (comparison reported in DHY row)	see DHY row	n/a (reference)	n/a (reference)	—
Barley et al., 2018 [4]	DHY (dehydration: 3 h cycling at 60 W, ~40 °C, sweat suit, no fluids; n = 14)	Repeat sled push test – runs completed (n)	3 h post	not reported	26.64 ± 5.7	not reported	p = 0.188; 90% CI (−4.57, −0.57)	Hedges g = 0.550	No	Null	Crossover: no pre-intervention performance test (familiarisation only); comparison is DHY vs CONT at the same time point. CIs are 90%, not 95%. Text: 10 vs 13 athletes completed the test at 3 h (DHY vs CONT); no test reported for completion counts. Athletes not completing were excluded from time analyses.
Barley et al., 2018 [4]	CONT (thermoneutral control, 25 °C, fluids allowed; n = 14)	Repeat sled push test – runs completed (n)	3 h post	not reported	29.21 ± 2.94	not reported	Reference condition (comparison reported in DHY row)	see DHY row	n/a (reference)	n/a (reference)	—

Study (first author, year) [ref]	Group / condition	Outcome	Time point	Pre value (mean ± SD)	Post value (mean ± SD)	Change (absolute and/or %)	Test statistic and p value	Effect size (95% CI if reported)	Statistically significant (Yes / No)	Direction (Favourable / Unfavourable / Null)	Notes
Barley et al., 2018 [4]	DHY (dehydration: 3 h cycling at 60 W, ~40 °C, sweat suit, no fluids; n = 14)	Repeat sled push test – runs completed (n)	24 h post	not reported	29.21 ± 2.94	not reported	p = 0.336; 90% CI (−0.19, 0.05)	Hedges g = 0.028	No	Null	Crossover: no pre-intervention performance test (familiarisation only); comparison is DHY vs CONT at the same time point. CIs are 90%, not 95%. CONT dispersion printed as '+ 2.68' (typographical). DHY 24 h value identical to CONT 3 h value (29.21 ± 2.94) – possible transcription error in the original.
Barley et al., 2018 [4]	CONT (thermoneutral control, 25 °C, fluids allowed; n = 14)	Repeat sled push test – runs completed (n)	24 h post	not reported	29.29 + 2.68	not reported	Reference condition (comparison reported in DHY row)	see DHY row	n/a (reference)	n/a (reference)	—
Barley et al., 2018 [4]	DHY (dehydration: 3 h cycling at 60 W, ~40 °C, sweat suit, no fluids; n = 14)	Vertical jump (cm)	3 h post	not reported	47.71 ± 7.84	not reported	p = N/A (no main effect); 90% CI (−2.46, −0.36)	Hedges g = 0.171	No	Null	Crossover: no pre-intervention performance test (familiarisation only); comparison is DHY vs CONT at the same time point. CIs are 90%, not 95%. Abstract reports p = 0.467 for vertical jump (time point not specified).
Barley et al., 2018 [4]	CONT (thermoneutral control, 25 °C, fluids allowed; n = 14)	Vertical jump (cm)	3 h post	not reported	49.14 ± 8.4	not reported	Reference condition (comparison reported in DHY row)	see DHY row	n/a (reference)	n/a (reference)	—

Study (first author, year) [ref]	Group / condition	Outcome	Time point	Pre value (mean ± SD)	Post value (mean ± SD)	Change (absolute and/or %)	Test statistic and p value	Effect size (95% CI if reported)	Statistically significant (Yes / No)	Direction (Favourable / Unfavourable / Null)	Notes
Barley et al., 2018 [4]	DHY (dehydration: 3 h cycling at 60 W, ~40 °C, sweat suit, no fluids; n = 14)	Vertical jump (cm)	24 h post	not reported	47.5 ± 9.25	not reported	p = N/A (no main effect); 90% CI (−2.46, 0.46)	Hedges g = 0.115	No	Null	Crossover: no pre-intervention performance test (familiarisation only); comparison is DHY vs CONT at the same time point. CIs are 90%, not 95%.
Barley et al., 2018 [4]	CONT (thermoneutral control, 25 °C, fluids allowed; n = 14)	Vertical jump (cm)	24 h post	not reported	48.5 ± 7.49	not reported	Reference condition (comparison reported in DHY row)	see DHY row	n/a (reference)	n/a (reference)	—
Barley et al., 2018 [4]	DHY (dehydration: 3 h cycling at 60 W, ~40 °C, sweat suit, no fluids; n = 14)	Hand grip (kg)	3 h post	not reported	51 ± 8	not reported	p = 0.044; 90% CI (−4.05, −0.66) (Table 2) / (−4.04, −0.66) (text)	Hedges g = 0.243	Yes	Unfavourable	Crossover: no pre-intervention performance test (familiarisation only); comparison is DHY vs CONT at the same time point. CIs are 90%, not 95%. Minor text/table discrepancy in lower CI limit. Hand not specified.
Barley et al., 2018 [4]	CONT (thermoneutral control, 25 °C, fluids allowed; n = 14)	Hand grip (kg)	3 h post	not reported	53 ± 8	not reported	Reference condition (comparison reported in DHY row)	see DHY row	n/a (reference)	n/a (reference)	—

Study (first author, year) [ref]	Group / condition	Outcome	Time point	Pre value (mean ± SD)	Post value (mean ± SD)	Change (absolute and/or %)	Test statistic and p value	Effect size (95% CI if reported)	Statistically significant (Yes / No)	Direction (Favourable / Unfavourable / Null)	Notes
Barley et al., 2018 [4]	DHY (dehydration: 3 h cycling at 60 W, ~40 °C, sweat suit, no fluids; n = 14)	Hand grip (kg)	24 h post	not reported	54 ± 9	not reported	p = 0.102; 90% CI (−2.03, −0.12)	Hedges g = 0.105	No	Null	Crossover: no pre-intervention performance test (familiarisation only); comparison is DHY vs CONT at the same time point. CIs are 90%, not 95%.
Barley et al., 2018 [4]	CONT (thermoneutral control, 25 °C, fluids allowed; n = 14)	Hand grip (kg)	24 h post	not reported	56 ± 10	not reported	Reference condition (comparison reported in DHY row)	see DHY row	n/a (reference)	n/a (reference)	—
Barley et al., 2018 [4]	DHY (dehydration: 3 h cycling at 60 W, ~40 °C, sweat suit, no fluids; n = 14)	Medicine ball chest throw (cm)	3 h post	not reported	454 ± 46	not reported	p = 0.276; 90% CI (−0.23, −0.01)	Hedges g = 0.245	No	Null	Crossover: no pre-intervention performance test (familiarisation only); comparison is DHY vs CONT at the same time point. CIs are 90%, not 95%. Units of the CI unclear (values incompatible with a difference in cm).
Barley et al., 2018 [4]	CONT (thermoneutral control, 25 °C, fluids allowed; n = 14)	Medicine ball chest throw (cm)	3 h post	not reported	466 ± 49	not reported	Reference condition (comparison reported in DHY row)	see DHY row	n/a (reference)	n/a (reference)	—

Study (first author, year) [ref]	Group / condition	Outcome	Time point	Pre value (mean ± SD)	Post value (mean ± SD)	Change (absolute and/or %)	Test statistic and p value	Effect size (95% CI if reported)	Statistically significant (Yes / No)	Direction (Favourable / Unfavourable / Null)	Notes
Barley et al., 2018 [4]	DHY (dehydration: 3 h cycling at 60 W, ~40 °C, sweat suit, no fluids; n = 14)	Medicine ball chest throw (cm)	24 h post	not reported	449 ± 44	not reported	p = 0.016; 90% CI (−0.36, −0.13)	Hedges g = 0.253	Yes	Unfavourable	Crossover: no pre-intervention performance test (familiarisation only); comparison is DHY vs CONT at the same time point. CIs are 90%, not 95%. Units of the CI unclear (values incompatible with a 25-cm difference).
Barley et al., 2018 [4]	CONT (thermoneutral control, 25 °C, fluids allowed; n = 14)	Medicine ball chest throw (cm)	24 h post	not reported	474 ± 52	not reported	Reference condition (comparison reported in DHY row)	see DHY row	n/a (reference)	n/a (reference)	—
Barley et al., 2018 [4]	DHY (n = 14)	RPE during repeat sled push (Borg 6–20)	3 h and 24 h post	not reported	Figure only	not reported	* p < 0.05 vs CONT at several effort blocks (Figure 1C); exact p NR	not reported	Yes	Unfavourable	Data shown only in Figure 1C (not extracted from graph).
Barley et al., 2018 [4]	DHY (n = 14)	Heart rate during repeat sled push (bpm)	3 h and 24 h post	not reported	Figure only	not reported	No significant differences vs CONT (text); exact p NR	not reported	No	Null	Data shown only in Figure 1B.
Barley et al., 2018 [4]	DHY (n = 14)	Sled push speed by effort block (km·h^{-1})	3 h and 24 h post	not reported	Figure only	not reported	* p < 0.05 vs CONT at several effort blocks (Figure 1A); exact p NR	not reported	Yes	Unfavourable	Data shown only in Figure 1A.
de Sousa Fortes et al., 2017 [27]	EG (experimental group; n = 20)	GPAI (decision-making index) in simulated judo fight	48 h before vs 48 h after 2-wk period	0.48 ± 0.1	0.49 ± 0.1	Δ% 1.2	Two-way RM ANOVA: group F = 36.04, p = 0.01; time F = 31.73, p = 0.23; interaction F = 40.59, p = 0.01 (text: p < 0.01). Within-EG: no significant change (p NR)	d = 0.1	No (within-EG); interaction Yes	Null	Reported F and p values appear mutually inconsistent (e.g., F = 31.73, p = 0.23).
de Sousa Fortes et al., 2017 [27]	CG (control group; n = 19)	GPAI (decision-making index) in simulated judo fight	48 h before vs 48 h after	0.47 ± 0.1	0.52 ± 0.1	Δ% 5.1	Interaction p < 0.01; post hoc within-CG pre–post p = 0.01	d = 0.7	Yes (post hoc, within-CG)	Favourable	Pre-intervention GPAI did not differ between groups (p = 0.26).

Study (first author, year) [ref]	Group / condition	Outcome	Time point	Pre value (mean ± SD)	Post value (mean ± SD)	Change (absolute and/or %)	Test statistic and p value	Effect size (95% CI if reported)	Statistically significant (Yes / No)	Direction (Favourable / Unfavourable / Null)	Notes
Fortes et al., 2017 [28]	EG (experimental group; n = 20)	Special Judo Fitness Test (index)	24 h before vs 24 h after 2-wk period	13.3 ± 1.4	13.2 ± 1.22	Δ% 0.7	Group F = 30.64, p = 0.01; time F = 27.51, p = 0.23; interaction F = 37.85, p = 0.01 (text: p < 0.01). Within-EG: no significant change (p NR)	d = 0.1	No (within-EG); interaction Yes	Null	Lower index = better performance. Article states Δ% 0.7 for a decrease 13.3→13.2 (sign not given). EG not allowed ad libitum refeeding/rehydration before post-test.
Fortes et al., 2017 [28]	CG (control group; n = 19)	Special Judo Fitness Test (index)	24 h before vs 24 h after	13.1 ± 1.2	12.4 ± 1.25	Δ% −6.0	Interaction p = 0.01; post hoc within-CG p = 0.01	d = 0.7 (small)	Yes (post hoc, within-CG)	Favourable	—
Fortes et al., 2017 [28]	EG (n = 20)	Number of throws in the SJFT (total)	24 h before vs 24 h after	20.3 ± 2.1	19.1 ± 2.2	Δ% −5.9	Group F = 41.50, p = 0.01; time F = 35.28, p = 0.32; interaction F = 46.33, p = 0.01 (table) / p = 0.001 (text); post hoc within-EG p = 0.01	d = 0.7 (small)	Yes (post hoc, within-EG)	Unfavourable	Discrepancy between text (interaction p = 0.001) and Table 2 (p = 0.01).
Fortes et al., 2017 [28]	CG (n = 19)	Number of throws in the SJFT (total)	24 h before vs 24 h after	20 ± 2.3	21 ± 2.4	Δ% 4.3	Interaction as above; post hoc within-CG p = 0.01	d = 0.7 (small)	Yes (post hoc, within-CG)	Favourable	—
Fortes et al., 2017 [28]	EG (n = 20)	HR after SJFT (bpm)	24 h before vs 24 h after	180.5 ± 4.4	181.2 ± 4.3	Δ% 0.5	Group F = 2.06, p = 0.23; time F = 1.24, p = 0.29; interaction F = 2.29, p = 0.18	d = 0.1	No	Null	SJFT component (used to compute index).
Fortes et al., 2017 [28]	CG (n = 19)	HR after SJFT (bpm)	24 h before vs 24 h after	180.7 ± 4.1	181.6 ± 5.2	Δ% 0.5	As EG row (no group, time or interaction effect)	d = 0.1	No	Null	—
Fortes et al., 2017 [28]	EG (n = 20)	HR 1 min after SJFT (bpm)	24 h before vs 24 h after	135.8 ± 5.0	144.3 ± 4.2	Δ% 6.3	Group F = 50.61, p = 0.01; time F = 42.97, p = 0.22; interaction F = 57.44, p = 0.01 (table) / p = 0.001 (text); post hoc within-EG p = 0.01	d = 0.7 (small)	Yes (post hoc, within-EG)	Unfavourable	Higher HR 1 min = poorer recovery. Text/table discrepancy for interaction p.
Fortes et al., 2017 [28]	CG (n = 19)	HR 1 min after SJFT (bpm)	24 h before vs 24 h after	135.4 ± 4.7	132.6 ± 4.8	Δ% −2.2	Post hoc within-CG p = 0.01	d = 0.5 (small)	Yes (post hoc, within-CG)	Favourable	—

Study (first author, year) [ref]	Group / condition	Outcome	Time point	Pre value (mean ± SD)	Post value (mean ± SD)	Change (absolute and/or %)	Test statistic and p value	Effect size (95% CI if reported)	Statistically significant (Yes / No)	Direction (Favourable / Unfavourable / Null)	Notes
Fortes et al., 2019 [29]	EG (experimental group; n = 20)	DALDA Part B (symptoms of stress; score)	48 h before vs 48 h after 2-wk period	34.23 ± 1.63 SE [SD 7.29†]	39.51 ± 1.86 SE [SD 8.32†]	not reported	Repeated-measures MANOVA: time F(2,37) = 36.14, p = 0.001; group F(2,37) = 49.56, p = 0.001 (interaction NR). Within-EG pre–post F(2,18) = 43.63, p = 0.01; EG vs CG at post F(2,37) = 38.55, p = 0.01	d = 0.6 (EG vs CG at post)	Yes (within-EG and between groups at post)	Unfavourable	†SD derived from SE × √n (n = 20 stated exactly); article Table 3 reports mean ± SE. Discrepancy: Table 2 (pre-test descriptives, mean ± SD) gives EG DALDA 36.25 ± 7.49, whereas Table 3 gives 34.23 at pre-test. Higher score = more stress symptoms. Group × time interaction not reported. The analysed PDF is the 2017 online-first version (DOI 10.1080/1612197X.2017.1303530).
Fortes et al., 2019 [29]	CG (control group; n = 19)	DALDA Part B (symptoms of stress; score)	48 h before vs 48 h after	34.65 ± 1.74 SE [SD 7.58†]	34.19 ± 1.82 SE [SD 7.93†]	not reported	Within-CG F(2,17) = 3.14, p = 0.18	not reported	No	Null	†SD derived from SE × √n (n = 19). Table 2 pre-test value: 36.91 ± 7.12 (SD), inconsistent with Table 3 (34.65).
Fortes et al., 2019 [29]	EG (n = 20)	Recovery (RESTQ-Sport; sum of recovery subscales, ΣR)	48 h before vs 48 h after	101.40 ± 2.52 SE [SD 11.27†]	87.63 ± 2.47 SE [SD 11.05†]	not reported	Within-EG F(2,18) = 69.97, p = 0.001; EG vs CG at post F(2,37) = 116.75, p = 0.001	d = 0.9 (EG vs CG at post)	Yes (within-EG and between groups at post)	Unfavourable	†SD derived from SE × √n (n = 20). Table 2 pre-test value: 104.40 ± 12.84 (SD), inconsistent with Table 3 (101.40). Body weight showed 'collinearity' with recovery (F(2,37) = 14.86, p = 0.03).
Fortes et al., 2019 [29]	CG (n = 19)	Recovery (RESTQ-Sport; sum of recovery subscales, ΣR)	48 h before vs 48 h after	100.97 ± 2.80 SE [SD 12.21†]	109.30 ± 2.71 SE [SD 11.81†]	not reported	Within-CG F(2,18) = 47.61, p = 0.01	not reported	Yes (within-CG)	Favourable	†SD derived from SE × √n (n = 19). Table 2 pre-test: 102.89 ± 13.09 (SD). Degrees of freedom F(2,18) for n = 19 as printed.

Study (first author, year) [ref]	Group / condition	Outcome	Time point	Pre value (mean ± SD)	Post value (mean ± SD)	Change (absolute and/or %)	Test statistic and p value	Effect size (95% CI if reported)	Statistically significant (Yes / No)	Direction (Favourable / Unfavourable / Null)	Notes
De Sousa Fortes et al., 2017 [30]	EG (weight loss group; n = 15)	SEI (index), whole 3-round match (GPAI)	Pre (24 h before) vs post (24 h after 2-wk period)	0.42 ± 0.1 (95% CI 0.39–0.44)	0.43 ± 0.1 (95% CI 0.37–0.45)	Δ% 1.3 ± 0.4 (95% CI 0.9–2.3)	Two-way RM ANOVA: group F = 39.22, p = 0.001; time F = 32.54, p = 0.190; interaction F = 47.70, p = 0.001. Within-EG pre–post: no significant change (p NR)	d = 0.1 (trivial)	No (within-EG); interaction Yes	Null	Interaction significant, driven by improvement in CG only. SD reported to one decimal (0.1) for all SEI values. Reported F values appear inconsistent with the p values (e.g., time F = 32.54 with p = 0.190).
De Sousa Fortes et al., 2017 [30]	CG (control group; n = 16)	SEI (index), whole 3-round match (GPAI)	Pre vs post	0.41 ± 0.1 (95% CI 0.38–0.46)	0.49 ± 0.1 (95% CI 0.45–0.53)	Δ% 8.4 ± 1.6 (95% CI 6.8–9.2)	Interaction p < 0.001 (text) / 0.001 (table); post hoc within-CG pre–post p = 0.01	d = 1.1 (moderate; 95% CI 0.7–1.4)	Yes (post hoc, within-CG)	Favourable	—
De Sousa Fortes et al., 2017 [30]	EG (n = 15)	SEI (index), round 1	Pre and post (round-level analysis, Table 3)	0.39 ± 0.2 (95% CI 0.37–0.48)	0.45 ± 0.1 (95% CI 0.36–0.48)	not reported	Round-level ANOVA at post: group F = 36.23, p = 0.01; time F = 38.05, p = 0.03; interaction F = 41.98, p = 0.02 (pre: group F = 3.39, p = 0.31; time F = 4.01, p = 0.33; interaction F = 4.76, p = 0.46). No post hoc difference flagged for round 1	not reported	No	Null	—
De Sousa Fortes et al., 2017 [30]	CG (n = 16)	SEI (index), round 1	Pre and post (round-level analysis, Table 3)	0.41 ± 0.1 (95% CI 0.36–0.43)	0.44 ± 0.1 (95% CI 0.40–0.49)	not reported	See EG round 1 row (same ANOVA). No post hoc difference flagged for round 1	not reported	No	Null	—
De Sousa Fortes et al., 2017 [30]	EG (n = 15)	SEI (index), round 2	Pre and post (Table 3)	0.42 ± 0.2 (95% CI 0.36–0.46)	0.40 ± 0.2 (95% CI 0.32–0.45)	not reported	Post: EG round 2 < EG round 1 (# p < 0.05 vs first round); text: p = 0.03	d = 0.6 (small; 95% CI 0.4–1.2)	Yes (post hoc, round 2 vs round 1 at post)	Unfavourable	Post hoc comparison is round 2 vs round 1 within EG at post-test, not pre vs post.

Study (first author, year) [ref]	Group / condition	Outcome	Time point	Pre value (mean ± SD)	Post value (mean ± SD)	Change (absolute and/or %)	Test statistic and p value	Effect size (95% CI if reported)	Statistically significant (Yes / No)	Direction (Favourable / Unfavourable / Null)	Notes
De Sousa Fortes et al., 2017 [30]	CG (n = 16)	SEI (index), round 2	Pre and post (Table 3)	0.38 ± 0.2 (95% CI 0.33–0.44)	0.51 ± 0.2 (95% CI 0.42–0.55)	not reported	Post: CG > EG (* p < 0.05 vs EG); text: 'improvement for the CG in the second round' p = 0.02	d = 0.8 (moderate; 95% CI 0.4–1.0)	Yes (post hoc, CG vs EG at post)	Favourable	Ambiguity: table footnote defines * as 'vs EG' (between-group at post), whereas the text describes it as an 'improvement for the CG'.
De Sousa Fortes et al., 2017 [30]	EG (n = 15)	SEI (index), round 3	Pre and post (Table 3)	0.41 ± 0.1 (95% CI 0.34–0.45)	0.42 ± 0.1 (95% CI 0.35–0.48)	not reported	No post hoc difference flagged for EG round 3	not reported	No	Null	—
De Sousa Fortes et al., 2017 [30]	CG (n = 16)	SEI (index), round 3	Pre and post (Table 3)	0.42 ± 0.1 (95% CI 0.40–0.49)	0.48 ± 0.2 (95% CI 0.44–0.58)	not reported	Post: CG > EG (* p < 0.05 vs EG); text p = 0.01	d = 1.0 (moderate; 95% CI 0.7–1.5)	Yes (post hoc, CG vs EG at post)	Favourable	Same ambiguity as round 2 (between-group vs within-group).
De Sousa Fortes et al., 2017 [30]	EG (n = 15)	Total actions (n) during match	Pre and post (text only)	52 ± 7 (95% CI 47–54)	50 ± 8 (95% CI 47–56)	not reported	No statistical test reported	not reported	not tested	Null	Descriptive only.
De Sousa Fortes et al., 2017 [30]	CG (n = 16)	Total actions (n) during match	Pre and post (text only)	53 ± 8 (95% CI 49–58)	51 ± 7 (95% CI 48–55)	not reported	No statistical test reported	not reported	not tested	Null	Descriptive only.
De Sousa Fortes et al., 2017 [30]	EG (n = 15)	Appropriate actions (n)	Pre and post (text only)	24 ± 5 (95% CI 20–28)	26 ± 4 (95% CI 22–29)	not reported	No statistical test reported	not reported	not tested	Null	Internal inconsistency: appropriate/total actions (26/50 = 0.52 post in EG; 22/51 = 0.43 post in CG) do not reproduce the reported SEI values (EG 0.43; CG 0.49).
De Sousa Fortes et al., 2017 [30]	CG (n = 16)	Appropriate actions (n)	Pre and post (text only)	23 ± 5 (95% CI 20–29)	22 ± 5 (95% CI 19–28)	not reported	No statistical test reported	not reported	not tested	Null	See EG row: inconsistent with SEI.

Study (first author, year) [ref]	Group / condition	Outcome	Time point	Pre value (mean ± SD)	Post value (mean ± SD)	Change (absolute and/or %)	Test statistic and p value	Effect size (95% CI if reported)	Statistically significant (Yes / No)	Direction (Favourable / Unfavourable / Null)	Notes
Ayar et al., 2023 [31]	Experimental group (dietitian-supervised programme, 15–20% energy restriction; n = 8)	POMS – Tension–Anxiety	First vs last day of a 23-day pre-competition training camp	12.38 ± 3.2	10.75 ± 3.85	not reported	Wilcoxon within-EG p = 0.074; Mann–Whitney on change scores EG vs CG p = 0.001	Cohen's d = 2.411 (between-group difference in change)	No (within-EG); Yes (between-group change)	Null	Non-randomised allocation (national-team starters vs substitutes); single-blinded. Wilcoxon signed-rank (within group) and Mann–Whitney U on pre–post differences (between groups); Cohen's d for the between-group comparison of changes. BOTH groups lost weight: EG 84.9 → 81.9 kg (p = 0.012; ≈3.5%), CG 84.9 → 83.5 kg (p = 0.080; ≈1.6%). POMS Turkish 58-item version, raw scores.
Ayar et al., 2023 [31]	Control group (self-selected 'traditional' weight loss methods; n = 8)	POMS – Tension–Anxiety	First vs last day of a 23-day pre-competition training camp	11.13 ± 5.89	17.88 ± 4.79	not reported	Wilcoxon within-CG p = 0.012; between-group p = 0.001 (see EG row)	see EG row	Yes (within-CG)	Unfavourable	Control athletes also cut weight using traditional methods (sauna, excess exercise, food/fluid restriction). Mean ± SD with min–max (median) reported in the original.

Study (first author, year) [ref]	Group / condition	Outcome	Time point	Pre value (mean ± SD)	Post value (mean ± SD)	Change (absolute and/or %)	Test statistic and p value	Effect size (95% CI if reported)	Statistically significant (Yes / No)	Direction (Favourable / Unfavourable / Null)	Notes
Ayar et al., 2023 [31]	Experimental group (dietitian-supervised programme, 15–20% energy restriction; n = 8)	POMS – Depression–Dejection	First vs last day of a 23-day pre-competition training camp	6.38 ± 5.37	3.13 ± 2.23	not reported	Wilcoxon within-EG p = 0.141; Mann–Whitney on change scores EG vs CG p = 0.002	Cohen's d = 1.998 (between-group difference in change)	No (within-EG); Yes (between-group change)	Null	Non-randomised allocation (national-team starters vs substitutes); single-blinded. Wilcoxon signed-rank (within group) and Mann–Whitney U on pre–post differences (between groups); Cohen's d for the between-group comparison of changes. BOTH groups lost weight: EG 84.9 → 81.9 kg (p = 0.012; ≈3.5%), CG 84.9 → 83.5 kg (p = 0.080; ≈1.6%). POMS Turkish 58-item version, raw scores.
Ayar et al., 2023 [31]	Control group (self-selected 'traditional' weight loss methods; n = 8)	POMS – Depression–Dejection	First vs last day of a 23-day pre-competition training camp	9.38 ± 8.02	19.63 ± 13.23	not reported	Wilcoxon within-CG p = 0.012; between-group p = 0.002 (see EG row)	see EG row	Yes (within-CG)	Unfavourable	Control athletes also cut weight using traditional methods (sauna, excess exercise, food/fluid restriction). Mean ± SD with min–max (median) reported in the original.

Study (first author, year) [ref]	Group / condition	Outcome	Time point	Pre value (mean ± SD)	Post value (mean ± SD)	Change (absolute and/or %)	Test statistic and p value	Effect size (95% CI if reported)	Statistically significant (Yes / No)	Direction (Favourable / Unfavourable / Null)	Notes
Ayar et al., 2023 [31]	Experimental group (dietitian-supervised programme, 15–20% energy restriction; n = 8)	POMS – Anger–Hostility	First vs last day of a 23-day pre-competition training camp	10.88 ± 5.44	7.75 ± 5.52	not reported	Wilcoxon within-EG p = 0.058; Mann–Whitney on change scores EG vs CG p = 0.001	Cohen's d = 2.304 (between-group difference in change)	No (within-EG); Yes (between-group change)	Null	Non-randomised allocation (national-team starters vs substitutes); single-blinded. Wilcoxon signed-rank (within group) and Mann–Whitney U on pre–post differences (between groups); Cohen's d for the between-group comparison of changes. BOTH groups lost weight: EG 84.9 → 81.9 kg (p = 0.012; ≈3.5%), CG 84.9 → 83.5 kg (p = 0.080; ≈1.6%). POMS Turkish 58-item version, raw scores.
Ayar et al., 2023 [31]	Control group (self-selected 'traditional' weight loss methods; n = 8)	POMS – Anger–Hostility	First vs last day of a 23-day pre-competition training camp	9.50 ± 9.90	22.88 ± 10.62	not reported	Wilcoxon within-CG p = 0.012; between-group p = 0.001 (see EG row)	see EG row	Yes (within-CG)	Unfavourable	Control athletes also cut weight using traditional methods (sauna, excess exercise, food/fluid restriction). Mean ± SD with min–max (median) reported in the original.

Study (first author, year) [ref]	Group / condition	Outcome	Time point	Pre value (mean ± SD)	Post value (mean ± SD)	Change (absolute and/or %)	Test statistic and p value	Effect size (95% CI if reported)	Statistically significant (Yes / No)	Direction (Favourable / Unfavourable / Null)	Notes
Ayar et al., 2023 [31]	Experimental group (dietitian-supervised programme, 15–20% energy restriction; n = 8)	POMS – Fatigue–Stillness (Fatigue–Inertia)	First vs last day of a 23-day pre-competition training camp	5.63 ± 2.62	3.63 ± 2.83	not reported	Wilcoxon within-EG p = 0.041; Mann–Whitney on change scores EG vs CG p = 0.001	Cohen's d = 3.360 (between-group difference in change)	Yes (within-EG)	Favourable	Non-randomised allocation (national-team starters vs substitutes); single-blinded. Wilcoxon signed-rank (within group) and Mann–Whitney U on pre–post differences (between groups); Cohen's d for the between-group comparison of changes. BOTH groups lost weight: EG 84.9 → 81.9 kg (p = 0.012; ≈3.5%), CG 84.9 → 83.5 kg (p = 0.080; ≈1.6%). POMS Turkish 58-item version, raw scores.
Ayar et al., 2023 [31]	Control group (self-selected 'traditional' weight loss methods; n = 8)	POMS – Fatigue–Stillness (Fatigue–Inertia)	First vs last day of a 23-day pre-competition training camp	7.63 ± 4.78	15.13 ± 4.22	not reported	Wilcoxon within-CG p = 0.012; between-group p = 0.001 (see EG row)	see EG row	Yes (within-CG)	Unfavourable	Control athletes also cut weight using traditional methods (sauna, excess exercise, food/fluid restriction). Mean ± SD with min–max (median) reported in the original.

Study (first author, year) [ref]	Group / condition	Outcome	Time point	Pre value (mean ± SD)	Post value (mean ± SD)	Change (absolute and/or %)	Test statistic and p value	Effect size (95% CI if reported)	Statistically significant (Yes / No)	Direction (Favourable / Unfavourable / Null)	Notes
Ayar et al., 2023 [31]	Experimental group (dietitian-supervised programme, 15–20% energy restriction; n = 8)	POMS – Confusion–Bewilderment	First vs last day of a 23-day pre-competition training camp	8.00 ± 3.46	8.13 ± 4.70	not reported	Wilcoxon within-EG p = 1.000; Mann–Whitney on change scores EG vs CG p = 0.387	Cohen's d = 0.502 (between-group difference in change)	No (within-EG)	Null	Non-randomised allocation (national-team starters vs substitutes); single-blinded. Wilcoxon signed-rank (within group) and Mann–Whitney U on pre–post differences (between groups); Cohen's d for the between-group comparison of changes. BOTH groups lost weight: EG 84.9 → 81.9 kg (p = 0.012; ≈3.5%), CG 84.9 → 83.5 kg (p = 0.080; ≈1.6%). POMS Turkish 58-item version, raw scores.
Ayar et al., 2023 [31]	Control group (self-selected 'traditional' weight loss methods; n = 8)	POMS – Confusion–Bewilderment	First vs last day of a 23-day pre-competition training camp	8.38 ± 4.03	9.88 ± 5.62	not reported	Wilcoxon within-CG p = 0.246; between-group p = 0.387 (see EG row)	see EG row	No (within-CG)	Null	Control athletes also cut weight using traditional methods (sauna, excess exercise, food/fluid restriction). Mean ± SD with min–max (median) reported in the original.

Study (first author, year) [ref]	Group / condition	Outcome	Time point	Pre value (mean ± SD)	Post value (mean ± SD)	Change (absolute and/or %)	Test statistic and p value	Effect size (95% CI if reported)	Statistically significant (Yes / No)	Direction (Favourable / Unfavourable / Null)	Notes
Ayar et al., 2023 [31]	Experimental group (dietitian-supervised programme, 15–20% energy restriction; n = 8)	POMS – Vigor–Activity	First vs last day of a 23-day pre-competition training camp	18.75 ± 3.37	21.63 ± 3.29	not reported	Wilcoxon within-EG p = 0.021; Mann–Whitney on change scores EG vs CG p = 0.065	Cohen's d = 1.141 (between-group difference in change)	Yes (within-EG)	Favourable	Non-randomised allocation (national-team starters vs substitutes); single-blinded. Wilcoxon signed-rank (within group) and Mann–Whitney U on pre–post differences (between groups); Cohen's d for the between-group comparison of changes. BOTH groups lost weight: EG 84.9 → 81.9 kg (p = 0.012; ≈3.5%), CG 84.9 → 83.5 kg (p = 0.080; ≈1.6%). POMS Turkish 58-item version, raw scores. Between-group difference in change not significant (p = 0.065).
Ayar et al., 2023 [31]	Control group (self-selected 'traditional' weight loss methods; n = 8)	POMS – Vigor–Activity	First vs last day of a 23-day pre-competition training camp	17.38 ± 6.05	13.50 ± 6.12	not reported	Wilcoxon within-CG p = 0.205; between-group p = 0.065 (see EG row)	see EG row	No (within-CG)	Null	Control athletes also cut weight using traditional methods (sauna, excess exercise, food/fluid restriction). Mean ± SD with min–max (median) reported in the original.

Study (first author, year) [ref]	Group / condition	Outcome	Time point	Pre value (mean ± SD)	Post value (mean ± SD)	Change (absolute and/or %)	Test statistic and p value	Effect size (95% CI if reported)	Statistically significant (Yes / No)	Direction (Favourable / Unfavourable / Null)	Notes
Ayar et al., 2023 [31]	Experimental group (dietitian-supervised programme, 15–20% energy restriction; n = 8)	POMS – Total Mood Disturbance (TMD)	First vs last day of a 23-day pre-competition training camp	24.50 ± 17.03	11.75 ± 12.04	not reported	Wilcoxon within-EG p = 0.017; Mann–Whitney on change scores EG vs CG p = 0.001	Cohen's d = 2.773 (between-group difference in change)	Yes (within-EG)	Favourable	Non-randomised allocation (national-team starters vs substitutes); single-blinded. Wilcoxon signed-rank (within group) and Mann–Whitney U on pre–post differences (between groups); Cohen's d for the between-group comparison of changes. BOTH groups lost weight: EG 84.9 → 81.9 kg (p = 0.012; ≈3.5%), CG 84.9 → 83.5 kg (p = 0.080; ≈1.6%). POMS Turkish 58-item version, raw scores.
Ayar et al., 2023 [31]	Control group (self-selected 'traditional' weight loss methods; n = 8)	POMS – Total Mood Disturbance (TMD)	First vs last day of a 23-day pre-competition training camp	28.63 ± 30.70	71.88 ± 37.26	not reported	Wilcoxon within-CG p = 0.012; between-group p = 0.001 (see EG row)	see EG row	Yes (within-CG)	Unfavourable	Control athletes also cut weight using traditional methods (sauna, excess exercise, food/fluid restriction). Mean ± SD with min–max (median) reported in the original.

Study (first author, year) [ref]	Group / condition	Outcome	Time point	Pre value (mean ± SD)	Post value (mean ± SD)	Change (absolute and/or %)	Test statistic and p value	Effect size (95% CI if reported)	Statistically significant (Yes / No)	Direction (Favourable / Unfavourable / Null)	Notes
Brandt et al., 2018 [32]	RWL group (rapid weight loss; n = 9)	BRUMS – Anger	Training (30 d before) vs official weigh-in (24 h before bout); pre-bout (10 min before) and post-bout (10 min after) in Post column	2.0 (1.0; 4.5)	Weigh-in 0.0 (0.0; 2.0); pre-bout 0.0 (0.0; 4.0); post-bout 0.0 (0.0; 4.5)	not reported	Within-RWL: p = 0.24. Between groups (Mann–Whitney): training p = 0.48; weigh-in p = 0.037 (NWL > RWL; ES = 0.17); pre-bout p = 0.60; post-bout(?) p = 0.86	ES = Z/√N where reported (see stat column)	Yes (between groups at weigh-in only; NWL > RWL)	Favourable (lower anger in RWL than NWL at weigh-in)	Data are median (Q1; Q3), not mean ± SD. Non-randomised; NWL n = 3. Within-group: Friedman test; between-group: Mann–Whitney U. The 'weight loss' period was ~30 days (11.6 ± 3.5 kg). The fourth set of between-group p values is labelled 'before competition' a second time in the text; assumed to refer to post-bout. Abstract states that RWL was 'associated with high anger at the official weigh-in', whereas Results and Table 1 show higher anger in NWL (6.0 vs 0.0).
Brandt et al., 2018 [32]	NWL group (no rapid weight loss; n = 3)	BRUMS – Anger	Training (30 d before) vs official weigh-in (24 h before bout); pre-bout (10 min before) and post-bout (10 min after) in Post column	1.0 (0.0; 3.0)	Weigh-in 6.0 (1.0; 7.0); pre-bout 2.0 (0.0; 7.0); post-bout 0.0 (0.0; 2.0)	not reported	Within-NWL: p = 0.41	not reported	No	Null	Data are median (Q1; Q3), not mean ± SD. Non-randomised; NWL n = 3. Within-group: Friedman test; between-group: Mann–Whitney U. The 'weight loss' period was ~30 days (11.6 ± 3.5 kg).

Study (first author, year) [ref]	Group / condition	Outcome	Time point	Pre value (mean ± SD)	Post value (mean ± SD)	Change (absolute and/or %)	Test statistic and p value	Effect size (95% CI if reported)	Statistically significant (Yes / No)	Direction (Favourable / Unfavourable / Null)	Notes
Brandt et al., 2018 [32]	RWL group (rapid weight loss; n = 9)	BRUMS – Confusion	Training (30 d before) vs official weigh-in (24 h before bout); pre-bout (10 min before) and post-bout (10 min after) in Post column	1.0 (1.0; 2.0)	Weigh-in 0.0 (0.0; 0.5); pre-bout 0.0 (0.0; 0.0); post-bout 0.0 (0.0; 3.5)	not reported	Within-RWL: Friedman $\chi^2 = 8.081$, df = 3, p = 0.044; post hoc vs training: weigh-in p = 0.047 (ES = 0.16), pre-bout p = 0.016 (ES = 0.20). Between groups (Mann–Whitney): training p = 0.86; weigh-in p = 0.28; pre-bout p = 0.86; post-bout(?) p = 0.48	ES = Z/√N where reported (see stat column)	Yes (within-RWL, Friedman + post hoc)	Unclear (text and medians conflict)	Data are median (Q1; Q3), not mean ± SD. Non-randomised; NWL n = 3. Within-group: Friedman test; between-group: Mann–Whitney U. The 'weight loss' period was ~30 days (11.6 ± 3.5 kg). The fourth set of between-group p values is labelled 'before competition' a second time in the text; assumed to refer to post-bout. Ambiguity: text says RWL 'reported higher values than official weigh-in and before competition'; Table 1 medians show confusion LOWER at weigh-in (0.0) and pre-bout (0.0) than at training (1.0).
Brandt et al., 2018 [32]	NWL group (no rapid weight loss; n = 3)	BRUMS – Confusion	Training (30 d before) vs official weigh-in (24 h before bout); pre-bout (10 min before) and post-bout (10 min after) in Post column	1.0 (0.0; 4.0)	Weigh-in 2.0 (0.0; 2.0); pre-bout 0.0 (0.0; 0.0); post-bout 0.0 (0.0; 0.0)	not reported	Within-NWL: p = 0.19	not reported	No	Null	Data are median (Q1; Q3), not mean ± SD. Non-randomised; NWL n = 3. Within-group: Friedman test; between-group: Mann–Whitney U. The 'weight loss' period was ~30 days (11.6 ± 3.5 kg).

Study (first author, year) [ref]	Group / condition	Outcome	Time point	Pre value (mean ± SD)	Post value (mean ± SD)	Change (absolute and/or %)	Test statistic and p value	Effect size (95% CI if reported)	Statistically significant (Yes / No)	Direction (Favourable / Unfavourable / Null)	Notes
Brandt et al., 2018 [32]	RWL group (rapid weight loss; n = 9)	BRUMS – Depression	Training (30 d before) vs official weigh-in (24 h before bout); pre-bout (10 min before) and post-bout (10 min after) in Post column	0.0 (0.0; 0.5)	Weigh-in 0.0 (0.0; 0.0); pre-bout 0.0 (0.0; 0.0); post-bout 0.0 (0.0; 0.5)	not reported	Within-RWL: p = 0.19. Between groups (Mann–Whitney): training p = 0.86; weigh-in p = 1.00; pre-bout p = 1.00; post-bout(?) p = 0.60	ES = Z/√N where reported (see stat column)	No	Null	Data are median (Q1; Q3), not mean ± SD. Non-randomised; NWL n = 3. Within-group: Friedman test; between-group: Mann–Whitney U. The 'weight loss' period was ~30 days (11.6 ± 3.5 kg). The fourth set of between-group p values is labelled 'before competition' a second time in the text; assumed to refer to post-bout.
Brandt et al., 2018 [32]	NWL group (no rapid weight loss; n = 3)	BRUMS – Depression	Training (30 d before) vs official weigh-in (24 h before bout); pre-bout (10 min before) and post-bout (10 min after) in Post column	0.0 (0.0; 1.0)	Weigh-in 0.0 (0.0; 0.0); pre-bout 0.0 (0.0; 0.0); post-bout 0.0 (0.0; 0.0)	not reported	Within-NWL: p = 0.39	not reported	No	Null	Data are median (Q1; Q3), not mean ± SD. Non-randomised; NWL n = 3. Within-group: Friedman test; between-group: Mann–Whitney U. The 'weight loss' period was ~30 days (11.6 ± 3.5 kg).
Brandt et al., 2018 [32]	RWL group (rapid weight loss; n = 9)	BRUMS – Fatigue	Training (30 d before) vs official weigh-in (24 h before bout); pre-bout (10 min before) and post-bout (10 min after) in Post column	2.0 (0.5; 4.5)	Weigh-in 2.0 (0.0; 3.0); pre-bout 0.0 (0.0; 2.5); post-bout 2.0 (0.0; 5.5)	not reported	Within-RWL: p = 0.22. Between groups (Mann–Whitney): training p = 0.86; weigh-in p = 0.48; pre-bout p = 1.00; post-bout(?) p = 0.86	ES = Z/√N where reported (see stat column)	No	Null	Data are median (Q1; Q3), not mean ± SD. Non-randomised; NWL n = 3. Within-group: Friedman test; between-group: Mann–Whitney U. The 'weight loss' period was ~30 days (11.6 ± 3.5 kg). The fourth set of between-group p values is labelled 'before competition' a second time in the text; assumed to refer to post-bout.

Study (first author, year) [ref]	Group / condition	Outcome	Time point	Pre value (mean ± SD)	Post value (mean ± SD)	Change (absolute and/or %)	Test statistic and p value	Effect size (95% CI if reported)	Statistically significant (Yes / No)	Direction (Favourable / Unfavourable / Null)	Notes
Brandt et al., 2018 [32]	NWL group (no rapid weight loss; n = 3)	BRUMS – Fatigue	Training (30 d before) vs official weigh-in (24 h before bout); pre-bout (10 min before) and post-bout (10 min after) in Post column	3.0 (0.0; 4.0)	Weigh-in 4.0 (0.0; 5.0); pre-bout 0.0 (0.0; 3.0); post-bout 3.0 (0.0; 8.0)	not reported	Within-NWL: p = 0.43	not reported	No	Null	Data are median (Q1; Q3), not mean ± SD. Non-randomised; NWL n = 3. Within-group: Friedman test; between-group: Mann–Whitney U. The 'weight loss' period was ~30 days (11.6 ± 3.5 kg).
Brandt et al., 2018 [32]	RWL group (rapid weight loss; n = 9)	BRUMS – Tension	Training (30 d before) vs official weigh-in (24 h before bout); pre-bout (10 min before) and post-bout (10 min after) in Post column	3.0 (1.0; 7.5)	Weigh-in 4.0 (2.0; 5.0); pre-bout 3.0 (1.5; 4.0); post-bout 0.0 (0.0; 3.5)	not reported	Within-RWL: p = 0.15. Between groups (Mann–Whitney): training p = 0.48; weigh-in p = 0.37; pre-bout p = 0.21; post-bout(?) p = 0.48	ES = Z/√N where reported (see stat column)	No	Null	Data are median (Q1; Q3), not mean ± SD. Non-randomised; NWL n = 3. Within-group: Friedman test; between-group: Mann–Whitney U. The 'weight loss' period was ~30 days (11.6 ± 3.5 kg). The fourth set of between-group p values is labelled 'before competition' a second time in the text; assumed to refer to post-bout.
Brandt et al., 2018 [32]	NWL group (no rapid weight loss; n = 3)	BRUMS – Tension	Training (30 d before) vs official weigh-in (24 h before bout); pre-bout (10 min before) and post-bout (10 min after) in Post column	2.0 (2.0; 2.0)	Weigh-in 6.0 (2.0; 11.0); pre-bout 2.0 (0.0; 2.0); post-bout 3.0 (0.0; 5.0)	not reported	Within-NWL: p = 0.24	not reported	No	Null	Data are median (Q1; Q3), not mean ± SD. Non-randomised; NWL n = 3. Within-group: Friedman test; between-group: Mann–Whitney U. The 'weight loss' period was ~30 days (11.6 ± 3.5 kg).

Study (first author, year) [ref]	Group / condition	Outcome	Time point	Pre value (mean ± SD)	Post value (mean ± SD)	Change (absolute and/or %)	Test statistic and p value	Effect size (95% CI if reported)	Statistically significant (Yes / No)	Direction (Favourable / Unfavourable / Null)	Notes
Brandt et al., 2018 [32]	RWL group (rapid weight loss; n = 9)	BRUMS – Vigor	Training (30 d before) vs official weigh-in (24 h before bout); pre-bout (10 min before) and post-bout (10 min after) in Post column	13.0 (8.0; 16.0)	Weigh-in 15.0 (12.5; 16.0); pre-bout 16.0 (14.5; 16.0); post-bout 13.0 (9.5; 15.0)	not reported	Within-RWL: p = 0.25. Between groups (Mann–Whitney): training p = 0.86; weigh-in p = 0.28; pre-bout p = 0.86; post-bout(?) p = 0.28	ES = Z/√N where reported (see stat column)	No	Null	Data are median (Q1; Q3), not mean ± SD. Non-randomised; NWL n = 3. Within-group: Friedman test; between-group: Mann–Whitney U. The 'weight loss' period was ~30 days (11.6 ± 3.5 kg). The fourth set of between-group p values is labelled 'before competition' a second time in the text; assumed to refer to post-bout.
Brandt et al., 2018 [32]	NWL group (no rapid weight loss; n = 3)	BRUMS – Vigor	Training (30 d before) vs official weigh-in (24 h before bout); pre-bout (10 min before) and post-bout (10 min after) in Post column	11.0 (8.0; 16.0)	Weigh-in 12.0 (12.0; 15.0); pre-bout 15.0 (15.0; 16.0); post-bout 14.0 (13.0; 16.0)	not reported	Within-NWL: p = 0.56	not reported	No	Null	Data are median (Q1; Q3), not mean ± SD. Non-randomised; NWL n = 3. Within-group: Friedman test; between-group: Mann–Whitney U. The 'weight loss' period was ~30 days (11.6 ± 3.5 kg).

Study (first author, year) [ref]	Group / condition	Outcome	Time point	Pre value (mean ± SD)	Post value (mean ± SD)	Change (absolute and/or %)	Test statistic and p value	Effect size (95% CI if reported)	Statistically significant (Yes / No)	Direction (Favourable / Unfavourable / Null)	Notes
Brandt et al., 2018 [32]	RWL group (rapid weight loss; n = 9)	BRUMS – Total mood disturbance (TMD)	Training (30 d before) vs official weigh-in (24 h before bout); pre-bout (10 min before) and post-bout (10 min after) in Post column	−8.0 (−11.0; −4.0)*	Weigh-in 9.0 (5.0; 10.0); pre-bout 11.0 (2.0; 14.0); post-bout 3.0 (−2.5; 14.0)*	not reported	Within-RWL: Friedman χ^2 = 14.461, df = 3, p = 0.002; vs training: weigh-in p = 0.008 (ES = 0.22), pre-bout p = 0.008 (ES = 0.22), post-bout p = 0.011 (ES = 0.21). Between groups (Mann–Whitney): training p = 0.48; weigh-in p = 0.21; pre-bout p = 0.37; post-bout(?) p = 0.86	ES = Z/√N where reported (see stat column)	Yes (within-RWL, Friedman + post hoc)	Unfavourable	Data are median (Q1; Q3), not mean ± SD. Non-randomised; NWL n = 3. Within-group: Friedman test; between-group: Mann–Whitney U. The 'weight loss' period was ~30 days (11.6 ± 3.5 kg). The fourth set of between-group p values is labelled 'before competition' a second time in the text; assumed to refer to post-bout. *Minus signs are rendered as '2' in the PDF text layer (e.g., '210.0' = −10.0); negative values reconstructed and should be checked against the typeset table. Text states RWL 'reported lower values at 30 days before competition' than at later points. The printed TMD formula is 'vigor − (anger + confusion + depression + fatigue + tension)', i.e. inverted relative to the standard scoring, which makes the direction of 'disturbance' ambiguous.

Study (first author, year) [ref]	Group / condition	Outcome	Time point	Pre value (mean ± SD)	Post value (mean ± SD)	Change (absolute and/or %)	Test statistic and p value	Effect size (95% CI if reported)	Statistically significant (Yes / No)	Direction (Favourable / Unfavourable / Null)	Notes
Brandt et al., 2018 [32]	NWL group (no rapid weight loss; n = 3)	BRUMS – Total mood disturbance (TMD)	Training (30 d before) vs official weigh-in (24 h before bout); pre-bout (10 min before) and post-bout (10 min after) in Post column	−10.0 (−16.0; −7.0)*	Weigh-in 3.0 (−9.0; 9.0)*; pre-bout 3.0 (−4.0; 12.0)*; post-bout −2.0 (−3.0; 16.0)*	not reported	Within-NWL: $p = 0.12$	not reported	No	Null	Data are median (Q1; Q3), not mean ± SD. Non-randomised; NWL n = 3. Within-group: Friedman test; between-group: Mann–Whitney U. The 'weight loss' period was ~30 days (11.6 ± 3.5 kg).
Brandt et al., 2018 [32]	All fighters (RWL n = 9 + NWL n = 3)	Competition outcome (win/loss)	Official bout	not reported	10/12 won (83.3%): 7 by finish, 2 by TKO, 1 by decision; 2 lost (1 decision, 1 KO)	not reported	Outcomes by group NR; no test of RWL vs NWL on outcome reported. No mood differences between winners and losers ($p > 0.05$)	not reported	not tested	not reported	Authors conclude RWL 'did not have deleterious effects on performance', but no group-wise outcome data are provided.
Koral & Dosseville, 2009 [33]	Diet group – males (n = 5)	Squat jump (SJ) (cm)	T1 (4 wk before championship) vs T2 (1 d before championship)	58.6 ± 6.2	58.8 ± 4.8	not reported	Text: results did not differ between sessions; no SJ/CMJ ratio change. Table II marks diet T2 values with symbol a ('procedure main effect $p < 0.05$')	not reported	No (per text)	Null	Diet group: −4 MJ/day for 21 days + plastic-suit sweating during the last 6 days (≈4 weeks in total). MANOVA with Tukey post hoc; effects not reported separately per sex × group cell except via table symbols. Discrepancy between Table II symbols and text (see Mean power row). Males > females (sex effect).

Study (first author, year) [ref]	Group / condition	Outcome	Time point	Pre value (mean ± SD)	Post value (mean ± SD)	Change (absolute and/or %)	Test statistic and p value	Effect size (95% CI if reported)	Statistically significant (Yes / No)	Direction (Favourable / Unfavourable / Null)	Notes
Koral & Dosseville, 2009 [33]	Diet group – females (n = 5)	Squat jump (SJ) (cm)	T1 (4 wk before championship) vs T2 (1 d before championship)	45.6 ± 1.7	46.8 ± 2.9	not reported	Text: results did not differ between sessions; no SJ/CMJ ratio change. Table II marks diet T2 values with symbol a ('procedure main effect $p < 0.05$')	not reported	No (per text)	Null	Diet group: −4 MJ/day for 21 days + plastic-suit sweating during the last 6 days (≈4 weeks in total). MANOVA with Tukey post hoc; effects not reported separately per sex × group cell except via table symbols. Discrepancy between Table II symbols and text (see Mean power row). Males > females (sex effect).
Koral & Dosseville, 2009 [33]	Control group – males (n = 5)	Squat jump (SJ) (cm)	T1 (4 wk before championship) vs T2 (1 d before championship)	56.0 ± 6.3	54.6 ± 8.1	not reported	Text: results did not differ between sessions; no SJ/CMJ ratio change. Table II marks diet T2 values with symbol a ('procedure main effect $p < 0.05$')	not reported	No (per text)	Null	Diet group: −4 MJ/day for 21 days + plastic-suit sweating during the last 6 days (≈4 weeks in total). MANOVA with Tukey post hoc; effects not reported separately per sex × group cell except via table symbols. Discrepancy between Table II symbols and text (see Mean power row). Males > females (sex effect).

Study (first author, year) [ref]	Group / condition	Outcome	Time point	Pre value (mean ± SD)	Post value (mean ± SD)	Change (absolute and/or %)	Test statistic and p value	Effect size (95% CI if reported)	Statistically significant (Yes / No)	Direction (Favourable / Unfavourable / Null)	Notes
Koral & Dosseville, 2009 [33]	Control group – females (n = 5)	Squat jump (SJ) (cm)	T1 (4 wk before championship) vs T2 (1 d before championship)	43.0 ± 8.9	42.4 ± 7.0	not reported	Text: results did not differ between sessions; no SJ/CMJ ratio change. Table II marks diet T2 values with symbol a ('procedure main effect p < 0.05')	not reported	No (per text)	Null	Diet group: −4 MJ/day for 21 days + plastic-suit sweating during the last 6 days (≈4 weeks in total). MANOVA with Tukey post hoc; effects not reported separately per sex × group cell except via table symbols. Discrepancy between Table II symbols and text (see Mean power row). Males > females (sex effect).
Koral & Dosseville, 2009 [33]	Diet group – males (n = 5)	Countermovement jump (CMJ) (cm)	T1 (4 wk before championship) vs T2 (1 d before championship)	61.6 ± 5.0	59.0 ± 6.4	not reported	Text: results did not differ between sessions; no SJ/CMJ ratio change. Table II marks diet T2 values with symbol a ('procedure main effect p < 0.05')	not reported	No (per text)	Null	Diet group: −4 MJ/day for 21 days + plastic-suit sweating during the last 6 days (≈4 weeks in total). MANOVA with Tukey post hoc; effects not reported separately per sex × group cell except via table symbols. Discrepancy between Table II symbols and text (see Mean power row). Males > females (sex effect).

Study (first author, year) [ref]	Group / condition	Outcome	Time point	Pre value (mean ± SD)	Post value (mean ± SD)	Change (absolute and/or %)	Test statistic and p value	Effect size (95% CI if reported)	Statistically significant (Yes / No)	Direction (Favourable / Unfavourable / Null)	Notes
Koral & Dosseville, 2009 [33]	Diet group – females (n = 5)	Countermovement jump (CMJ) (cm)	T1 (4 wk before championship) vs T2 (1 d before championship)	47.0 ± 3.9	47.4 ± 3.1	not reported	Text: results did not differ between sessions; no SJ/CMJ ratio change. Table II marks diet T2 values with symbol a ('procedure main effect $p < 0.05$')	not reported	No (per text)	Null	Diet group: −4 MJ/day for 21 days + plastic-suit sweating during the last 6 days (≈4 weeks in total). MANOVA with Tukey post hoc; effects not reported separately per sex × group cell except via table symbols. Discrepancy between Table II symbols and text (see Mean power row). Males > females (sex effect).
Koral & Dosseville, 2009 [33]	Control group – males (n = 5)	Countermovement jump (CMJ) (cm)	T1 (4 wk before championship) vs T2 (1 d before championship)	55.8 ± 7.9	54.8 ± 6.6	not reported	Text: results did not differ between sessions; no SJ/CMJ ratio change. Table II marks diet T2 values with symbol a ('procedure main effect $p < 0.05$')	not reported	No (per text)	Null	Diet group: −4 MJ/day for 21 days + plastic-suit sweating during the last 6 days (≈4 weeks in total). MANOVA with Tukey post hoc; effects not reported separately per sex × group cell except via table symbols. Discrepancy between Table II symbols and text (see Mean power row). Males > females (sex effect).

Study (first author, year) [ref]	Group / condition	Outcome	Time point	Pre value (mean ± SD)	Post value (mean ± SD)	Change (absolute and/or %)	Test statistic and p value	Effect size (95% CI if reported)	Statistically significant (Yes / No)	Direction (Favourable / Unfavourable / Null)	Notes
Koral & Dosseville, 2009 [33]	Control group – females (n = 5)	Countermovement jump (CMJ) (cm)	T1 (4 wk before championship) vs T2 (1 d before championship)	43.6 ± 8.6	42.8 ± 7.2	not reported	Text: results did not differ between sessions; no SJ/CMJ ratio change. Table II marks diet T2 values with symbol a ('procedure main effect $p < 0.05$')	not reported	No (per text)	Null	Diet group: −4 MJ/day for 21 days + plastic-suit sweating during the last 6 days (≈4 weeks in total). MANOVA with Tukey post hoc; effects not reported separately per sex × group cell except via table symbols. Discrepancy between Table II symbols and text (see Mean power row). Males > females (sex effect).
Koral & Dosseville, 2009 [33]	Diet group – males (n = 5)	Mean power, 10 rowing movements at 70% 1-MR (MP) (W)	T1 (4 wk before championship) vs T2 (1 d before championship)	213.0 ± 17.2	208.1 ± 20.7	Diet group: −3.6 W (Discussion, pooled sexes)	Text: results did not differ between sessions; Table II marks diet T2 values with symbol a ('procedure main effect $p < 0.05$')	not reported	No (per text)	Null	Diet group: −4 MJ/day for 21 days + plastic-suit sweating during the last 6 days (≈4 weeks in total). MANOVA with Tukey post hoc; effects not reported separately per sex × group cell except via table symbols. Discrepancy: Table II attaches a significance symbol to diet-group T2 values while the text states no difference between sessions. Males > females (sex effect).

Study (first author, year) [ref]	Group / condition	Outcome	Time point	Pre value (mean ± SD)	Post value (mean ± SD)	Change (absolute and/or %)	Test statistic and p value	Effect size (95% CI if reported)	Statistically significant (Yes / No)	Direction (Favourable / Unfavourable / Null)	Notes
Koral & Dosseville, 2009 [33]	Diet group – females (n = 5)	Mean power, 10 rowing movements at 70% 1-MR (MP) (W)	T1 (4 wk before championship) vs T2 (1 d before championship)	179.5 ± 21.1	177.8 ± 25.0	Diet group: −3.6 W (Discussion, pooled sexes)	Text: results did not differ between sessions; Table II marks diet T2 values with symbol a ('procedure main effect $p < 0.05$')	not reported	No (per text)	Null	Diet group: −4 MJ/day for 21 days + plastic-suit sweating during the last 6 days (≈4 weeks in total). MANOVA with Tukey post hoc; effects not reported separately per sex × group cell except via table symbols. Discrepancy: Table II attaches a significance symbol to diet-group T2 values while the text states no difference between sessions. Males > females (sex effect).
Koral & Dosseville, 2009 [33]	Control group – males (n = 5)	Mean power, 10 rowing movements at 70% 1-MR (MP) (W)	T1 (4 wk before championship) vs T2 (1 d before championship)	209.6 ± 10.3	221.6 ± 16.6	Control group: +20.6 W (Discussion, pooled sexes)	Text: results did not differ between sessions; Table II marks diet T2 values with symbol a ('procedure main effect $p < 0.05$')	not reported	No (per text)	Null	Diet group: −4 MJ/day for 21 days + plastic-suit sweating during the last 6 days (≈4 weeks in total). MANOVA with Tukey post hoc; effects not reported separately per sex × group cell except via table symbols. Discrepancy: Table II attaches a significance symbol to diet-group T2 values while the text states no difference between sessions. Males > females (sex effect).

Study (first author, year) [ref]	Group / condition	Outcome	Time point	Pre value (mean ± SD)	Post value (mean ± SD)	Change (absolute and/or %)	Test statistic and p value	Effect size (95% CI if reported)	Statistically significant (Yes / No)	Direction (Favourable / Unfavourable / Null)	Notes
Koral & Dosseville, 2009 [33]	Control group – females (n = 5)	Mean power, 10 rowing movements at 70% 1-MR (MP) (W)	T1 (4 wk before championship) vs T2 (1 d before championship)	179.8 ± 18.5	204.4 ± 31.1	Control group: +20.6 W (Discussion, pooled sexes)	Text: results did not differ between sessions; Table II marks diet T2 values with symbol a ('procedure main effect $p < 0.05$')	not reported	No (per text)	Null	Diet group: −4 MJ/day for 21 days + plastic-suit sweating during the last 6 days (≈4 weeks in total). MANOVA with Tukey post hoc; effects not reported separately per sex × group cell except via table symbols. Discrepancy: Table II attaches a significance symbol to diet-group T2 values while the text states no difference between sessions. Males > females (sex effect).
Koral & Dosseville, 2009 [33]	Diet group – males (n = 5)	Tokui-waza repetitions in 5 s (TW-5) (n)	T1 (4 wk before championship) vs T2 (1 d before championship)	8.8 ± 0.8	8.8 ± 0.4	not reported	No significant difference between sessions for either group (F and p NR)	not reported	No	Null	Diet group: −4 MJ/day for 21 days + plastic-suit sweating during the last 6 days (≈4 weeks in total). MANOVA with Tukey post hoc; effects not reported separately per sex × group cell except via table symbols.
Koral & Dosseville, 2009 [33]	Diet group – females (n = 5)	Tokui-waza repetitions in 5 s (TW-5) (n)	T1 (4 wk before championship) vs T2 (1 d before championship)	8.3 ± 0.4	8.2 ± 0.8	not reported	No significant difference between sessions for either group (F and p NR)	not reported	No	Null	Diet group: −4 MJ/day for 21 days + plastic-suit sweating during the last 6 days (≈4 weeks in total). MANOVA with Tukey post hoc; effects not reported separately per sex × group cell except via table symbols.

Study (first author, year) [ref]	Group / condition	Outcome	Time point	Pre value (mean ± SD)	Post value (mean ± SD)	Change (absolute and/or %)	Test statistic and p value	Effect size (95% CI if reported)	Statistically significant (Yes / No)	Direction (Favourable / Unfavourable / Null)	Notes
Koral & Dosseville, 2009 [33]	Control group – males (n = 5)	Tokui-waza repetitions in 5 s (TW-5) (n)	T1 (4 wk before championship) vs T2 (1 d before championship)	8.2 ± 0.4	8.2 ± 0.4	not reported	No significant difference between sessions for either group (F and p NR)	not reported	No	Null	Diet group: −4 MJ/day for 21 days + plastic-suit sweating during the last 6 days (≈4 weeks in total). MANOVA with Tukey post hoc; effects not reported separately per sex × group cell except via table symbols.
Koral & Dosseville, 2009 [33]	Control group – females (n = 5)	Tokui-waza repetitions in 5 s (TW-5) (n)	T1 (4 wk before championship) vs T2 (1 d before championship)	8.8 ± 0.4	8.6 ± 0.5	not reported	No significant difference between sessions for either group (F and p NR)	not reported	No	Null	Diet group: −4 MJ/day for 21 days + plastic-suit sweating during the last 6 days (≈4 weeks in total). MANOVA with Tukey post hoc; effects not reported separately per sex × group cell except via table symbols.
Koral & Dosseville, 2009 [33]	Diet group – males (n = 5)	Tokui-waza repetitions in 30 s (TW-30) (n)	T1 (4 wk before championship) vs T2 (1 d before championship)	48.4 ± 5.4	42.6 ± 5.1	Diet group ≈ −6% (Discussion); abstract: −4.5 ± 2.7 (sign rendered as '7')	Procedure main effect $p < 0.01$ (text); group × procedure interaction $p < 0.05$ (text and Table II, symbol c)	not reported	Yes (interaction; post hoc per sex NR)	Unfavourable	Diet group: −4 MJ/day for 21 days + plastic-suit sweating during the last 6 days (≈4 weeks in total). MANOVA with Tukey post hoc; effects not reported separately per sex × group cell except via table symbols. Table II symbol b ('sex main effect $p < 0.05$') is also attached to diet T2 values.

Study (first author, year) [ref]	Group / condition	Outcome	Time point	Pre value (mean ± SD)	Post value (mean ± SD)	Change (absolute and/or %)	Test statistic and p value	Effect size (95% CI if reported)	Statistically significant (Yes / No)	Direction (Favourable / Unfavourable / Null)	Notes
Koral & Dosseville, 2009 [33]	Diet group – females (n = 5)	Tokui-waza repetitions in 30 s (TW-30) (n)	T1 (4 wk before championship) vs T2 (1 d before championship)	40.0 ± 2.3	36.8 ± 3.8	Diet group ≈ −6% (Discussion); abstract: −4.5 ± 2.7 (sign rendered as '7')	Procedure main effect $p < 0.01$ (text); group × procedure interaction $p < 0.05$ (text and Table II, symbol c)	not reported	Yes (interaction; post hoc per sex NR)	Unfavourable	Diet group: −4 MJ/day for 21 days + plastic-suit sweating during the last 6 days (≈4 weeks in total). MANOVA with Tukey post hoc; effects not reported separately per sex × group cell except via table symbols. Table II symbol b ('sex main effect p < 0.05') is also attached to diet T2 values.
Koral & Dosseville, 2009 [33]	Control group – males (n = 5)	Tokui-waza repetitions in 30 s (TW-30) (n)	T1 (4 wk before championship) vs T2 (1 d before championship)	44.4 ± 7.2	43.0 ± 7.3	Control ≈ −2% (Discussion)	Procedure main effect $p < 0.01$ (text); group × procedure interaction $p < 0.05$ (text and Table II, symbol c)	not reported	No (within-control, NR)	Null	Diet group: −4 MJ/day for 21 days + plastic-suit sweating during the last 6 days (≈4 weeks in total). MANOVA with Tukey post hoc; effects not reported separately per sex × group cell except via table symbols.
Koral & Dosseville, 2009 [33]	Control group – females (n = 5)	Tokui-waza repetitions in 30 s (TW-30) (n)	T1 (4 wk before championship) vs T2 (1 d before championship)	45.0 ± 4.9	43.0 ± 3.7	Control ≈ −2% (Discussion)	Procedure main effect $p < 0.01$ (text); group × procedure interaction $p < 0.05$ (text and Table II, symbol c)	not reported	No (within-control, NR)	Null	Diet group: −4 MJ/day for 21 days + plastic-suit sweating during the last 6 days (≈4 weeks in total). MANOVA with Tukey post hoc; effects not reported separately per sex × group cell except via table symbols.

Study (first author, year) [ref]	Group / condition	Outcome	Time point	Pre value (mean ± SD)	Post value (mean ± SD)	Change (absolute and/or %)	Test statistic and p value	Effect size (95% CI if reported)	Statistically significant (Yes / No)	Direction (Favourable / Unfavourable / Null)	Notes
Koral & Dosseville, 2009 [33]	Diet group – males (n = 5)	POMS – Tension	T1 (4 wk before championship) vs T2 (1 d before championship)	46.5 ± 1.1	48.0 ± 2.1	not reported	2 × 2 × 2 × 6 MANOVA: overall POMS procedure main effect $p < 0.01$; procedure × sex × group interaction $p < 0.05$ flagged (symbol a) only for diet-group cells (this cell not flagged)	not reported	No	Null	Diet group: −4 MJ/day for 21 days + plastic-suit sweating during the last 6 days (≈4 weeks in total). MANOVA with Tukey post hoc; effects not reported separately per sex × group cell except via table symbols. Dispersion values (e.g., ±0.4 on T-score-type values) are implausibly small for an SD and may be SEs; the article labels them 's'. Abstract gives within-group differences with signs rendered as '7' (e.g., confusion '714.6 ± 7.9', tension '710.1 ± 12.5', vigour '11.3 ± 8.5'), apparently expressed as T1 − T2.

Study (first author, year) [ref]	Group / condition	Outcome	Time point	Pre value (mean ± SD)	Post value (mean ± SD)	Change (absolute and/or %)	Test statistic and p value	Effect size (95% CI if reported)	Statistically significant (Yes / No)	Direction (Favourable / Unfavourable / Null)	Notes
Koral & Dosseville, 2009 [33]	Diet group – females (n = 5)	POMS – Tension	T1 (4 wk before championship) vs T2 (1 d before championship)	34.0 ± 2.0	52.8 ± 2.0	not reported	2 × 2 × 2 × 6 MANOVA: overall POMS procedure main effect p < 0.01; procedure × sex × group interaction p < 0.05 flagged (symbol a) only for diet-group cells	not reported	Yes (procedure × sex × group interaction, p < 0.05)	Unfavourable	Diet group: −4 MJ/day for 21 days + plastic-suit sweating during the last 6 days (≈4 weeks in total). MANOVA with Tukey post hoc; effects not reported separately per sex × group cell except via table symbols. Dispersion values (e.g., ±0.4 on T-score-type values) are implausibly small for an SD and may be SEs; the article labels them 's'. Abstract gives within-group differences with signs rendered as '7' (e.g., confusion '714.6 ± 7.9', tension '710.1 ± 12.5', vigour '11.3 ± 8.5'), apparently expressed as T1 − T2.

Study (first author, year) [ref]	Group / condition	Outcome	Time point	Pre value (mean ± SD)	Post value (mean ± SD)	Change (absolute and/or %)	Test statistic and p value	Effect size (95% CI if reported)	Statistically significant (Yes / No)	Direction (Favourable / Unfavourable / Null)	Notes
Koral & Dosseville, 2009 [33]	Control group – males (n = 5)	POMS – Tension	T1 (4 wk before championship) vs T2 (1 d before championship)	44.2 ± 2.2	51.4 ± 1.8	not reported	2 × 2 × 2 × 6 MANOVA: overall POMS procedure main effect $p < 0.01$; procedure × sex × group interaction $p < 0.05$ flagged (symbol a) only for diet-group cells (this cell not flagged)	not reported	No	Null	Diet group: −4 MJ/day for 21 days + plastic-suit sweating during the last 6 days (≈4 weeks in total). MANOVA with Tukey post hoc; effects not reported separately per sex × group cell except via table symbols. Dispersion values (e.g., ±0.4 on T-score-type values) are implausibly small for an SD and may be SEs; the article labels them 's'. Control-group changes of similar size (e.g., anger in control females 45.5→55.6) were not flagged as significant.
Koral & Dosseville, 2009 [33]	Control group – females (n = 5)	POMS – Tension	T1 (4 wk before championship) vs T2 (1 d before championship)	42.8 ± 1.7	43.9 ± 1.9	not reported	2 × 2 × 2 × 6 MANOVA: overall POMS procedure main effect $p < 0.01$; procedure × sex × group interaction $p < 0.05$ flagged (symbol a) only for diet-group cells (this cell not flagged)	not reported	No	Null	Diet group: −4 MJ/day for 21 days + plastic-suit sweating during the last 6 days (≈4 weeks in total). MANOVA with Tukey post hoc; effects not reported separately per sex × group cell except via table symbols. Dispersion values (e.g., ±0.4 on T-score-type values) are implausibly small for an SD and may be SEs; the article labels them 's'. Control-group changes of similar size (e.g., anger in control females 45.5→55.6) were not flagged as significant.

Study (first author, year) [ref]	Group / condition	Outcome	Time point	Pre value (mean ± SD)	Post value (mean ± SD)	Change (absolute and/or %)	Test statistic and p value	Effect size (95% CI if reported)	Statistically significant (Yes / No)	Direction (Favourable / Unfavourable / Null)	Notes
Koral & Dosseville, 2009 [33]	Diet group – males (n = 5)	POMS – Depression	T1 (4 wk before championship) vs T2 (1 d before championship)	34.4 ± 2.3	41.0 ± 1.6	not reported	2 × 2 × 2 × 6 MANOVA: overall POMS procedure main effect p < 0.01; procedure × sex × group interaction p < 0.05 flagged (symbol a) only for diet-group cells (this cell not flagged)	not reported	No	Null	Diet group: −4 MJ/day for 21 days + plastic-suit sweating during the last 6 days (≈4 weeks in total). MANOVA with Tukey post hoc; effects not reported separately per sex × group cell except via table symbols. Dispersion values (e.g., ±0.4 on T-score-type values) are implausibly small for an SD and may be SEs; the article labels them 's'.
Koral & Dosseville, 2009 [33]	Diet group – females (n = 5)	POMS – Depression	T1 (4 wk before championship) vs T2 (1 d before championship)	36.2 ± 0.9	42.8 ± 1.2	not reported	2 × 2 × 2 × 6 MANOVA: overall POMS procedure main effect p < 0.01; procedure × sex × group interaction p < 0.05 flagged (symbol a) only for diet-group cells (this cell not flagged)	not reported	No	Null	Diet group: −4 MJ/day for 21 days + plastic-suit sweating during the last 6 days (≈4 weeks in total). MANOVA with Tukey post hoc; effects not reported separately per sex × group cell except via table symbols. Dispersion values (e.g., ±0.4 on T-score-type values) are implausibly small for an SD and may be SEs; the article labels them 's'.

Study (first author, year) [ref]	Group / condition	Outcome	Time point	Pre value (mean ± SD)	Post value (mean ± SD)	Change (absolute and/or %)	Test statistic and p value	Effect size (95% CI if reported)	Statistically significant (Yes / No)	Direction (Favourable / Unfavourable / Null)	Notes
Koral & Dosseville, 2009 [33]	Control group – males (n = 5)	POMS – Depression	T1 (4 wk before championship) vs T2 (1 d before championship)	32.4 ± 3.0	39.4 ± 1.4	not reported	2 × 2 × 2 × 6 MANOVA: overall POMS procedure main effect $p < 0.01$; procedure × sex × group interaction $p < 0.05$ flagged (symbol a) only for diet-group cells (this cell not flagged)	not reported	No	Null	Diet group: −4 MJ/day for 21 days + plastic-suit sweating during the last 6 days (≈4 weeks in total). MANOVA with Tukey post hoc; effects not reported separately per sex × group cell except via table symbols. Dispersion values (e.g., ±0.4 on T-score-type values) are implausibly small for an SD and may be SEs; the article labels them 's'. Control-group changes of similar size (e.g., anger in control females 45.5→55.6) were not flagged as significant.
Koral & Dosseville, 2009 [33]	Control group – females (n = 5)	POMS – Depression	T1 (4 wk before championship) vs T2 (1 d before championship)	37.3 ± 2.9	40.4 ± 1.1	not reported	2 × 2 × 2 × 6 MANOVA: overall POMS procedure main effect $p < 0.01$; procedure × sex × group interaction $p < 0.05$ flagged (symbol a) only for diet-group cells (this cell not flagged)	not reported	No	Null	Diet group: −4 MJ/day for 21 days + plastic-suit sweating during the last 6 days (≈4 weeks in total). MANOVA with Tukey post hoc; effects not reported separately per sex × group cell except via table symbols. Dispersion values (e.g., ±0.4 on T-score-type values) are implausibly small for an SD and may be SEs; the article labels them 's'. Control-group changes of similar size (e.g., anger in control females 45.5→55.6) were not flagged as significant.

Study (first author, year) [ref]	Group / condition	Outcome	Time point	Pre value (mean ± SD)	Post value (mean ± SD)	Change (absolute and/or %)	Test statistic and p value	Effect size (95% CI if reported)	Statistically significant (Yes / No)	Direction (Favourable / Unfavourable / Null)	Notes
Koral & Dosseville, 2009 [33]	Diet group – males (n = 5)	POMS – Anger	T1 (4 wk before championship) vs T2 (1 d before championship)	40.3 ± 0.8	48.2 ± 2.6	not reported	2 × 2 × 2 × 6 MANOVA: overall POMS procedure main effect p < 0.01; procedure × sex × group interaction p < 0.05 flagged (symbol a) only for diet-group cells (this cell not flagged)	not reported	No	Null	Diet group: −4 MJ/day for 21 days + plastic-suit sweating during the last 6 days (≈4 weeks in total). MANOVA with Tukey post hoc; effects not reported separately per sex × group cell except via table symbols. Dispersion values (e.g., ±0.4 on T-score-type values) are implausibly small for an SD and may be SEs; the article labels them 's'.
Koral & Dosseville, 2009 [33]	Diet group – females (n = 5)	POMS – Anger	T1 (4 wk before championship) vs T2 (1 d before championship)	42.5 ± 0.9	43.5 ± 1.1	not reported	2 × 2 × 2 × 6 MANOVA: overall POMS procedure main effect p < 0.01; procedure × sex × group interaction p < 0.05 flagged (symbol a) only for diet-group cells (this cell not flagged)	not reported	No	Null	Diet group: −4 MJ/day for 21 days + plastic-suit sweating during the last 6 days (≈4 weeks in total). MANOVA with Tukey post hoc; effects not reported separately per sex × group cell except via table symbols. Dispersion values (e.g., ±0.4 on T-score-type values) are implausibly small for an SD and may be SEs; the article labels them 's'.

Study (first author, year) [ref]	Group / condition	Outcome	Time point	Pre value (mean ± SD)	Post value (mean ± SD)	Change (absolute and/or %)	Test statistic and p value	Effect size (95% CI if reported)	Statistically significant (Yes / No)	Direction (Favourable / Unfavourable / Null)	Notes
Koral & Dosseville, 2009 [33]	Control group – males (n = 5)	POMS – Anger	T1 (4 wk before championship) vs T2 (1 d before championship)	42.1 ± 1.2	47.7 ± 0.7	not reported	2 × 2 × 2 × 6 MANOVA: overall POMS procedure main effect $p < 0.01$; procedure × sex × group interaction $p < 0.05$ flagged (symbol a) only for diet-group cells (this cell not flagged)	not reported	No	Null	Diet group: −4 MJ/day for 21 days + plastic-suit sweating during the last 6 days (≈4 weeks in total). MANOVA with Tukey post hoc; effects not reported separately per sex × group cell except via table symbols. Dispersion values (e.g., ±0.4 on T-score-type values) are implausibly small for an SD and may be SEs; the article labels them 's'. Control-group changes of similar size (e.g., anger in control females 45.5→55.6) were not flagged as significant.
Koral & Dosseville, 2009 [33]	Control group – females (n = 5)	POMS – Anger	T1 (4 wk before championship) vs T2 (1 d before championship)	45.5 ± 1.1	55.6 ± 2.0	not reported	2 × 2 × 2 × 6 MANOVA: overall POMS procedure main effect $p < 0.01$; procedure × sex × group interaction $p < 0.05$ flagged (symbol a) only for diet-group cells (this cell not flagged)	not reported	No	Null	Diet group: −4 MJ/day for 21 days + plastic-suit sweating during the last 6 days (≈4 weeks in total). MANOVA with Tukey post hoc; effects not reported separately per sex × group cell except via table symbols. Dispersion values (e.g., ±0.4 on T-score-type values) are implausibly small for an SD and may be SEs; the article labels them 's'. Control-group changes of similar size (e.g., anger in control females 45.5→55.6) were not flagged as significant.

Study (first author, year) [ref]	Group / condition	Outcome	Time point	Pre value (mean ± SD)	Post value (mean ± SD)	Change (absolute and/or %)	Test statistic and p value	Effect size (95% CI if reported)	Statistically significant (Yes / No)	Direction (Favourable / Unfavourable / Null)	Notes
Koral & Dosseville, 2009 [33]	Diet group – males (n = 5)	POMS – Vigour	T1 (4 wk before championship) vs T2 (1 d before championship)	58.4 ± 0.4	48.6 ± 0.8	not reported	2 × 2 × 2 × 6 MANOVA: overall POMS procedure main effect $p < 0.01$; procedure × sex × group interaction $p < 0.05$ flagged (symbol a) only for diet-group cells	not reported	Yes (procedure × sex × group interaction, $p < 0.05$)	Unfavourable	Diet group: −4 MJ/day for 21 days + plastic-suit sweating during the last 6 days (≈4 weeks in total). MANOVA with Tukey post hoc; effects not reported separately per sex × group cell except via table symbols. Dispersion values (e.g., ±0.4 on T-score-type values) are implausibly small for an SD and may be SEs; the article labels them 's'. Abstract gives within-group differences with signs rendered as '7' (e.g., confusion '714.6 ± 7.9', tension '710.1 ± 12.5', vigour '11.3 ± 8.5'), apparently expressed as T1 − T2.

Study (first author, year) [ref]	Group / condition	Outcome	Time point	Pre value (mean ± SD)	Post value (mean ± SD)	Change (absolute and/or %)	Test statistic and p value	Effect size (95% CI if reported)	Statistically significant (Yes / No)	Direction (Favourable / Unfavourable / Null)	Notes
Koral & Dosseville, 2009 [33]	Diet group – females (n = 5)	POMS – Vigour	T1 (4 wk before championship) vs T2 (1 d before championship)	59.4 ± 0.7	48.6 ± 0.5	not reported	2 × 2 × 2 × 6 MANOVA: overall POMS procedure main effect p < 0.01; procedure × sex × group interaction p < 0.05 flagged (symbol a) only for diet-group cells	not reported	Yes (procedure × sex × group interaction, p < 0.05)	Unfavourable	Diet group: −4 MJ/day for 21 days + plastic-suit sweating during the last 6 days (≈4 weeks in total). MANOVA with Tukey post hoc; effects not reported separately per sex × group cell except via table symbols. Dispersion values (e.g., ±0.4 on T-score-type values) are implausibly small for an SD and may be SEs; the article labels them 's'. Abstract gives within-group differences with signs rendered as '7' (e.g., confusion '714.6 ± 7.9', tension '710.1 ± 12.5', vigour '11.3 ± 8.5'), apparently expressed as T1 − T2.

Study (first author, year) [ref]	Group / condition	Outcome	Time point	Pre value (mean ± SD)	Post value (mean ± SD)	Change (absolute and/or %)	Test statistic and p value	Effect size (95% CI if reported)	Statistically significant (Yes / No)	Direction (Favourable / Unfavourable / Null)	Notes
Koral & Dosseville, 2009 [33]	Control group – males (n = 5)	POMS – Vigour	T1 (4 wk before championship) vs T2 (1 d before championship)	53.3 ± 0.8	51.0 ± 0.4	not reported	2 × 2 × 2 × 6 MANOVA: overall POMS procedure main effect p < 0.01; procedure × sex × group interaction p < 0.05 flagged (symbol a) only for diet-group cells (this cell not flagged)	not reported	No	Null	Diet group: −4 MJ/day for 21 days + plastic-suit sweating during the last 6 days (≈4 weeks in total). MANOVA with Tukey post hoc; effects not reported separately per sex × group cell except via table symbols. Dispersion values (e.g., ±0.4 on T-score-type values) are implausibly small for an SD and may be SEs; the article labels them 's'. Control-group changes of similar size (e.g., anger in control females 45.5→55.6) were not flagged as significant.
Koral & Dosseville, 2009 [33]	Control group – females (n = 5)	POMS – Vigour	T1 (4 wk before championship) vs T2 (1 d before championship)	58.1 ± 0.4	52.4 ± 0.8	not reported	2 × 2 × 2 × 6 MANOVA: overall POMS procedure main effect p < 0.01; procedure × sex × group interaction p < 0.05 flagged (symbol a) only for diet-group cells (this cell not flagged)	not reported	No	Null	Diet group: −4 MJ/day for 21 days + plastic-suit sweating during the last 6 days (≈4 weeks in total). MANOVA with Tukey post hoc; effects not reported separately per sex × group cell except via table symbols. Dispersion values (e.g., ±0.4 on T-score-type values) are implausibly small for an SD and may be SEs; the article labels them 's'. Control-group changes of similar size (e.g., anger in control females 45.5→55.6) were not flagged as significant.

Study (first author, year) [ref]	Group / condition	Outcome	Time point	Pre value (mean ± SD)	Post value (mean ± SD)	Change (absolute and/or %)	Test statistic and p value	Effect size (95% CI if reported)	Statistically significant (Yes / No)	Direction (Favourable / Unfavourable / Null)	Notes
Koral & Dosseville, 2009 [33]	Diet group – males (n = 5)	POMS – Fatigue	T1 (4 wk before championship) vs T2 (1 d before championship)	53.0 ± 1.2	55.6 ± 1.7	not reported	2 × 2 × 2 × 6 MANOVA: overall POMS procedure main effect $p < 0.01$; procedure × sex × group interaction $p < 0.05$ flagged (symbol a) only for diet-group cells (this cell not flagged)	not reported	No	Null	Diet group: −4 MJ/day for 21 days + plastic-suit sweating during the last 6 days (≈4 weeks in total). MANOVA with Tukey post hoc; effects not reported separately per sex × group cell except via table symbols. Dispersion values (e.g., ±0.4 on T-score-type values) are implausibly small for an SD and may be SEs; the article labels them 's'.
Koral & Dosseville, 2009 [33]	Diet group – females (n = 5)	POMS – Fatigue	T1 (4 wk before championship) vs T2 (1 d before championship)	43.8 ± 0.9	52.8 ± 2.3	not reported	2 × 2 × 2 × 6 MANOVA: overall POMS procedure main effect $p < 0.01$; procedure × sex × group interaction $p < 0.05$ flagged (symbol a) only for diet-group cells (this cell not flagged)	not reported	No	Null	Diet group: −4 MJ/day for 21 days + plastic-suit sweating during the last 6 days (≈4 weeks in total). MANOVA with Tukey post hoc; effects not reported separately per sex × group cell except via table symbols. Dispersion values (e.g., ±0.4 on T-score-type values) are implausibly small for an SD and may be SEs; the article labels them 's'.

Study (first author, year) [ref]	Group / condition	Outcome	Time point	Pre value (mean ± SD)	Post value (mean ± SD)	Change (absolute and/or %)	Test statistic and p value	Effect size (95% CI if reported)	Statistically significant (Yes / No)	Direction (Favourable / Unfavourable / Null)	Notes
Koral & Dosseville, 2009 [33]	Control group – males (n = 5)	POMS – Fatigue	T1 (4 wk before championship) vs T2 (1 d before championship)	51.2 ± 1.5	49.5 ± 1.2	not reported	2 × 2 × 2 × 6 MANOVA: overall POMS procedure main effect $p < 0.01$; procedure × sex × group interaction $p < 0.05$ flagged (symbol a) only for diet-group cells (this cell not flagged)	not reported	No	Null	Diet group: −4 MJ/day for 21 days + plastic-suit sweating during the last 6 days (≈4 weeks in total). MANOVA with Tukey post hoc; effects not reported separately per sex × group cell except via table symbols. Dispersion values (e.g., ±0.4 on T-score-type values) are implausibly small for an SD and may be SEs; the article labels them 's'. Control-group changes of similar size (e.g., anger in control females 45.5→55.6) were not flagged as significant.
Koral & Dosseville, 2009 [33]	Control group – females (n = 5)	POMS – Fatigue	T1 (4 wk before championship) vs T2 (1 d before championship)	42.7 ± 0.6	49.6 ± 1.5	not reported	2 × 2 × 2 × 6 MANOVA: overall POMS procedure main effect $p < 0.01$; procedure × sex × group interaction $p < 0.05$ flagged (symbol a) only for diet-group cells (this cell not flagged)	not reported	No	Null	Diet group: −4 MJ/day for 21 days + plastic-suit sweating during the last 6 days (≈4 weeks in total). MANOVA with Tukey post hoc; effects not reported separately per sex × group cell except via table symbols. Dispersion values (e.g., ±0.4 on T-score-type values) are implausibly small for an SD and may be SEs; the article labels them 's'. Control-group changes of similar size (e.g., anger in control females 45.5→55.6) were not flagged as significant.

Study (first author, year) [ref]	Group / condition	Outcome	Time point	Pre value (mean ± SD)	Post value (mean ± SD)	Change (absolute and/or %)	Test statistic and p value	Effect size (95% CI if reported)	Statistically significant (Yes / No)	Direction (Favourable / Unfavourable / Null)	Notes
Koral & Dosseville, 2009 [33]	Diet group – males (n = 5)	POMS – Confusion	T1 (4 wk before championship) vs T2 (1 d before championship)	38.6 ± 1.1	49.6 ± 1.3	not reported	2 × 2 × 2 × 6 MANOVA: overall POMS procedure main effect p < 0.01; procedure × sex × group interaction p < 0.05 flagged (symbol a) only for diet-group cells	not reported	Yes (procedure × sex × group interaction, p < 0.05)	Unfavourable	Diet group: −4 MJ/day for 21 days + plastic-suit sweating during the last 6 days (≈4 weeks in total). MANOVA with Tukey post hoc; effects not reported separately per sex × group cell except via table symbols. Dispersion values (e.g., ±0.4 on T-score-type values) are implausibly small for an SD and may be SEs; the article labels them 's'. Abstract gives within-group differences with signs rendered as '7' (e.g., confusion '714.6 ± 7.9', tension '710.1 ± 12.5', vigour '11.3 ± 8.5'), apparently expressed as T1 − T2.

Study (first author, year) [ref]	Group / condition	Outcome	Time point	Pre value (mean ± SD)	Post value (mean ± SD)	Change (absolute and/or %)	Test statistic and p value	Effect size (95% CI if reported)	Statistically significant (Yes / No)	Direction (Favourable / Unfavourable / Null)	Notes
Koral & Dosseville, 2009 [33]	Diet group – females (n = 5)	POMS – Confusion	T1 (4 wk before championship) vs T2 (1 d before championship)	34.1 ± 1.9	52.2 ± 2.7	not reported	2 × 2 × 2 × 6 MANOVA: overall POMS procedure main effect $p < 0.01$; procedure × sex × group interaction $p < 0.05$ flagged (symbol a) only for diet-group cells	not reported	Yes (procedure × sex × group interaction, $p < 0.05$)	Unfavourable	Diet group: −4 MJ/day for 21 days + plastic-suit sweating during the last 6 days (≈4 weeks in total). MANOVA with Tukey post hoc; effects not reported separately per sex × group cell except via table symbols. Dispersion values (e.g., ±0.4 on T-score-type values) are implausibly small for an SD and may be SEs; the article labels them 's'. Abstract gives within-group differences with signs rendered as '7' (e.g., confusion '714.6 ± 7.9', tension '710.1 ± 12.5', vigour '11.3 ± 8.5'), apparently expressed as T1 − T2.

Study (first author, year) [ref]	Group / condition	Outcome	Time point	Pre value (mean ± SD)	Post value (mean ± SD)	Change (absolute and/or %)	Test statistic and p value	Effect size (95% CI if reported)	Statistically significant (Yes / No)	Direction (Favourable / Unfavourable / Null)	Notes
Koral & Dosseville, 2009 [33]	Control group – males (n = 5)	POMS – Confusion	T1 (4 wk before championship) vs T2 (1 d before championship)	38.4 ± 2.4	47.2 ± 1.1	not reported	2 × 2 × 2 × 6 MANOVA: overall POMS procedure main effect p < 0.01; procedure × sex × group interaction p < 0.05 flagged (symbol a) only for diet-group cells (this cell not flagged)	not reported	No	Null	Diet group: −4 MJ/day for 21 days + plastic-suit sweating during the last 6 days (≈4 weeks in total). MANOVA with Tukey post hoc; effects not reported separately per sex × group cell except via table symbols. Dispersion values (e.g., ±0.4 on T-score-type values) are implausibly small for an SD and may be SEs; the article labels them 's'. Control-group changes of similar size (e.g., anger in control females 45.5→55.6) were not flagged as significant.
Koral & Dosseville, 2009 [33]	Control group – females (n = 5)	POMS – Confusion	T1 (4 wk before championship) vs T2 (1 d before championship)	40.5 ± 2.3	43.1 ± 0.8 [printed '08']	not reported	2 × 2 × 2 × 6 MANOVA: overall POMS procedure main effect p < 0.01; procedure × sex × group interaction p < 0.05 flagged (symbol a) only for diet-group cells (this cell not flagged)	not reported	No	Null	Diet group: −4 MJ/day for 21 days + plastic-suit sweating during the last 6 days (≈4 weeks in total). MANOVA with Tukey post hoc; effects not reported separately per sex × group cell except via table symbols. Dispersion values (e.g., ±0.4 on T-score-type values) are implausibly small for an SD and may be SEs; the article labels them 's'. Control-group changes of similar size (e.g., anger in control females 45.5→55.6) were not flagged as significant.

Study (first author, year) [ref]	Group / condition	Outcome	Time point	Pre value (mean ± SD)	Post value (mean ± SD)	Change (absolute and/or %)	Test statistic and p value	Effect size (95% CI if reported)	Statistically significant (Yes / No)	Direction (Favourable / Unfavourable / Null)	Notes
Miranda et al., 2021 [34]	Single group of wrestlers (gradual weight loss over ~20 d; n = 8: 5 M, 3 F)	Concentric knee extension peak torque, 60°/s (Nm)	Baseline (weight maintenance, 20 d before starting weight loss) vs post (precompetitive period, 3 d before national competition)	Figure only	Figure only	not reported	Paired t-test, p = 0.086	Cohen's ES shown in Figure 1 (assignment ambiguous; see notes)	No	Null	No control group; single-group pre–post design; paired t-tests (p ≤ 0.05). Weight loss self-selected (−2.6 kg, −3.7% BM). Peak torque values shown only in Figure 1 (bar chart; not extracted). Effect sizes printed in Figure 1 (0.22, 0.15, 0.10, 0.17, 0.25, 0.05, 0.09, 0.01) cannot be unambiguously assigned to each movement from the text layer. Dominant limb only.
Miranda et al., 2021 [34]	Single group of wrestlers (gradual weight loss over ~20 d; n = 8: 5 M, 3 F)	Concentric knee extension peak torque, 180°/s (Nm)	Baseline (weight maintenance, 20 d before starting weight loss) vs post (precompetitive period, 3 d before national competition)	Figure only	Figure only	not reported	Paired t-test, p = 0.630	Cohen's ES shown in Figure 1 (assignment ambiguous; see notes)	No	Null	No control group; single-group pre–post design; paired t-tests (p ≤ 0.05). Weight loss self-selected (−2.6 kg, −3.7% BM). Peak torque values shown only in Figure 1 (bar chart; not extracted). Effect sizes printed in Figure 1 (0.22, 0.15, 0.10, 0.17, 0.25, 0.05, 0.09, 0.01) cannot be unambiguously assigned to each movement from the text layer. Dominant limb only.

Study (first author, year) [ref]	Group / condition	Outcome	Time point	Pre value (mean ± SD)	Post value (mean ± SD)	Change (absolute and/or %)	Test statistic and p value	Effect size (95% CI if reported)	Statistically significant (Yes / No)	Direction (Favourable / Unfavourable / Null)	Notes
Miranda et al., 2021 [34]	Single group of wrestlers (gradual weight loss over ~20 d; n = 8: 5 M, 3 F)	Concentric knee flexion peak torque, 60°/s (Nm)	Baseline (weight maintenance, 20 d before starting weight loss) vs post (precompetitive period, 3 d before national competition)	Figure only	Figure only	not reported	Paired t-test, p = 0.310	Cohen's ES shown in Figure 1 (assignment ambiguous; see notes)	No	Null	No control group; single-group pre–post design; paired t-tests (p ≤ 0.05). Weight loss self-selected (−2.6 kg, −3.7% BM). Peak torque values shown only in Figure 1 (bar chart; not extracted). Effect sizes printed in Figure 1 (0.22, 0.15, 0.10, 0.17, 0.25, 0.05, 0.09, 0.01) cannot be unambiguously assigned to each movement from the text layer. Dominant limb only.
Miranda et al., 2021 [34]	Single group of wrestlers (gradual weight loss over ~20 d; n = 8: 5 M, 3 F)	Concentric knee flexion peak torque, 180°/s (Nm)	Baseline (weight maintenance, 20 d before starting weight loss) vs post (precompetitive period, 3 d before national competition)	Figure only	Figure only	not reported	Paired t-test, p = 0.239	Cohen's ES shown in Figure 1 (assignment ambiguous; see notes)	No	Null	No control group; single-group pre–post design; paired t-tests (p ≤ 0.05). Weight loss self-selected (−2.6 kg, −3.7% BM). Peak torque values shown only in Figure 1 (bar chart; not extracted). Effect sizes printed in Figure 1 (0.22, 0.15, 0.10, 0.17, 0.25, 0.05, 0.09, 0.01) cannot be unambiguously assigned to each movement from the text layer. Dominant limb only.

Study (first author, year) [ref]	Group / condition	Outcome	Time point	Pre value (mean ± SD)	Post value (mean ± SD)	Change (absolute and/or %)	Test statistic and p value	Effect size (95% CI if reported)	Statistically significant (Yes / No)	Direction (Favourable / Unfavourable / Null)	Notes
Miranda et al., 2021 [34]	Single group of wrestlers (gradual weight loss over ~20 d; n = 8: 5 M, 3 F)	Concentric shoulder internal rotation peak torque, 60°/s (Nm)	Baseline (weight maintenance, 20 d before starting weight loss) vs post (precompetitive period, 3 d before national competition)	Figure only	Figure only	not reported	Paired t-test, p = 0.825	Cohen's ES shown in Figure 1 (assignment ambiguous; see notes)	No	Null	No control group; single-group pre–post design; paired t-tests (p ≤ 0.05). Weight loss self-selected (−2.6 kg, −3.7% BM). Peak torque values shown only in Figure 1 (bar chart; not extracted). Effect sizes printed in Figure 1 (0.22, 0.15, 0.10, 0.17, 0.25, 0.05, 0.09, 0.01) cannot be unambiguously assigned to each movement from the text layer. Dominant limb only.
Miranda et al., 2021 [34]	Single group of wrestlers (gradual weight loss over ~20 d; n = 8: 5 M, 3 F)	Concentric shoulder internal rotation peak torque, 180°/s (Nm)	Baseline (weight maintenance, 20 d before starting weight loss) vs post (precompetitive period, 3 d before national competition)	Figure only	Figure only	not reported	Paired t-test, p = 0.245	Cohen's ES shown in Figure 1 (assignment ambiguous; see notes)	No	Null	No control group; single-group pre–post design; paired t-tests (p ≤ 0.05). Weight loss self-selected (−2.6 kg, −3.7% BM). Peak torque values shown only in Figure 1 (bar chart; not extracted). Effect sizes printed in Figure 1 (0.22, 0.15, 0.10, 0.17, 0.25, 0.05, 0.09, 0.01) cannot be unambiguously assigned to each movement from the text layer. Dominant limb only.

Study (first author, year) [ref]	Group / condition	Outcome	Time point	Pre value (mean ± SD)	Post value (mean ± SD)	Change (absolute and/or %)	Test statistic and p value	Effect size (95% CI if reported)	Statistically significant (Yes / No)	Direction (Favourable / Unfavourable / Null)	Notes
Miranda et al., 2021 [34]	Single group of wrestlers (gradual weight loss over ~20 d; n = 8: 5 M, 3 F)	Concentric shoulder external rotation peak torque, 60°/s (Nm)	Baseline (weight maintenance, 20 d before starting weight loss) vs post (precompetitive period, 3 d before national competition)	Figure only	Figure only	not reported	Paired t-test, p = 0.149	Cohen's ES shown in Figure 1 (assignment ambiguous; see notes)	No	Null	No control group; single-group pre–post design; paired t-tests (p ≤ 0.05). Weight loss self-selected (−2.6 kg, −3.7% BM). Peak torque values shown only in Figure 1 (bar chart; not extracted). Effect sizes printed in Figure 1 (0.22, 0.15, 0.10, 0.17, 0.25, 0.05, 0.09, 0.01) cannot be unambiguously assigned to each movement from the text layer. Dominant limb only.
Miranda et al., 2021 [34]	Single group of wrestlers (gradual weight loss over ~20 d; n = 8: 5 M, 3 F)	Concentric shoulder external rotation peak torque, 180°/s (Nm)	Baseline (weight maintenance, 20 d before starting weight loss) vs post (precompetitive period, 3 d before national competition)	Figure only	Figure only	not reported	Paired t-test, p = 0.163	Cohen's ES shown in Figure 1 (assignment ambiguous; see notes)	No	Null	No control group; single-group pre–post design; paired t-tests (p ≤ 0.05). Weight loss self-selected (−2.6 kg, −3.7% BM). Peak torque values shown only in Figure 1 (bar chart; not extracted). Effect sizes printed in Figure 1 (0.22, 0.15, 0.10, 0.17, 0.25, 0.05, 0.09, 0.01) cannot be unambiguously assigned to each movement from the text layer. Dominant limb only.
Yoshioka et al., 2006 [35]	WR group – males (weight reduction ~3.4% BM; n = 22)	POMS – Anger	Pre (20 d before competition) vs post (1 d before competition)	6.2 ± 4.5	6.6 ± 6.8	not reported	Three-way ANOVA; within-group pre vs post not significant (p NR)	not reported	No	Null	POMS raw scores (mean ± SD); three-way ANOVA (group × sex × time). Non-randomised allocation (need for weight reduction).

Study (first author, year) [ref]	Group / condition	Outcome	Time point	Pre value (mean ± SD)	Post value (mean ± SD)	Change (absolute and/or %)	Test statistic and p value	Effect size (95% CI if reported)	Statistically significant (Yes / No)	Direction (Favourable / Unfavourable / Null)	Notes
Yoshioka et al., 2006 [35]	WR group – females (weight reduction ~4.9% BM; n = 8)	POMS – Anger	Pre (20 d before competition) vs post (1 d before competition)	11.0 ± 7.5	8.0 ± 5.4	not reported	Three-way ANOVA; within-group pre vs post not significant (p NR)	not reported	No	Null	POMS raw scores (mean ± SD); three-way ANOVA (group × sex × time). Non-randomised allocation (need for weight reduction). Authors describe a non-significant 'tendency to decrease' in WR females.
Yoshioka et al., 2006 [35]	Non-WR group – males (n = 5)	POMS – Anger	Pre (20 d before competition) vs post (1 d before competition)	5.2 ± 4.2	9.6 ± 5.6	not reported	Three-way ANOVA; within-group pre vs post not significant (p NR)	not reported	No	Null	POMS raw scores (mean ± SD); three-way ANOVA (group × sex × time). Non-randomised allocation (need for weight reduction).
Yoshioka et al., 2006 [35]	Non-WR group – females (n = 8)	POMS – Anger	Pre (20 d before competition) vs post (1 d before competition)	5.1 ± 5.3	5.8 ± 7.2	not reported	Three-way ANOVA; within-group pre vs post not significant (p NR)	not reported	No	Null	POMS raw scores (mean ± SD); three-way ANOVA (group × sex × time). Non-randomised allocation (need for weight reduction).
Yoshioka et al., 2006 [35]	WR group – males (weight reduction ~3.4% BM; n = 22)	POMS – Confusion	Pre (20 d before competition) vs post (1 d before competition)	7.3 ± 2.4	8.1 ± 4.7	not reported	Three-way ANOVA; within-group pre vs post not significant (p NR)	not reported	No	Null	POMS raw scores (mean ± SD); three-way ANOVA (group × sex × time). Non-randomised allocation (need for weight reduction).

Study (first author, year) [ref]	Group / condition	Outcome	Time point	Pre value (mean ± SD)	Post value (mean ± SD)	Change (absolute and/or %)	Test statistic and p value	Effect size (95% CI if reported)	Statistically significant (Yes / No)	Direction (Favourable / Unfavourable / Null)	Notes
Yoshioka et al., 2006 [35]	WR group – females (weight reduction ~4.9% BM; n = 8)	POMS – Confusion	Pre (20 d before competition) vs post (1 d before competition)	12.4 ± 7.0	10.1 ± 8.6	not reported	Three-way ANOVA; within-group pre vs post not significant (p NR)	not reported	No	Null	POMS raw scores (mean ± SD); three-way ANOVA (group × sex × time). Non-randomised allocation (need for weight reduction). † Pre-value significantly higher in WR females than WR males ($p < 0.05$). Authors describe a non-significant 'tendency to decrease' in WR females.
Yoshioka et al., 2006 [35]	Non-WR group – males (n = 5)	POMS – Confusion	Pre (20 d before competition) vs post (1 d before competition)	8.6 ± 5.3	9.8 ± 4.0	not reported	Three-way ANOVA; within-group pre vs post not significant (p NR)	not reported	No	Null	POMS raw scores (mean ± SD); three-way ANOVA (group × sex × time). Non-randomised allocation (need for weight reduction).
Yoshioka et al., 2006 [35]	Non-WR group – females (n = 8)	POMS – Confusion	Pre (20 d before competition) vs post (1 d before competition)	7.9 ± 3.3	8.1 ± 3.8	not reported	Three-way ANOVA; within-group pre vs post not significant (p NR)	not reported	No	Null	POMS raw scores (mean ± SD); three-way ANOVA (group × sex × time). Non-randomised allocation (need for weight reduction).
Yoshioka et al., 2006 [35]	WR group – males (weight reduction ~3.4% BM; n = 22)	POMS – Depression	Pre (20 d before competition) vs post (1 d before competition)	5.6 ± 5.1	8.1 ± 9.7	not reported	Three-way ANOVA; within-group pre vs post not significant (p NR)	not reported	No	Null	POMS raw scores (mean ± SD); three-way ANOVA (group × sex × time). Non-randomised allocation (need for weight reduction).

Study (first author, year) [ref]	Group / condition	Outcome	Time point	Pre value (mean ± SD)	Post value (mean ± SD)	Change (absolute and/or %)	Test statistic and p value	Effect size (95% CI if reported)	Statistically significant (Yes / No)	Direction (Favourable / Unfavourable / Null)	Notes
Yoshioka et al., 2006 [35]	WR group – females (weight reduction ~4.9% BM; n = 8)	POMS – Depression	Pre (20 d before competition) vs post (1 d before competition)	17.9 ± 17.3	13.6 ± 11.8	not reported	Three-way ANOVA; within-group pre vs post not significant (p NR)	not reported	No	Null	POMS raw scores (mean ± SD); three-way ANOVA (group × sex × time). Non-randomised allocation (need for weight reduction). † Pre-value significantly higher in WR females than WR males ($p < 0.05$). Authors describe a non-significant 'tendency to decrease' in WR females.
Yoshioka et al., 2006 [35]	Non-WR group – males (n = 5)	POMS – Depression	Pre (20 d before competition) vs post (1 d before competition)	9.8 ± 7.5	12.4 ± 9.4	not reported	Three-way ANOVA; within-group pre vs post not significant (p NR)	not reported	No	Null	POMS raw scores (mean ± SD); three-way ANOVA (group × sex × time). Non-randomised allocation (need for weight reduction).
Yoshioka et al., 2006 [35]	Non-WR group – females (n = 8)	POMS – Depression	Pre (20 d before competition) vs post (1 d before competition)	7.8 ± 6.1	7.3 ± 10.7	not reported	Three-way ANOVA; within-group pre vs post not significant (p NR)	not reported	No	Null	POMS raw scores (mean ± SD); three-way ANOVA (group × sex × time). Non-randomised allocation (need for weight reduction).
Yoshioka et al., 2006 [35]	WR group – males (weight reduction ~3.4% BM; n = 22)	POMS – Fatigue	Pre (20 d before competition) vs post (1 d before competition)	5.7 ± 4.1	8.2 ± 6.0	not reported	Three-way ANOVA; within-group pre vs post $p < 0.01$	not reported	Yes (within-group)	Unfavourable	POMS raw scores (mean ± SD); three-way ANOVA (group × sex × time). Non-randomised allocation (need for weight reduction).

Study (first author, year) [ref]	Group / condition	Outcome	Time point	Pre value (mean ± SD)	Post value (mean ± SD)	Change (absolute and/or %)	Test statistic and p value	Effect size (95% CI if reported)	Statistically significant (Yes / No)	Direction (Favourable / Unfavourable / Null)	Notes
Yoshioka et al., 2006 [35]	WR group – females (weight reduction ~4.9% BM; n = 8)	POMS – Fatigue	Pre (20 d before competition) vs post (1 d before competition)	8.8 ± 6.8	10.4 ± 10.4	not reported	Three-way ANOVA; within-group pre vs post not significant (p NR)	not reported	No	Null	POMS raw scores (mean ± SD); three-way ANOVA (group × sex × time). Non-randomised allocation (need for weight reduction).
Yoshioka et al., 2006 [35]	Non-WR group – males (n = 5)	POMS – Fatigue	Pre (20 d before competition) vs post (1 d before competition)	7.0 ± 3.1	6.6 ± 1.3	not reported	Three-way ANOVA; within-group pre vs post not significant (p NR)	not reported	No	Null	POMS raw scores (mean ± SD); three-way ANOVA (group × sex × time). Non-randomised allocation (need for weight reduction).
Yoshioka et al., 2006 [35]	Non-WR group – females (n = 8)	POMS – Fatigue	Pre (20 d before competition) vs post (1 d before competition)	8.0 ± 3.8	9.3 ± 8.4	not reported	Three-way ANOVA; within-group pre vs post not significant (p NR)	not reported	No	Null	POMS raw scores (mean ± SD); three-way ANOVA (group × sex × time). Non-randomised allocation (need for weight reduction).
Yoshioka et al., 2006 [35]	WR group – males (weight reduction ~3.4% BM; n = 22)	POMS – Tension	Pre (20 d before competition) vs post (1 d before competition)	9.5 ± 3.9	10.7 ± 5.7	not reported	Three-way ANOVA; within-group pre vs post $p < 0.05$	not reported	Yes (within-group)	Unfavourable	POMS raw scores (mean ± SD); three-way ANOVA (group × sex × time). Non-randomised allocation (need for weight reduction).
Yoshioka et al., 2006 [35]	WR group – females (weight reduction ~4.9% BM; n = 8)	POMS – Tension	Pre (20 d before competition) vs post (1 d before competition)	13.8 ± 6.8	10.6 ± 6.6	not reported	Three-way ANOVA; within-group pre vs post not significant (p NR)	not reported	No	Null	POMS raw scores (mean ± SD); three-way ANOVA (group × sex × time). Non-randomised allocation (need for weight reduction). Authors describe a non-significant 'tendency to decrease' in WR females.

Study (first author, year) [ref]	Group / condition	Outcome	Time point	Pre value (mean ± SD)	Post value (mean ± SD)	Change (absolute and/or %)	Test statistic and p value	Effect size (95% CI if reported)	Statistically significant (Yes / No)	Direction (Favourable / Unfavourable / Null)	Notes
Yoshioka et al., 2006 [35]	Non-WR group – males (n = 5)	POMS – Tension	Pre (20 d before competition) vs post (1 d before competition)	13.4 ± 3.4	10.8 ± 3.6	not reported	Three-way ANOVA; within-group pre vs post not significant (p NR)	not reported	No	Null	POMS raw scores (mean ± SD); three-way ANOVA (group × sex × time). Non-randomised allocation (need for weight reduction).
Yoshioka et al., 2006 [35]	Non-WR group – females (n = 8)	POMS – Tension	Pre (20 d before competition) vs post (1 d before competition)	9.8 ± 3.4	9.3 ± 4.4	not reported	Three-way ANOVA; within-group pre vs post not significant (p NR)	not reported	No	Null	POMS raw scores (mean ± SD); three-way ANOVA (group × sex × time). Non-randomised allocation (need for weight reduction).
Yoshioka et al., 2006 [35]	WR group – males (weight reduction ~3.4% BM; n = 22)	POMS – Vigor	Pre (20 d before competition) vs post (1 d before competition)	9.6 ± 4.7	7.5 ± 4.9	not reported	Three-way ANOVA; within-group pre vs post $p < 0.05$	not reported	Yes (within-group)	Unfavourable	POMS raw scores (mean ± SD); three-way ANOVA (group × sex × time). Non-randomised allocation (need for weight reduction). Text/table discrepancy: Results and Discussion state that Vigor 'significantly increased' in WR males, whereas Table 5 shows a decrease (9.6 → 7.5, * $p < 0.05$).
Yoshioka et al., 2006 [35]	WR group – females (weight reduction ~4.9% BM; n = 8)	POMS – Vigor	Pre (20 d before competition) vs post (1 d before competition)	12.9 ± 4.2	10.5 ± 8.9	not reported	Three-way ANOVA; within-group pre vs post not significant (p NR)	not reported	No	Null	POMS raw scores (mean ± SD); three-way ANOVA (group × sex × time). Non-randomised allocation (need for weight reduction).

Study (first author, year) [ref]	Group / condition	Outcome	Time point	Pre value (mean ± SD)	Post value (mean ± SD)	Change (absolute and/or %)	Test statistic and p value	Effect size (95% CI if reported)	Statistically significant (Yes / No)	Direction (Favourable / Unfavourable / Null)	Notes
Yoshioka et al., 2006 [35]	Non-WR group – males (n = 5)	POMS – Vigor	Pre (20 d before competition) vs post (1 d before competition)	11.0 ± 1.6	11.2 ± 5.0	not reported	Three-way ANOVA; within-group pre vs post not significant (p NR)	not reported	No	Null	POMS raw scores (mean ± SD); three-way ANOVA (group × sex × time). Non-randomised allocation (need for weight reduction).
Yoshioka et al., 2006 [35]	Non-WR group – females (n = 8)	POMS – Vigor	Pre (20 d before competition) vs post (1 d before competition)	11.0 ± 9.5	10.0 ± 9.6	not reported	Three-way ANOVA; within-group pre vs post not significant (p NR)	not reported	No	Null	POMS raw scores (mean ± SD); three-way ANOVA (group × sex × time). Non-randomised allocation (need for weight reduction).
Yoshioka et al., 2006 [35]	WR group – males (weight reduction ~3.4% BM; n = 22)	POMS – Total mood disturbance (TMD = T + D + A + F + C + 100 − V)	Pre (20 d before competition) vs post (1 d before competition)	122.6 ± 14.6	134.1 ± 32.3	not reported	Three-way ANOVA; within-group pre vs post $p < 0.05$	not reported	Yes (within-group)	Unfavourable	POMS raw scores (mean ± SD); three-way ANOVA (group × sex × time). Non-randomised allocation (need for weight reduction).
Yoshioka et al., 2006 [35]	WR group – females (weight reduction ~4.9% BM; n = 8)	POMS – Total mood disturbance (TMD = T + D + A + F + C + 100 − V)	Pre (20 d before competition) vs post (1 d before competition)	150.8 ± 44.7	142.2 ± 46.3	not reported	Three-way ANOVA; within-group pre vs post not significant (p NR)	not reported	No	Null	POMS raw scores (mean ± SD); three-way ANOVA (group × sex × time). Non-randomised allocation (need for weight reduction). † Pre-value significantly higher in WR females than WR males ($p < 0.05$). Authors describe a non-significant 'tendency to decrease' in WR females.

Study (first author, year) [ref]	Group / condition	Outcome	Time point	Pre value (mean ± SD)	Post value (mean ± SD)	Change (absolute and/or %)	Test statistic and p value	Effect size (95% CI if reported)	Statistically significant (Yes / No)	Direction (Favourable / Unfavourable / Null)	Notes
Yoshioka et al., 2006 [35]	Non-WR group – males (n = 5)	POMS – Total mood disturbance (TMD = T + D + A + F + C + 100 − V)	Pre (20 d before competition) vs post (1 d before competition)	130.8 ± 20.0	131.8 ± 13.3	not reported	Three-way ANOVA; within-group pre vs post not significant (p NR)	not reported	No	Null	POMS raw scores (mean ± SD); three-way ANOVA (group × sex × time). Non-randomised allocation (need for weight reduction).
Yoshioka et al., 2006 [35]	Non-WR group – females (n = 8)	POMS – Total mood disturbance (TMD = T + D + A + F + C + 100 − V)	Pre (20 d before competition) vs post (1 d before competition)	127.5 ± 25.0	129.6 ± 36.7	not reported	Three-way ANOVA; within-group pre vs post not significant (p NR)	not reported	No	Null	POMS raw scores (mean ± SD); three-way ANOVA (group × sex × time). Non-randomised allocation (need for weight reduction).
Yoshioka et al., 2006 [35]	WR groups (males and females)	Competition results	Tournament	not reported	WR males: 2 champions, one 2nd and one 3rd place; WR females: 1 champion	not reported	No significant relationship between results and weight-reduction rate or POMS changes (statistics NR)	not reported	not tested	not reported	POMS raw scores (mean ± SD); three-way ANOVA (group × sex × time). Non-randomised allocation (need for weight reduction).
Artioli et al., 2010 [36]	Weight loss group (weight-cyclers; ~5% BM in 5–7 d, then 4 h refeeding; n = 7)	Judo combat – total effort time (s)	Pre-intervention vs post-intervention (5–7 d apart; WL tested after 4-h recovery)	229 ± 17	228 ± 24	not reported	No group, time or interaction effects (F and p NR)	not reported	No	Null	Non-randomised allocation (weight-cyclers vs non-weight-cyclers). Mixed-model two-way ANOVA (time × group) with Tukey post hoc. Achieved WL 4.8 ± 1.1% BM; 51 ± 13% of lost mass regained during recovery. No familiarisation trial (authors attribute time effects to learning). Table IV. Time units not stated in the table (assumed seconds).

Study (first author, year) [ref]	Group / condition	Outcome	Time point	Pre value (mean ± SD)	Post value (mean ± SD)	Change (absolute and/or %)	Test statistic and p value	Effect size (95% CI if reported)	Statistically significant (Yes / No)	Direction (Favourable / Unfavourable / Null)	Notes
Artioli et al., 2010 [36]	Control group (non-weight-cyclers; weight stable; n = 7)	Judo combat – total effort time (s)	Pre-intervention vs post-intervention (5–7 d apart; WL tested after 4-h recovery)	237 ± 11	239 ± 8	not reported	No group, time or interaction effects (F and p NR)	not reported	No	Null	—
Artioli et al., 2010 [36]	Weight loss group (weight-cyclers; ~5% BM in 5–7 d, then 4 h refeeding; n = 7)	Judo combat – total recovery time (s)	Pre-intervention vs post-intervention (5–7 d apart; WL tested after 4-h recovery)	71 ± 17	72 ± 24	not reported	No group, time or interaction effects (F and p NR)	not reported	No	Null	Non-randomised allocation (weight-cyclers vs non-weight-cyclers). Mixed-model two-way ANOVA (time × group) with Tukey post hoc. Achieved WL 4.8 ± 1.1% BM; 51 ± 13% of lost mass regained during recovery. No familiarisation trial (authors attribute time effects to learning). Table IV. Time units not stated in the table (assumed seconds).
Artioli et al., 2010 [36]	Control group (non-weight-cyclers; weight stable; n = 7)	Judo combat – total recovery time (s)	Pre-intervention vs post-intervention (5–7 d apart; WL tested after 4-h recovery)	63 ± 11	61 ± 8	not reported	No group, time or interaction effects (F and p NR)	not reported	No	Null	—

Study (first author, year) [ref]	Group / condition	Outcome	Time point	Pre value (mean ± SD)	Post value (mean ± SD)	Change (absolute and/or %)	Test statistic and p value	Effect size (95% CI if reported)	Statistically significant (Yes / No)	Direction (Favourable / Unfavourable / Null)	Notes
Artioli et al., 2010 [36]	Weight loss group (weight-cyclers; ~5% BM in 5–7 d, then 4 h refeeding; n = 7)	Judo combat – total standing combat time (s)	Pre-intervention vs post-intervention (5–7 d apart; WL tested after 4-h recovery)	199 ± 9	191 ± 18	not reported	No group, time or interaction effects (F and p NR)	not reported	No	Null	Non-randomised allocation (weight-cyclers vs non-weight-cyclers). Mixed-model two-way ANOVA (time × group) with Tukey post hoc. Achieved WL 4.8 ± 1.1% BM; 51 ± 13% of lost mass regained during recovery. No familiarisation trial (authors attribute time effects to learning). Table IV. Time units not stated in the table (assumed seconds).
Artioli et al., 2010 [36]	Control group (non-weight-cyclers; weight stable; n = 7)	Judo combat – total standing combat time (s)	Pre-intervention vs post-intervention (5–7 d apart; WL tested after 4-h recovery)	191 ± 19	204 ± 22	not reported	No group, time or interaction effects (F and p NR)	not reported	No	Null	—
Artioli et al., 2010 [36]	Weight loss group (weight-cyclers; ~5% BM in 5–7 d, then 4 h refeeding; n = 7)	Judo combat – total ground work time (s)	Pre-intervention vs post-intervention (5–7 d apart; WL tested after 4-h recovery)	30 ± 14	38 ± 19	not reported	No group, time or interaction effects (F and p NR)	not reported	No	Null	Non-randomised allocation (weight-cyclers vs non-weight-cyclers). Mixed-model two-way ANOVA (time × group) with Tukey post hoc. Achieved WL 4.8 ± 1.1% BM; 51 ± 13% of lost mass regained during recovery. No familiarisation trial (authors attribute time effects to learning). Table IV. Time units not stated in the table (assumed seconds).

Study (first author, year) [ref]	Group / condition	Outcome	Time point	Pre value (mean ± SD)	Post value (mean ± SD)	Change (absolute and/or %)	Test statistic and p value	Effect size (95% CI if reported)	Statistically significant (Yes / No)	Direction (Favourable / Unfavourable / Null)	Notes
Artioli et al., 2010 [36]	Control group (non-weight-cyclers; weight stable; n = 7)	Judo combat – total ground work time (s)	Pre-intervention vs post-intervention (5–7 d apart; WL tested after 4-h recovery)	47 ± 17	35 ± 19	not reported	No group, time or interaction effects (F and p NR)	not reported	No	Null	—
Artioli et al., 2010 [36]	Weight loss group (weight-cyclers; ~5% BM in 5–7 d, then 4 h refeeding; n = 7)	Judo combat – number of attacks (n)	Pre-intervention vs post-intervention (5–7 d apart; WL tested after 4-h recovery)	Figure only	Figure only	not reported	No significant differences (p NR)	not reported	No	Null	Non-randomised allocation (weight-cyclers vs non-weight-cyclers). Mixed-model two-way ANOVA (time × group) with Tukey post hoc. Achieved WL 4.8 ± 1.1% BM; 51 ± 13% of lost mass regained during recovery. No familiarisation trial (authors attribute time effects to learning). Data only in Figure 2.
Artioli et al., 2010 [36]	Weight loss group (weight-cyclers; ~5% BM in 5–7 d, then 4 h refeeding; n = 7)	Uchi-komi exercise (3 bouts: 10 s, 20 s, 30 s) – repetitions	Pre-intervention vs post-intervention (5–7 d apart; WL tested after 4-h recovery)	not reported	not reported	not reported	Results not reported in the article	not reported	not reported	not reported	Non-randomised allocation (weight-cyclers vs non-weight-cyclers). Mixed-model two-way ANOVA (time × group) with Tukey post hoc. Achieved WL 4.8 ± 1.1% BM; 51 ± 13% of lost mass regained during recovery. No familiarisation trial (authors attribute time effects to learning). Uchi-komi was part of the protocol, but no uchi-komi performance data or statistics are reported in Results.

Study (first author, year) [ref]	Group / condition	Outcome	Time point	Pre value (mean ± SD)	Post value (mean ± SD)	Change (absolute and/or %)	Test statistic and p value	Effect size (95% CI if reported)	Statistically significant (Yes / No)	Direction (Favourable / Unfavourable / Null)	Notes
Artioli et al., 2010 [36]	Control group (non-weight-cyclers; weight stable; n = 7)	Judo combat – number of attacks (n)	Pre-intervention vs post-intervention (5–7 d apart; WL tested after 4-h recovery)	Figure only	Figure only	not reported	No significant differences (p NR)	not reported	No	Null	Non-randomised allocation (weight-cyclers vs non-weight-cyclers). Mixed-model two-way ANOVA (time × group) with Tukey post hoc. Achieved WL 4.8 ± 1.1% BM; 51 ± 13% of lost mass regained during recovery. No familiarisation trial (authors attribute time effects to learning). Data only in Figure 2.
Artioli et al., 2010 [36]	Control group (non-weight-cyclers; weight stable; n = 7)	Uchi-komi exercise (3 bouts: 10 s, 20 s, 30 s) – repetitions	Pre-intervention vs post-intervention (5–7 d apart; WL tested after 4-h recovery)	not reported	not reported	not reported	Results not reported in the article	not reported	not reported	not reported	Non-randomised allocation (weight-cyclers vs non-weight-cyclers). Mixed-model two-way ANOVA (time × group) with Tukey post hoc. Achieved WL 4.8 ± 1.1% BM; 51 ± 13% of lost mass regained during recovery. No familiarisation trial (authors attribute time effects to learning). Uchi-komi was part of the protocol, but no uchi-komi performance data or statistics are reported in Results.

Study (first author, year) [ref]	Group / condition	Outcome	Time point	Pre value (mean ± SD)	Post value (mean ± SD)	Change (absolute and/or %)	Test statistic and p value	Effect size (95% CI if reported)	Statistically significant (Yes / No)	Direction (Favourable / Unfavourable / Null)	Notes
Artioli et al., 2010 [36]	Weight loss group (weight-cyclers; ~5% BM in 5–7 d, then 4 h refeeding; n = 7)	Upper-body Wingate (3 × 30 s) – absolute mean power (W)	Pre-intervention vs post-intervention (5–7 d apart; WL tested after 4-h recovery)	Figure only	Figure only	'Discrete improvement' post vs pre (magnitude NR)	Main effect of time $p < 0.01$ (pre vs post, both groups); no group or interaction effects (F and p NR)	not reported	Yes (time main effect only; no interaction)	Favourable (time effect in both groups; attributed to learning)	Non-randomised allocation (weight-cyclers vs non-weight-cyclers). Mixed-model two-way ANOVA (time × group) with Tukey post hoc. Achieved WL 4.8 ± 1.1% BM; 51 ± 13% of lost mass regained during recovery. No familiarisation trial (authors attribute time effects to learning). Data only in Figures 3–6. Relative power for WL post computed with post-recovery body mass. The improvement occurred in both groups and is not attributable to weight loss.
Artioli et al., 2010 [36]	Control group (non-weight-cyclers; weight stable; n = 7)	Upper-body Wingate (3 × 30 s) – absolute mean power (W)	Pre-intervention vs post-intervention (5–7 d apart; WL tested after 4-h recovery)	Figure only	Figure only	'Discrete improvement' post vs pre (magnitude NR)	Main effect of time $p < 0.01$ (pre vs post, both groups); no group or interaction effects (F and p NR)	not reported	Yes (time main effect only; no interaction)	Favourable (time effect in both groups; attributed to learning)	Non-randomised allocation (weight-cyclers vs non-weight-cyclers). Mixed-model two-way ANOVA (time × group) with Tukey post hoc. Achieved WL 4.8 ± 1.1% BM; 51 ± 13% of lost mass regained during recovery. No familiarisation trial (authors attribute time effects to learning). Data only in Figures 3–6. Relative power for WL post computed with post-recovery body mass. The improvement occurred in both groups and is not attributable to weight loss.

Study (first author, year) [ref]	Group / condition	Outcome	Time point	Pre value (mean ± SD)	Post value (mean ± SD)	Change (absolute and/or %)	Test statistic and p value	Effect size (95% CI if reported)	Statistically significant (Yes / No)	Direction (Favourable / Unfavourable / Null)	Notes
Artioli et al., 2010 [36]	Weight loss group (weight-cyclers; ~5% BM in 5–7 d, then 4 h refeeding; n = 7)	Upper-body Wingate (3 × 30 s) – relative mean power (W/kg)	Pre-intervention vs post-intervention (5–7 d apart; WL tested after 4-h recovery)	Figure only	Figure only	'Discrete improvement' post vs pre (magnitude NR)	Main effect of time $p < 0.01$ (pre vs post, both groups); no group or interaction effects (F and p NR)	not reported	Yes (time main effect only; no interaction)	Favourable (time effect in both groups; attributed to learning)	Non-randomised allocation (weight-cyclers vs non-weight-cyclers). Mixed-model two-way ANOVA (time × group) with Tukey post hoc. Achieved WL 4.8 ± 1.1% BM; 51 ± 13% of lost mass regained during recovery. No familiarisation trial (authors attribute time effects to learning). Data only in Figures 3–6. Relative power for WL post computed with post-recovery body mass. The improvement occurred in both groups and is not attributable to weight loss.
Artioli et al., 2010 [36]	Control group (non-weight-cyclers; weight stable; n = 7)	Upper-body Wingate (3 × 30 s) – relative mean power (W/kg)	Pre-intervention vs post-intervention (5–7 d apart; WL tested after 4-h recovery)	Figure only	Figure only	'Discrete improvement' post vs pre (magnitude NR)	Main effect of time $p < 0.01$ (pre vs post, both groups); no group or interaction effects (F and p NR)	not reported	Yes (time main effect only; no interaction)	Favourable (time effect in both groups; attributed to learning)	Non-randomised allocation (weight-cyclers vs non-weight-cyclers). Mixed-model two-way ANOVA (time × group) with Tukey post hoc. Achieved WL 4.8 ± 1.1% BM; 51 ± 13% of lost mass regained during recovery. No familiarisation trial (authors attribute time effects to learning). Data only in Figures 3–6. Relative power for WL post computed with post-recovery body mass. The improvement occurred in both groups and is not attributable to weight loss.

Study (first author, year) [ref]	Group / condition	Outcome	Time point	Pre value (mean ± SD)	Post value (mean ± SD)	Change (absolute and/or %)	Test statistic and p value	Effect size (95% CI if reported)	Statistically significant (Yes / No)	Direction (Favourable / Unfavourable / Null)	Notes
Artioli et al., 2010 [36]	Weight loss group (weight-cyclers; ~5% BM in 5–7 d, then 4 h refeeding; n = 7)	Upper-body Wingate (3 × 30 s) – absolute peak power (W)	Pre-intervention vs post-intervention (5–7 d apart; WL tested after 4-h recovery)	Figure only	Figure only	'Discrete improvement' post vs pre (magnitude NR)	Main effect of time $p < 0.01$ (pre vs post, both groups); no group or interaction effects (F and p NR)	not reported	Yes (time main effect only; no interaction)	Favourable (time effect in both groups; attributed to learning)	Non-randomised allocation (weight-cyclers vs non-weight-cyclers). Mixed-model two-way ANOVA (time × group) with Tukey post hoc. Achieved WL 4.8 ± 1.1% BM; 51 ± 13% of lost mass regained during recovery. No familiarisation trial (authors attribute time effects to learning). Data only in Figures 3–6. Relative power for WL post computed with post-recovery body mass. The improvement occurred in both groups and is not attributable to weight loss.
Artioli et al., 2010 [36]	Control group (non-weight-cyclers; weight stable; n = 7)	Upper-body Wingate (3 × 30 s) – absolute peak power (W)	Pre-intervention vs post-intervention (5–7 d apart; WL tested after 4-h recovery)	Figure only	Figure only	'Discrete improvement' post vs pre (magnitude NR)	Main effect of time $p < 0.01$ (pre vs post, both groups); no group or interaction effects (F and p NR)	not reported	Yes (time main effect only; no interaction)	Favourable (time effect in both groups; attributed to learning)	Non-randomised allocation (weight-cyclers vs non-weight-cyclers). Mixed-model two-way ANOVA (time × group) with Tukey post hoc. Achieved WL 4.8 ± 1.1% BM; 51 ± 13% of lost mass regained during recovery. No familiarisation trial (authors attribute time effects to learning). Data only in Figures 3–6. Relative power for WL post computed with post-recovery body mass. The improvement occurred in both groups and is not attributable to weight loss.

Study (first author, year) [ref]	Group / condition	Outcome	Time point	Pre value (mean ± SD)	Post value (mean ± SD)	Change (absolute and/or %)	Test statistic and p value	Effect size (95% CI if reported)	Statistically significant (Yes / No)	Direction (Favourable / Unfavourable / Null)	Notes
Artioli et al., 2010 [36]	Weight loss group (weight-cyclers; ~5% BM in 5–7 d, then 4 h refeeding; n = 7)	Upper-body Wingate (3 × 30 s) – relative peak power (W/kg)	Pre-intervention vs post-intervention (5–7 d apart; WL tested after 4-h recovery)	Figure only	Figure only	'Discrete improvement' post vs pre (magnitude NR)	Main effect of time $p < 0.01$ (pre vs post, both groups); no group or interaction effects (F and p NR)	not reported	Yes (time main effect only; no interaction)	Favourable (time effect in both groups; attributed to learning)	Non-randomised allocation (weight-cyclers vs non-weight-cyclers). Mixed-model two-way ANOVA (time × group) with Tukey post hoc. Achieved WL 4.8 ± 1.1% BM; 51 ± 13% of lost mass regained during recovery. No familiarisation trial (authors attribute time effects to learning). Data only in Figures 3–6. Relative power for WL post computed with post-recovery body mass. The improvement occurred in both groups and is not attributable to weight loss.
Artioli et al., 2010 [36]	Control group (non-weight-cyclers; weight stable; n = 7)	Upper-body Wingate (3 × 30 s) – relative peak power (W/kg)	Pre-intervention vs post-intervention (5–7 d apart; WL tested after 4-h recovery)	Figure only	Figure only	'Discrete improvement' post vs pre (magnitude NR)	Main effect of time $p < 0.01$ (pre vs post, both groups); no group or interaction effects (F and p NR)	not reported	Yes (time main effect only; no interaction)	Favourable (time effect in both groups; attributed to learning)	Non-randomised allocation (weight-cyclers vs non-weight-cyclers). Mixed-model two-way ANOVA (time × group) with Tukey post hoc. Achieved WL 4.8 ± 1.1% BM; 51 ± 13% of lost mass regained during recovery. No familiarisation trial (authors attribute time effects to learning). Data only in Figures 3–6. Relative power for WL post computed with post-recovery body mass. The improvement occurred in both groups and is not attributable to weight loss.

Study (first author, year) [ref]	Group / condition	Outcome	Time point	Pre value (mean ± SD)	Post value (mean ± SD)	Change (absolute and/or %)	Test statistic and p value	Effect size (95% CI if reported)	Statistically significant (Yes / No)	Direction (Favourable / Unfavourable / Null)	Notes
Artioli et al., 2010 [36]	Weight loss group (weight-cyclers; ~5% BM in 5–7 d, then 4 h refeeding; n = 7)	Upper-body Wingate (3 × 30 s) – absolute total work (J)	Pre-intervention vs post-intervention (5–7 d apart; WL tested after 4-h recovery)	Figure only	Figure only	'Discrete improvement' post vs pre (magnitude NR)	Main effect of time p < 0.01 (pre vs post, both groups); no group or interaction effects (F and p NR)	not reported	Yes (time main effect only; no interaction)	Favourable (time effect in both groups; attributed to learning)	Non-randomised allocation (weight-cyclers vs non-weight-cyclers). Mixed-model two-way ANOVA (time × group) with Tukey post hoc. Achieved WL 4.8 ± 1.1% BM; 51 ± 13% of lost mass regained during recovery. No familiarisation trial (authors attribute time effects to learning). Data only in Figures 3–6. Relative power for WL post computed with post-recovery body mass. The improvement occurred in both groups and is not attributable to weight loss.
Artioli et al., 2010 [36]	Control group (non-weight-cyclers; weight stable; n = 7)	Upper-body Wingate (3 × 30 s) – absolute total work (J)	Pre-intervention vs post-intervention (5–7 d apart; WL tested after 4-h recovery)	Figure only	Figure only	'Discrete improvement' post vs pre (magnitude NR)	Main effect of time p < 0.01 (pre vs post, both groups); no group or interaction effects (F and p NR)	not reported	Yes (time main effect only; no interaction)	Favourable (time effect in both groups; attributed to learning)	Non-randomised allocation (weight-cyclers vs non-weight-cyclers). Mixed-model two-way ANOVA (time × group) with Tukey post hoc. Achieved WL 4.8 ± 1.1% BM; 51 ± 13% of lost mass regained during recovery. No familiarisation trial (authors attribute time effects to learning). Data only in Figures 3–6. Relative power for WL post computed with post-recovery body mass. The improvement occurred in both groups and is not attributable to weight loss.

Study (first author, year) [ref]	Group / condition	Outcome	Time point	Pre value (mean ± SD)	Post value (mean ± SD)	Change (absolute and/or %)	Test statistic and p value	Effect size (95% CI if reported)	Statistically significant (Yes / No)	Direction (Favourable / Unfavourable / Null)	Notes
Artioli et al., 2010 [36]	Weight loss group (weight-cyclers; ~5% BM in 5–7 d, then 4 h refeeding; n = 7)	Upper-body Wingate (3 × 30 s) – relative total work (J/kg)	Pre-intervention vs post-intervention (5–7 d apart; WL tested after 4-h recovery)	Figure only	Figure only	'Discrete improvement' post vs pre (magnitude NR)	Main effect of time $p < 0.01$ (pre vs post, both groups); no group or interaction effects (F and p NR)	not reported	Yes (time main effect only; no interaction)	Favourable (time effect in both groups; attributed to learning)	Non-randomised allocation (weight-cyclers vs non-weight-cyclers). Mixed-model two-way ANOVA (time × group) with Tukey post hoc. Achieved WL 4.8 ± 1.1% BM; 51 ± 13% of lost mass regained during recovery. No familiarisation trial (authors attribute time effects to learning). Data only in Figures 3–6. Relative power for WL post computed with post-recovery body mass. The improvement occurred in both groups and is not attributable to weight loss.
Artioli et al., 2010 [36]	Control group (non-weight-cyclers; weight stable; n = 7)	Upper-body Wingate (3 × 30 s) – relative total work (J/kg)	Pre-intervention vs post-intervention (5–7 d apart; WL tested after 4-h recovery)	Figure only	Figure only	'Discrete improvement' post vs pre (magnitude NR)	Main effect of time $p < 0.01$ (pre vs post, both groups); no group or interaction effects (F and p NR)	not reported	Yes (time main effect only; no interaction)	Favourable (time effect in both groups; attributed to learning)	Non-randomised allocation (weight-cyclers vs non-weight-cyclers). Mixed-model two-way ANOVA (time × group) with Tukey post hoc. Achieved WL 4.8 ± 1.1% BM; 51 ± 13% of lost mass regained during recovery. No familiarisation trial (authors attribute time effects to learning). Data only in Figures 3–6. Relative power for WL post computed with post-recovery body mass. The improvement occurred in both groups and is not attributable to weight loss.

Study (first author, year) [ref]	Group / condition	Outcome	Time point	Pre value (mean ± SD)	Post value (mean ± SD)	Change (absolute and/or %)	Test statistic and p value	Effect size (95% CI if reported)	Statistically significant (Yes / No)	Direction (Favourable / Unfavourable / Null)	Notes
Artioli et al., 2010 [36]	Weight loss group (weight-cyclers; ~5% BM in 5–7 d, then 4 h refeeding; n = 7)	Plasma lactate at rest and 3 min after uchi-komi, combat and Wingate (mmol/L)	Pre-intervention vs post-intervention (5–7 d apart; WL tested after 4-h recovery)	Figure only	Figure only	not reported	No differences between groups or conditions (p NR)	not reported	No	Null	Non-randomised allocation (weight-cyclers vs non-weight-cyclers). Mixed-model two-way ANOVA (time × group) with Tukey post hoc. Achieved WL 4.8 ± 1.1% BM; 51 ± 13% of lost mass regained during recovery. No familiarisation trial (authors attribute time effects to learning). Physiological marker; Figure 7.
Artioli et al., 2010 [36]	Control group (non-weight-cyclers; weight stable; n = 7)	Plasma lactate at rest and 3 min after uchi-komi, combat and Wingate (mmol/L)	Pre-intervention vs post-intervention (5–7 d apart; WL tested after 4-h recovery)	Figure only	Figure only	not reported	No differences between groups or conditions (p NR)	not reported	No	Null	Non-randomised allocation (weight-cyclers vs non-weight-cyclers). Mixed-model two-way ANOVA (time × group) with Tukey post hoc. Achieved WL 4.8 ± 1.1% BM; 51 ± 13% of lost mass regained during recovery. No familiarisation trial (authors attribute time effects to learning). Physiological marker; Figure 7.
Ceylan et al., 2022 [37]	DEHY (dehydration: 3 h cycling 60 W, 40 °C/40% RH, sauna suit; n = 9)	Maximal handgrip strength, right (HGS RIGHT) (kgf)	1 h after the protocol (1-h refeed/rehydration); no pre-intervention performance test	not reported	47.6 ± 3.8	Delta change 3.8%	Paired t-test, p = 0.004	Cohen's d = 1.34 (95% CI 0.40–2.23)	Yes	Unfavourable	Crossover (7-d washout). Comparison is DEHY vs CON at the same time point.

Study (first author, year) [ref]	Group / condition	Outcome	Time point	Pre value (mean ± SD)	Post value (mean ± SD)	Change (absolute and/or %)	Test statistic and p value	Effect size (95% CI if reported)	Statistically significant (Yes / No)	Direction (Favourable / Unfavourable / Null)	Notes
Ceylan et al., 2022 [37]	CON (3 h cycling 60 W, thermoneutral, fluids allowed; n = 9)	Maximal handgrip strength, right (HGS RIGHT) (kgf)	1 h after the protocol (1-h refeed/rehydration); no pre-intervention performance test	not reported	49.5 ± 4.5	not reported	Reference condition (comparison reported in DEHY row)	see DEHY row	n/a (reference)	n/a (reference)	—
Ceylan et al., 2022 [37]	DEHY (dehydration: 3 h cycling 60 W, 40 °C/40% RH, sauna suit; n = 9)	Maximal handgrip strength, left (HGS LEFT) (kgf)	1 h after the protocol (1-h refeed/rehydration); no pre-intervention performance test	not reported	45.7 ± 5.4	Delta change 4.4%	Paired t-test, $p = 0.037$	Cohen's d = 0.83 (95% CI 0.04–1.58)	Yes	Unfavourable	Crossover (7-d washout). Comparison is DEHY vs CON at the same time point.
Ceylan et al., 2022 [37]	CON (3 h cycling 60 W, thermoneutral, fluids allowed; n = 9)	Maximal handgrip strength, left (HGS LEFT) (kgf)	1 h after the protocol (1-h refeed/rehydration); no pre-intervention performance test	not reported	47.8 ± 4.6	not reported	Reference condition (comparison reported in DEHY row)	see DEHY row	n/a (reference)	n/a (reference)	—
Ceylan et al., 2022 [37]	DEHY (dehydration: 3 h cycling 60 W, 40 °C/40% RH, sauna suit; n = 9)	Judogi grip strength test, isometric (JGST ISO) (s)	1 h after the protocol (1-h refeed/rehydration); no pre-intervention performance test	not reported	45.8 ± 10.6	Delta change 14.6%	Paired t-test, $p < 0.001$	Cohen's d = 1.88 (95% CI 0.74–2.97)	Yes	Unfavourable	Crossover (7-d washout). Comparison is DEHY vs CON at the same time point.
Ceylan et al., 2022 [37]	CON (3 h cycling 60 W, thermoneutral, fluids allowed; n = 9)	Judogi grip strength test, isometric (JGST ISO) (s)	1 h after the protocol (1-h refeed/rehydration); no pre-intervention performance test	not reported	53.6 ± 11.5	not reported	Reference condition (comparison reported in DEHY row)	see DEHY row	n/a (reference)	n/a (reference)	—

Study (first author, year) [ref]	Group / condition	Outcome	Time point	Pre value (mean ± SD)	Post value (mean ± SD)	Change (absolute and/or %)	Test statistic and p value	Effect size (95% CI if reported)	Statistically significant (Yes / No)	Direction (Favourable / Unfavourable / Null)	Notes
Ceylan et al., 2022 [37]	DEHY (dehydration: 3 h cycling 60 W, 40 °C/40% RH, sauna suit; n = 9)	Judogi grip strength test, dynamic (JGST DIN) (repetitions)	1 h after the protocol (1-h refeed/rehydration); no pre-intervention performance test	not reported	16 ± 4	Delta change 23.8%	Paired t-test, $p = 0.005$	Cohen's d = 1.28 (95% CI 0.36–2.16)	Yes	Unfavourable	Crossover (7-d washout). Comparison is DEHY vs CON at the same time point.
Ceylan et al., 2022 [37]	CON (3 h cycling 60 W, thermoneutral, fluids allowed; n = 9)	Judogi grip strength test, dynamic (JGST DIN) (repetitions)	1 h after the protocol (1-h refeed/rehydration); no pre-intervention performance test	not reported	21 ± 3	not reported	Reference condition (comparison reported in DEHY row)	see DEHY row	n/a (reference)	n/a (reference)	—
Ceylan et al., 2022 [37]	DEHY (dehydration: 3 h cycling 60 W, 40 °C/40% RH, sauna suit; n = 9)	SJFT index (bpm·throws^{-1})	1 h after the protocol (1-h refeed/rehydration); no pre-intervention performance test	not reported	12.90 ± 1.16	Delta change reported as −1.3%	Paired t-test, $p < 0.001$	not reported	Yes	Unfavourable	Crossover (7-d washout). Comparison is DEHY vs CON at the same time point. Higher index = worse performance. The reported −1.3% delta change is inconsistent with the means (11.67→12.90 = +10.5%).
Ceylan et al., 2022 [37]	CON (3 h cycling 60 W, thermoneutral, fluids allowed; n = 9)	SJFT index (bpm·throws^{-1})	1 h after the protocol (1-h refeed/rehydration); no pre-intervention performance test	not reported	11.67 ± 0.94	not reported	Reference condition (comparison reported in DEHY row)	see DEHY row	n/a (reference)	n/a (reference)	—
Ceylan et al., 2022 [37]	DEHY (dehydration: 3 h cycling 60 W, 40 °C/40% RH, sauna suit; n = 9)	SJFT – number of throws, set A (15 s)	1 h after the protocol (1-h refeed/rehydration); no pre-intervention performance test	not reported	Figure only	Delta change 8% (text)	$p < 0.05$ (Figure 2)	not reported	Yes	Unfavourable	Crossover (7-d washout). Comparison is DEHY vs CON at the same time point. Throw counts shown only in Figure 2.

Study (first author, year) [ref]	Group / condition	Outcome	Time point	Pre value (mean ± SD)	Post value (mean ± SD)	Change (absolute and/or %)	Test statistic and p value	Effect size (95% CI if reported)	Statistically significant (Yes / No)	Direction (Favourable / Unfavourable / Null)	Notes
Ceylan et al., 2022 [37]	CON (3 h cycling 60 W, thermoneutral, fluids allowed; n = 9)	SJFT – number of throws, set A (15 s)	1 h after the protocol (1-h refeed/rehydration); no pre-intervention performance test	not reported	Figure only	not reported	Reference condition (comparison reported in DEHY row)	see DEHY row	n/a (reference)	n/a (reference)	—
Ceylan et al., 2022 [37]	DEHY (dehydration: 3 h cycling 60 W, 40 °C/40% RH, sauna suit; n = 9)	SJFT – number of throws, set B (30 s)	1 h after the protocol (1-h refeed/rehydration); no pre-intervention performance test	not reported	Figure only	not reported	Not significant (no asterisk in Figure 2; p NR)	not reported	No	Null	Crossover (7-d washout). Comparison is DEHY vs CON at the same time point. Data only in Figure 2.
Ceylan et al., 2022 [37]	CON (3 h cycling 60 W, thermoneutral, fluids allowed; n = 9)	SJFT – number of throws, set B (30 s)	1 h after the protocol (1-h refeed/rehydration); no pre-intervention performance test	not reported	Figure only	not reported	Reference condition (comparison reported in DEHY row)	see DEHY row	n/a (reference)	n/a (reference)	—
Ceylan et al., 2022 [37]	DEHY (dehydration: 3 h cycling 60 W, 40 °C/40% RH, sauna suit; n = 9)	SJFT – number of throws, set C (30 s)	1 h after the protocol (1-h refeed/rehydration); no pre-intervention performance test	not reported	Figure only	Delta change 7.9% (text)	$p < 0.05$ (Figure 2)	not reported	Yes	Unfavourable	Crossover (7-d washout). Comparison is DEHY vs CON at the same time point. Data only in Figure 2.
Ceylan et al., 2022 [37]	CON (3 h cycling 60 W, thermoneutral, fluids allowed; n = 9)	SJFT – number of throws, set C (30 s)	1 h after the protocol (1-h refeed/rehydration); no pre-intervention performance test	not reported	Figure only	not reported	Reference condition (comparison reported in DEHY row)	see DEHY row	n/a (reference)	n/a (reference)	—

Study (first author, year) [ref]	Group / condition	Outcome	Time point	Pre value (mean ± SD)	Post value (mean ± SD)	Change (absolute and/or %)	Test statistic and p value	Effect size (95% CI if reported)	Statistically significant (Yes / No)	Direction (Favourable / Unfavourable / Null)	Notes
Ceylan et al., 2022 [37]	DEHY (dehydration: 3 h cycling 60 W, 40 °C/40% RH, sauna suit; n = 9)	SJFT – total throws (TT)	1 h after the protocol (1-h refeed/rehydration); no pre-intervention performance test	not reported	Figure only	not reported	Shown in Figure 2; significance not stated in text (p NR)	not reported	not reported	not reported	Crossover (7-d washout). Comparison is DEHY vs CON at the same time point. Total throws appear only in Figure 2; unclear whether the difference was significant.
Ceylan et al., 2022 [37]	CON (3 h cycling 60 W, thermoneutral, fluids allowed; n = 9)	SJFT – total throws (TT)	1 h after the protocol (1-h refeed/rehydration); no pre-intervention performance test	not reported	Figure only	not reported	Reference condition (comparison reported in DEHY row)	see DEHY row	n/a (reference)	n/a (reference)	—
Ceylan et al., 2022 [37]	DEHY (dehydration: 3 h cycling 60 W, 40 °C/40% RH, sauna suit; n = 9)	Heart rate during SJFT, sets A–C (bpm)	1 h after the protocol (1-h refeed/rehydration); no pre-intervention performance test	not reported	Figure only	not reported	Higher in DEHY in sets B and C ($p < 0.05$, Figure 2); not in set A	not reported	Yes (sets B and C)	Unfavourable	Crossover (7-d washout). Comparison is DEHY vs CON at the same time point. Data only in Figure 2.
Ceylan et al., 2022 [37]	CON (3 h cycling 60 W, thermoneutral, fluids allowed; n = 9)	Heart rate during SJFT, sets A–C (bpm)	1 h after the protocol (1-h refeed/rehydration); no pre-intervention performance test	not reported	Figure only	not reported	Reference condition (comparison reported in DEHY row)	see DEHY row	n/a (reference)	n/a (reference)	—
Ceylan et al., 2022 [37]	DEHY (dehydration: 3 h cycling 60 W, 40 °C/40% RH, sauna suit; n = 9)	Heart rate 1 min after SJFT (HR1minpost) (bpm)	1 h after the protocol (1-h refeed/rehydration); no pre-intervention performance test	not reported	150.7 ± 10.6	not reported	RM ANOVA (Table 1): intervention $F = 5.97$ ($p < 0.05$); time $F = 469.56$ ($p < 0.001$); intervention × time $F = 1.83$ (n.s.)	not reported	No (interaction); intervention main effect Yes	Null	Crossover (7-d washout). Comparison is DEHY vs CON at the same time point. Text states that 'HR did not change regardless of the condition', whereas Table 1 marks the intervention effect as $p < 0.05$.

Study (first author, year) [ref]	Group / condition	Outcome	Time point	Pre value (mean ± SD)	Post value (mean ± SD)	Change (absolute and/or %)	Test statistic and p value	Effect size (95% CI if reported)	Statistically significant (Yes / No)	Direction (Favourable / Unfavourable / Null)	Notes
Ceylan et al., 2022 [37]	CON (3 h cycling 60 W, thermoneutral, fluids allowed; n = 9)	Heart rate 1 min after SJFT (HR1minpost) (bpm)	1 h after the protocol (1-h refeed/rehydration); no pre-intervention performance test	not reported	144.2 ± 14.2	not reported	Reference condition (comparison reported in DEHY row)	see DEHY row	n/a (reference)	n/a (reference)	—
Ceylan et al., 2022 [37]	DEHY (dehydration: 3 h cycling 60 W, 40 °C/40% RH, sauna suit; n = 9)	Blood lactate 1 min after SJFT (mmol/L)	1 h after the protocol (1-h refeed/rehydration); no pre-intervention performance test	not reported	17.01 ± 3.8 (text) / 17.0 ± 3.8 (Table 1)	not reported	p = 0.119 (text); Table 1: intervention F = 9.12 (significance symbol absent), time F = 169.12 (p < 0.001), interaction F = 2.29	not reported	No	Null	Crossover (7-d washout). Comparison is DEHY vs CON at the same time point. Minor text/table rounding differences; significance of the intervention F in Table 1 unclear.
Ceylan et al., 2022 [37]	CON (3 h cycling 60 W, thermoneutral, fluids allowed; n = 9)	Blood lactate 1 min after SJFT (mmol/L)	1 h after the protocol (1-h refeed/rehydration); no pre-intervention performance test	not reported	14.92 ± 3.57 (text) / 14.9 ± 3.5 (Table 1)	not reported	Reference condition (comparison reported in DEHY row)	see DEHY row	n/a (reference)	n/a (reference)	—
Connor et al., 2022 [38]	FWB (RWL incl. hot fresh-water bathing; n = 13)	Countermovement jump (CMJ) height (cm)	Day −2 (pre-RWL) vs Day +1 (~24–26 h after bathing, after recovery)	32.93 ± 2.71	32.86 ± 3.30	not reported	Two-way RM ANOVA (condition × time): Time p = 0.572; condition p = 0.080; interaction p = 0.435	Cohen's d NR for performance outcomes	No	Null	Crossover; both conditions are RWL (~26–28 h fluid/diet restriction + 2 h hot bath/wrap; total loss ≈5.3% BM) followed by ~24–26 h recovery; no no-weight-loss control. Participants were male mixed martial arts athletes (article title and Methods). Familiarisation values and order-effect tests (all p > 0.28) also reported; smallest worthwhile difference defined as 0.2 × between-subject SD.

Study (first author, year) [ref]	Group / condition	Outcome	Time point	Pre value (mean ± SD)	Post value (mean ± SD)	Change (absolute and/or %)	Test statistic and p value	Effect size (95% CI if reported)	Statistically significant (Yes / No)	Direction (Favourable / Unfavourable / Null)	Notes
Connor et al., 2022 [38]	SWB (RWL incl. hot salt-water bathing, ~5% Epsom salt; n = 13)	Countermovement jump (CMJ) height (cm)	Day −2 (pre-RWL) vs Day +1 (~24–26 h after bathing, after recovery)	31.81 ± 3.86	32.29 ± 3.06	not reported	Two-way RM ANOVA (condition × time): Time p = 0.572; condition p = 0.080; interaction p = 0.435	Cohen's d NR for performance outcomes	No	Null	Crossover; both conditions are RWL (~26–28 h fluid/diet restriction + 2 h hot bath/wrap; total loss ≈5.3% BM) followed by ~24–26 h recovery; no no-weight-loss control. Participants were male mixed martial arts athletes (article title and Methods). Familiarisation values and order-effect tests (all p > 0.28) also reported; smallest worthwhile difference defined as 0.2 × between-subject SD.
Connor et al., 2022 [38]	FWB (RWL incl. hot fresh-water bathing; n = 13)	Hand-grip strength (kg; mean of best left and right)	Day −2 (pre-RWL) vs Day +1 (~24–26 h after bathing, after recovery)	48.0 ± 6.8	48.9 ± 7.9	not reported	Two-way RM ANOVA (condition × time): Time p = 0.503; condition p = 0.739; interaction p = 0.066	Cohen's d NR for performance outcomes	No	Null	Crossover; both conditions are RWL (~26–28 h fluid/diet restriction + 2 h hot bath/wrap; total loss ≈5.3% BM) followed by ~24–26 h recovery; no no-weight-loss control. Participants were male mixed martial arts athletes (article title and Methods). Familiarisation values and order-effect tests (all p > 0.28) also reported; smallest worthwhile difference defined as 0.2 × between-subject SD.

Study (first author, year) [ref]	Group / condition	Outcome	Time point	Pre value (mean ± SD)	Post value (mean ± SD)	Change (absolute and/or %)	Test statistic and p value	Effect size (95% CI if reported)	Statistically significant (Yes / No)	Direction (Favourable / Unfavourable / Null)	Notes
Connor et al., 2022 [38]	SWB (RWL incl. hot salt-water bathing, ~5% Epsom salt; n = 13)	Hand-grip strength (kg; mean of best left and right)	Day −2 (pre-RWL) vs Day +1 (~24–26 h after bathing, after recovery)	48.8 ± 8.0	47.5 ± 7.6	not reported	Two-way RM ANOVA (condition × time): Time p = 0.503; condition p = 0.739; interaction p = 0.066	Cohen's d NR for performance outcomes	No	Null	Crossover; both conditions are RWL (~26–28 h fluid/diet restriction + 2 h hot bath/wrap; total loss ≈5.3% BM) followed by ~24–26 h recovery; no no-weight-loss control. Participants were male mixed martial arts athletes (article title and Methods). Familiarisation values and order-effect tests (all p > 0.28) also reported; smallest worthwhile difference defined as 0.2 × between-subject SD.
Connor et al., 2022 [38]	FWB (RWL incl. hot fresh-water bathing; n = 13)	Isometric mid-thigh pull (IMTP) peak force (N)	Day −2 (pre-RWL) vs Day +1 (~24–26 h after bathing, after recovery)	1690 ± 267	1757 ± 276	not reported	Two-way RM ANOVA (condition × time): Time p = 0.435; condition p = 0.785; interaction p = 0.152	Cohen's d NR for performance outcomes	No	Null	Crossover; both conditions are RWL (~26–28 h fluid/diet restriction + 2 h hot bath/wrap; total loss ≈5.3% BM) followed by ~24–26 h recovery; no no-weight-loss control. Participants were male mixed martial arts athletes (article title and Methods). Familiarisation values and order-effect tests (all p > 0.28) also reported; smallest worthwhile difference defined as 0.2 × between-subject SD.

Study (first author, year) [ref]	Group / condition	Outcome	Time point	Pre value (mean ± SD)	Post value (mean ± SD)	Change (absolute and/or %)	Test statistic and p value	Effect size (95% CI if reported)	Statistically significant (Yes / No)	Direction (Favourable / Unfavourable / Null)	Notes
Connor et al., 2022 [38]	SWB (RWL incl. hot salt-water bathing, ~5% Epsom salt; n = 13)	Isometric mid-thigh pull (IMTP) peak force (N)	Day −2 (pre-RWL) vs Day +1 (~24–26 h after bathing, after recovery)	1739 ± 288	1729 ± 287	not reported	Two-way RM ANOVA (condition × time): Time p = 0.435; condition p = 0.785; interaction p = 0.152	Cohen's d NR for performance outcomes	No	Null	Crossover; both conditions are RWL (~26–28 h fluid/diet restriction + 2 h hot bath/wrap; total loss ≈5.3% BM) followed by ~24–26 h recovery; no no-weight-loss control. Participants were male mixed martial arts athletes (article title and Methods). Familiarisation values and order-effect tests (all p > 0.28) also reported; smallest worthwhile difference defined as 0.2 × between-subject SD.
Connor et al., 2022 [38]	FWB (RWL incl. hot fresh-water bathing; n = 13)	3-min all-out test – functional threshold power (FTP) (W)	Day −2 (pre-RWL) vs Day +1 (~24–26 h after bathing, after recovery)	219.6 ± 22.9	220.6 ± 26.4	not reported	Two-way RM ANOVA (condition × time): Time p = 0.642; condition p = 0.068; interaction p = 0.854	Cohen's d NR for performance outcomes	No	Null	Crossover; both conditions are RWL (~26–28 h fluid/diet restriction + 2 h hot bath/wrap; total loss ≈5.3% BM) followed by ~24–26 h recovery; no no-weight-loss control. Participants were male mixed martial arts athletes (article title and Methods). Familiarisation values and order-effect tests (all p > 0.28) also reported; smallest worthwhile difference defined as 0.2 × between-subject SD.

Study (first author, year) [ref]	Group / condition	Outcome	Time point	Pre value (mean ± SD)	Post value (mean ± SD)	Change (absolute and/or %)	Test statistic and p value	Effect size (95% CI if reported)	Statistically significant (Yes / No)	Direction (Favourable / Unfavourable / Null)	Notes
Connor et al., 2022 [38]	SWB (RWL incl. hot salt-water bathing, ~5% Epsom salt; n = 13)	3-min all-out test – functional threshold power (FTP) (W)	Day −2 (pre-RWL) vs Day +1 (~24–26 h after bathing, after recovery)	222.2 ± 25.4	223.7 ± 27.8	not reported	Two-way RM ANOVA (condition × time): Time p = 0.642; condition p = 0.068; interaction p = 0.854	Cohen's d NR for performance outcomes	No	Null	Crossover; both conditions are RWL (~26–28 h fluid/diet restriction + 2 h hot bath/wrap; total loss ≈5.3% BM) followed by ~24–26 h recovery; no no-weight-loss control. Participants were male mixed martial arts athletes (article title and Methods). Familiarisation values and order-effect tests (all p > 0.28) also reported; smallest worthwhile difference defined as 0.2 × between-subject SD.
Connor et al., 2022 [38]	FWB (RWL incl. hot fresh-water bathing; n = 13)	3-min all-out test – maximum heart rate (bpm)	Day −2 (pre-RWL) vs Day +1 (~24–26 h after bathing, after recovery)	183.5 ± 10.1	185.2 ± 11.4	not reported	Two-way RM ANOVA (condition × time): Time p = 0.374; condition p = 0.228; interaction p = 0.790	Cohen's d NR for performance outcomes	No	Null	Crossover; both conditions are RWL (~26–28 h fluid/diet restriction + 2 h hot bath/wrap; total loss ≈5.3% BM) followed by ~24–26 h recovery; no no-weight-loss control. Participants were male mixed martial arts athletes (article title and Methods). Familiarisation values and order-effect tests (all p > 0.28) also reported; smallest worthwhile difference defined as 0.2 × between-subject SD.

Study (first author, year) [ref]	Group / condition	Outcome	Time point	Pre value (mean ± SD)	Post value (mean ± SD)	Change (absolute and/or %)	Test statistic and p value	Effect size (95% CI if reported)	Statistically significant (Yes / No)	Direction (Favourable / Unfavourable / Null)	Notes
Connor et al., 2022 [38]	SWB (RWL incl. hot salt-water bathing, ~5% Epsom salt; n = 13)	3-min all-out test – maximum heart rate (bpm)	Day −2 (pre-RWL) vs Day +1 (~24–26 h after bathing, after recovery)	185.2 ± 12.4	186.1 ± 11.4	not reported	Two-way RM ANOVA (condition × time): Time p = 0.374; condition p = 0.228; interaction p = 0.790	Cohen's d NR for performance outcomes	No	Null	Crossover; both conditions are RWL (~26–28 h fluid/diet restriction + 2 h hot bath/wrap; total loss ≈5.3% BM) followed by ~24–26 h recovery; no no-weight-loss control. Participants were male mixed martial arts athletes (article title and Methods). Familiarisation values and order-effect tests (all p > 0.28) also reported; smallest worthwhile difference defined as 0.2 × between-subject SD.
Isacco et al., 2020 [39]	WL (weight loss ≥3% BM in the week before; n = 10)	POMS – Tension	After each of 5 simulated fights (F1–F5, 30-min rest); T0 assessed but not tabulated	T0 NR; F1 = 39 ± 6	F5 = 36 ± 5 (F2 35 ± 3; F3 36 ± 5; F4 35 ± 5)	not reported	Random-effects model on AUC; time effect reported (see notes); group effect p = 0.28; time × group interaction p: F2 = 0.66, F3 = 0.28, F4 = 0.08, F5 = 0.01[5] (Sidak post hoc)	Hedges' g reported only in supplementary Table 4 (not available)	Yes at F5 only (interaction); No at F4	Unclear (WL and WS equal at F5)	Table p values reconstructed from a column-wrapped PDF; digits in [ ] were displaced in the text layer and should be checked against the typeset table. Text states significant interactions at F4 and F5 for every POMS dimension, but the table gives p = 0.08 at F4 for Tension.
Isacco et al., 2020 [39]	WS (weight stable; n = 10)	POMS – Tension	After each of 5 simulated fights (F1–F5, 30-min rest); T0 assessed but not tabulated	T0 NR; F1 = 40 ± 9	F5 = 36 ± 5 (F2 39 ± 8; F3 38 ± 7; F4 38 ± 7)	not reported	Comparator for the interaction (see WL row)	see WL row	n/a (comparator)	n/a (comparator)	—

Study (first author, year) [ref]	Group / condition	Outcome	Time point	Pre value (mean ± SD)	Post value (mean ± SD)	Change (absolute and/or %)	Test statistic and p value	Effect size (95% CI if reported)	Statistically significant (Yes / No)	Direction (Favourable / Unfavourable / Null)	Notes
Isacco et al., 2020 [39]	WL (weight loss ≥3% BM in the week before; n = 10)	POMS – Depression	After each of 5 simulated fights (F1–F5, 30-min rest); T0 assessed but not tabulated	T0 NR; F1 = 37 ± 3	F5 = 38 ± 4 (F2 37 ± 3; F3 37 ± 3; F4 38 ± 5)	not reported	Random-effects model on AUC; time effect reported (see notes); group effect p = 0.13[1]; time × group interaction p: F2 = 0.55, F3 = 0.14, F4 = 0.01, F5 = 0.00[1] (Sidak post hoc)	Hedges' g reported only in supplementary Table 4 (not available)	Yes (interaction at F4 and F5)	Unfavourable	Table p values reconstructed from a column-wrapped PDF; digits in [ ] were displaced in the text layer and should be checked against the typeset table.
Isacco et al., 2020 [39]	WS (weight stable; n = 10)	POMS – Depression	After each of 5 simulated fights (F1–F5, 30-min rest); T0 assessed but not tabulated	T0 NR; F1 = 36 ± 1	F5 = 36 ± 2 (F2 37 ± 2; F3 36 ± 2; F4 36 ± 2)	not reported	Comparator for the interaction (see WL row)	see WL row	n/a (comparator)	n/a (comparator)	—
Isacco et al., 2020 [39]	WL (weight loss ≥3% BM in the week before; n = 10)	POMS – Anger	After each of 5 simulated fights (F1–F5, 30-min rest); T0 assessed but not tabulated	T0 NR; F1 = 42 ± 4	F5 = 44 ± 4 (F2 42 ± 2; F3 44 ± 4; F4 43 ± 7)	not reported	Random-effects model on AUC; time effect reported (see notes); group effect p = 0.2[4]; time × group interaction p: F2 = 0.61, F3 = 0.21, F4 = 0.04, F5 = 0.00[4] (Sidak post hoc)	Hedges' g reported only in supplementary Table 4 (not available)	Yes (interaction at F4 and F5)	Unfavourable	Table p values reconstructed from a column-wrapped PDF; digits in [ ] were displaced in the text layer and should be checked against the typeset table. Direction inferred from F5 means only (WL 44 vs WS 43); authors do not state the direction of each interaction.
Isacco et al., 2020 [39]	WS (weight stable; n = 10)	POMS – Anger	After each of 5 simulated fights (F1–F5, 30-min rest); T0 assessed but not tabulated	T0 NR; F1 = 42 ± 3	F5 = 43 ± 4 (F2 45 ± 9; F3 44 ± 7; F4 44 ± 7)	not reported	Comparator for the interaction (see WL row)	see WL row	n/a (comparator)	n/a (comparator)	—

Study (first author, year) [ref]	Group / condition	Outcome	Time point	Pre value (mean ± SD)	Post value (mean ± SD)	Change (absolute and/or %)	Test statistic and p value	Effect size (95% CI if reported)	Statistically significant (Yes / No)	Direction (Favourable / Unfavourable / Null)	Notes
Isacco et al., 2020 [39]	WL (weight loss ≥3% BM in the week before; n = 10)	POMS – Vigour	After each of 5 simulated fights (F1–F5, 30-min rest); T0 assessed but not tabulated	T0 NR; F1 = 53 ± 11	F5 = 49 ± 12 (F2 52 ± 8; F3 50 ± 10; F4 47 ± 9)	not reported	Random-effects model on AUC; time effect reported (see notes); group effect p = 0.18; time × group interaction p: F2 = 0.56, F3 = 0.16, F4 = 0.034, F5 = 0.00[5] (Sidak post hoc)	Hedges' g reported only in supplementary Table 4 (not available)	Yes (interaction at F4 and F5)	Unfavourable	Table p values reconstructed from a column-wrapped PDF; digits in [ ] were displaced in the text layer and should be checked against the typeset table.
Isacco et al., 2020 [39]	WS (weight stable; n = 10)	POMS – Vigour	After each of 5 simulated fights (F1–F5, 30-min rest); T0 assessed but not tabulated	T0 NR; F1 = 54 ± 4	F5 = 53 ± 9 (F2 51 ± 5; F3 51 ± 6; F4 49 ± 5)	not reported	Comparator for the interaction (see WL row)	see WL row	n/a (comparator)	n/a (comparator)	—
Isacco et al., 2020 [39]	WL (weight loss ≥3% BM in the week before; n = 10)	POMS – Fatigue	After each of 5 simulated fights (F1–F5, 30-min rest); T0 assessed but not tabulated	T0 NR; F1 = 47 ± 5	F5 = 53 ± 9 (F2 47 ± 9; F3 48 ± 7; F4 50 ± 9)	not reported	Random-effects model on AUC; time effect reported (see notes); group effect p = 0.13; time × group interaction p: F2 = 0.56, F3 = 0.16, F4 = 0.02, F5 = 0.00[1] (Sidak post hoc)	Hedges' g reported only in supplementary Table 4 (not available)	Yes (interaction at F4 and F5)	Unfavourable	Table p values reconstructed from a column-wrapped PDF; digits in [ ] were displaced in the text layer and should be checked against the typeset table.
Isacco et al., 2020 [39]	WS (weight stable; n = 10)	POMS – Fatigue	After each of 5 simulated fights (F1–F5, 30-min rest); T0 assessed but not tabulated	T0 NR; F1 = 46 ± 6	F5 = 50 ± 5 (F2 47 ± 7; F3 47 ± 6; F4 47 ± 6)	not reported	Comparator for the interaction (see WL row)	see WL row	n/a (comparator)	n/a (comparator)	—
Isacco et al., 2020 [39]	WL (weight loss ≥3% BM in the week before; n = 10)	POMS – Confusion	After each of 5 simulated fights (F1–F5, 30-min rest); T0 assessed but not tabulated	T0 NR; F1 = 41 ± 5	F5 = 40 ± 7 (F2 40 ± 4; F3 39 ± 4; F4 41 ± 6)	not reported	Random-effects model on AUC; time effect reported (see notes); group effect p = 0.16; time × group interaction p: F2 = 0.55, F3 = 0.16, F4 = 0.02, F5 = 0.00[1] (Sidak post hoc)	Hedges' g reported only in supplementary Table 4 (not available)	Yes (interaction at F4 and F5)	Unfavourable	Table p values reconstructed from a column-wrapped PDF; digits in [ ] were displaced in the text layer and should be checked against the typeset table.

Study (first author, year) [ref]	Group / condition	Outcome	Time point	Pre value (mean ± SD)	Post value (mean ± SD)	Change (absolute and/or %)	Test statistic and p value	Effect size (95% CI if reported)	Statistically significant (Yes / No)	Direction (Favourable / Unfavourable / Null)	Notes
Isacco et al., 2020 [39]	WS (weight stable; n = 10)	POMS – Confusion	After each of 5 simulated fights (F1–F5, 30-min rest); T0 assessed but not tabulated	T0 NR; F1 = 39 ± 6	F5 = 37 ± 3 (F2 40 ± 6; F3 39 ± 7; F4 38 ± 3)	not reported	Comparator for the interaction (see WL row)	see WL row	n/a (comparator)	n/a (comparator)	—
Isacco et al., 2020 [39]	WL (weight loss ≥3% BM in the week before; n = 10)	Handgrip, right hand (HG right) (kg)	After each of 5 simulated fights (F1–F5, 30-min rest); T0 assessed but not tabulated	T0 NR; F1 = 53 ± 6	F5 = 49 ± 7 (F2 54 ± 7; F3 52 ± 7; F4 51 ± 7)	not reported	Random-effects model on AUC; time effect reported (see notes); group effect p = 0.26; time × group interaction p: F2 = 0.58, F3 = 0.25, F4 = 0.08, F5 = 0.05 (Sidak post hoc)	Hedges' g reported only in supplementary Table 4 (not available)	Borderline (interaction p = 0.05 at F5; reported by authors as significant)	Unclear	Table p values reconstructed from a column-wrapped PDF; digits in [ ] were displaced in the text layer and should be checked against the typeset table. p = 0.05 does not meet p < 0.05. Authors state that WL showed a smaller decrease in physical performance than WS.
Isacco et al., 2020 [39]	WS (weight stable; n = 10)	Handgrip, right hand (HG right) (kg)	After each of 5 simulated fights (F1–F5, 30-min rest); T0 assessed but not tabulated	T0 NR; F1 = 54 ± 9	F5 = 49 ± 9 (F2 52 ± 9; F3 50 ± 8; F4 50 ± 8)	not reported	Comparator for the interaction (see WL row)	see WL row	n/a (comparator)	n/a (comparator)	—
Isacco et al., 2020 [39]	WL (weight loss ≥3% BM in the week before; n = 10)	Handgrip, left hand (HG left) (kg)	After each of 5 simulated fights (F1–F5, 30-min rest); T0 assessed but not tabulated	T0 NR; F1 = 51 ± 7	F5 = 48 ± 8 (F2 50 ± 7; F3 50 ± 8; F4 50 ± 8)	not reported	Random-effects model on AUC; time effect reported (see notes); group effect p = 0.23; time × group interaction p: F2 = 0.57, F3 = 0.26, F4 = 0.08, F5 = 0.04 (Sidak post hoc)	Hedges' g reported only in supplementary Table 4 (not available)	Yes (interaction at F5)	Favourable (smaller decline in WL than WS)	Table p values reconstructed from a column-wrapped PDF; digits in [ ] were displaced in the text layer and should be checked against the typeset table. Direction per authors' discussion ('WL experienced a smaller decrease in physical performance').

Study (first author, year) [ref]	Group / condition	Outcome	Time point	Pre value (mean ± SD)	Post value (mean ± SD)	Change (absolute and/or %)	Test statistic and p value	Effect size (95% CI if reported)	Statistically significant (Yes / No)	Direction (Favourable / Unfavourable / Null)	Notes
Isacco et al., 2020 [39]	WS (weight stable; n = 10)	Handgrip, left hand (HG left) (kg)	After each of 5 simulated fights (F1–F5, 30-min rest); T0 assessed but not tabulated	T0 NR; F1 = 50 ± 8	F5 = 46 ± 7 (F2 50 ± 9; F3 48 ± 6; F4 46 ± 8)	not reported	Comparator for the interaction (see WL row)	see WL row	n/a (comparator)	n/a (comparator)	—
Isacco et al., 2020 [39]	WL (weight loss ≥3% BM in the week before; n = 10)	30-s horizontal isometric rowing – maximal strength (Fmax) (kg)	After each of 5 simulated fights (F1–F5, 30-min rest); T0 assessed but not tabulated	T0 NR; F1 = 80 ± 8	F5 = 79 ± 13 (F2 83 ± 14; F3 85 ± 13; F4 78 ± 13)	not reported	Random-effects model on AUC; time effect reported (see notes); group effect p = 0.16; time × group interaction p: F2 = 0.55, F3 = 0.18, F4 = 0.04[2], F5 = 0.01[3] (Sidak post hoc)	Hedges' g reported only in supplementary Table 4 (not available)	Yes (interaction at F4 and F5)	Favourable (Fmax preserved in WL vs decline in WS)	Table p values reconstructed from a column-wrapped PDF; digits in [ ] were displaced in the text layer and should be checked against the typeset table.
Isacco et al., 2020 [39]	WS (weight stable; n = 10)	30-s horizontal isometric rowing – maximal strength (Fmax) (kg)	After each of 5 simulated fights (F1–F5, 30-min rest); T0 assessed but not tabulated	T0 NR; F1 = 80 ± 16	F5 = 67 ± 15 (F2 76 ± 17; F3 76 ± 14; F4 70 ± 13)	not reported	Comparator for the interaction (see WL row)	see WL row	n/a (comparator)	n/a (comparator)	—
Isacco et al., 2020 [39]	WL (weight loss ≥3% BM in the week before; n = 10)	30-s horizontal isometric rowing – time sustained at Fmax (Time to Fmax) (s)	After each of 5 simulated fights (F1–F5, 30-min rest); T0 assessed but not tabulated	T0 NR; F1 = 11.5 ± 8.6	F5 = 9.8 ± 7.4 (F2 7.1 ± 5.9; F3 9.6 ± 7.1; F4 10.4 ± 9)	not reported	Random-effects model on AUC; time effect reported (see notes); group effect p = 0.001; time × group interaction p: F2 = 0.14, F3 = 0.00[7], F4 = 0.00, F5 = 0.00 (Sidak post hoc)	Hedges' g reported only in supplementary Table 4 (not available)	Yes (group effect; interaction at F3, F4, F5)	Favourable (longer in WL)	Table p values reconstructed from a column-wrapped PDF; digits in [ ] were displaced in the text layer and should be checked against the typeset table. Text gives F4 p < 0.001, F5 p < 0.01; table p values printed as 0.00. Group effect p = 0.001 not mentioned in text.

Study (first author, year) [ref]	Group / condition	Outcome	Time point	Pre value (mean ± SD)	Post value (mean ± SD)	Change (absolute and/or %)	Test statistic and p value	Effect size (95% CI if reported)	Statistically significant (Yes / No)	Direction (Favourable / Unfavourable / Null)	Notes
Isacco et al., 2020 [39]	WS (weight stable; n = 10)	30-s horizontal isometric rowing – time sustained at Fmax (Time to Fmax) (s)	After each of 5 simulated fights (F1–F5, 30-min rest); T0 assessed but not tabulated	T0 NR; F1 = 3.5 ± 3.5	F5 = 3.6 ± 2.7 (F2 2.8 ± 2.5; F3 4.3 ± 3.4; F4 4.4 ± 5.7)	not reported	Comparator for the interaction (see WL row)	see WL row	n/a (comparator)	n/a (comparator)	—
Isacco et al., 2020 [39]	WL (weight loss ≥3% BM in the week before; n = 10)	HRmin during fights (bpm)	After each of 5 simulated fights (F1–F5, 30-min rest); T0 assessed but not tabulated	T0 NR; F1 = 155 ± 24	F5 = 160 ± 9 (F2 161 ± 8; F3 158 ± 11; F4 154 ± 14)	not reported	Random-effects model on AUC; time effect reported (see notes); group effect p = 0.46; time × group interaction p: F2 = 0.18, F3 = 0.06, F4 = 0.12, F5 = 0.05 (Sidak post hoc)	Hedges' g reported only in supplementary Table 4 (not available)	Borderline (p = 0.05 at F5)	Unclear	Table p values reconstructed from a column-wrapped PDF; digits in [ ] were displaced in the text layer and should be checked against the typeset table. No time effect (WL p = 0.23; WS p = 0.15).
Isacco et al., 2020 [39]	WS (weight stable; n = 10)	HRmin during fights (bpm)	After each of 5 simulated fights (F1–F5, 30-min rest); T0 assessed but not tabulated	T0 NR; F1 = 163 ± 11	F5 = 158 ± 14 (F2 163 ± 8; F3 144 ± 52; F4 156 ± 11)	not reported	Comparator for the interaction (see WL row)	see WL row	n/a (comparator)	n/a (comparator)	—
Isacco et al., 2020 [39]	WL (weight loss ≥3% BM in the week before; n = 10)	HRmean during fights (bpm)	After each of 5 simulated fights (F1–F5, 30-min rest); T0 assessed but not tabulated	T0 NR; F1 = 17 ± 12 [sic]	F5 = 174 ± 6 (F2 174 ± 5; F3 173 ± 9; F4 170 ± 10)	not reported	Random-effects model on AUC; time effect reported (see notes); group effect p = 0.21; time × group interaction p: F2 = 0.25, F3 = 0.3, F4 = 0.59, F5 = 0.05 (Sidak post hoc)	Hedges' g reported only in supplementary Table 4 (not available)	Borderline (p = 0.05 at F5)	Unclear	Table p values reconstructed from a column-wrapped PDF; digits in [ ] were displaced in the text layer and should be checked against the typeset table. WL F1 value printed as '17 ± 12' (typographical error in original). No time effect (WL p = 0.32; WS p = 0.42).

Study (first author, year) [ref]	Group / condition	Outcome	Time point	Pre value (mean ± SD)	Post value (mean ± SD)	Change (absolute and/or %)	Test statistic and p value	Effect size (95% CI if reported)	Statistically significant (Yes / No)	Direction (Favourable / Unfavourable / Null)	Notes
Isacco et al., 2020 [39]	WS (weight stable; n = 10)	HRmean during fights (bpm)	After each of 5 simulated fights (F1–F5, 30-min rest); T0 assessed but not tabulated	T0 NR; F1 = 178 ± 6	F5 = 174 ± 9 (F2 177 ± 3; F3 176 ± 5; F4 175 ± 7)	not reported	Comparator for the interaction (see WL row)	see WL row	n/a (comparator)	n/a (comparator)	—
Isacco et al., 2020 [39]	WL (weight loss ≥3% BM in the week before; n = 10)	HRmax during fights (bpm)	After each of 5 simulated fights (F1–F5, 30-min rest); T0 assessed but not tabulated	T0 NR; F1 = 184 ± 10	F5 = 185 ± 6 (F2 184 ± 6; F3 184 ± 10; F4 184 ± 9)	not reported	Random-effects model on AUC; time effect reported (see notes); group effect p = 0.3[2]; time × group interaction p: F2 = 0.5, F3 = 0.56, F4 = 0.78, F5 = 0.07 (Sidak post hoc)	Hedges' g reported only in supplementary Table 4 (not available)	No (tendency p = 0.07 at F5)	Null	Table p values reconstructed from a column-wrapped PDF; digits in [ ] were displaced in the text layer and should be checked against the typeset table. No time effect (WL p = 0.89; WS p = 0.47).
Isacco et al., 2020 [39]	WS (weight stable; n = 10)	HRmax during fights (bpm)	After each of 5 simulated fights (F1–F5, 30-min rest); T0 assessed but not tabulated	T0 NR; F1 = 189 ± 8	F5 = 185 ± 9 (F2 187 ± 3; F3 187 ± 5; F4 187 ± 6)	not reported	Comparator for the interaction (see WL row)	see WL row	n/a (comparator)	n/a (comparator)	—
Isacco et al., 2020 [39]	WL (weight loss ≥3% BM in the week before; n = 10)	Relative cardiac cost (RCC) (%)	After each of 5 simulated fights (F1–F5, 30-min rest); T0 assessed but not tabulated	T0 NR; F1 = 82 ± 8	F5 = 85 ± 3 (F2 84 ± 3; F3 83 ± 5; F4 82 ± 6)	not reported	Random-effects model on AUC; time effect reported (see notes); group effect p = 0.12; time × group interaction p: F2 = 0.35, F3 = 0.11, F4 = 0.04, F5 = 0.09 (Sidak post hoc)	Hedges' g reported only in supplementary Table 4 (not available)	Yes (interaction at F4 only)	Unclear	Table p values reconstructed from a column-wrapped PDF; digits in [ ] were displaced in the text layer and should be checked against the typeset table. Time effect p < 0.001 in both groups.
Isacco et al., 2020 [39]	WS (weight stable; n = 10)	Relative cardiac cost (RCC) (%)	After each of 5 simulated fights (F1–F5, 30-min rest); T0 assessed but not tabulated	T0 NR; F1 = 86 ± 5	F5 = 83 ± 7 (F2 85 ± 2; F3 84 ± 4; F4 84 ± 6)	not reported	Comparator for the interaction (see WL row)	see WL row	n/a (comparator)	n/a (comparator)	—

Study (first author, year) [ref]	Group / condition	Outcome	Time point	Pre value (mean ± SD)	Post value (mean ± SD)	Change (absolute and/or %)	Test statistic and p value	Effect size (95% CI if reported)	Statistically significant (Yes / No)	Direction (Favourable / Unfavourable / Null)	Notes
Isacco et al., 2020 [39]	WL (weight loss ≥3% BM in the week before; n = 10)	Rate of perceived exertion (RPE, Borg 6–20)	After each of 5 simulated fights (F1–F5, 30-min rest); T0 assessed but not tabulated	not reported	Figure only	not reported	Group effect $p < 0.05$ (WL > WS); time effect $p < 0.001$; interaction F4 $p < 0.05$, F5 $p < 0.0001$	see supplementary Table 4 (not available)	Yes (group effect; interaction at F4 and F5)	Unfavourable	Data only in Figure 1A.
Isacco et al., 2020 [39]	WL (weight loss ≥3% BM in the week before; n = 10)	Workload (fight duration × RPE)	After each of 5 simulated fights (F1–F5, 30-min rest); T0 assessed but not tabulated	not reported	Figure only	not reported	Group effect $p < 0.05$ (WL > WS); time effect $p < 0.001$; interaction F5 $p < 0.001$	see supplementary Table 4 (not available)	Yes (group effect; interaction at F5)	Unfavourable	Data only in Figure 1B.
Mendes et al., 2013 [40]	WC group (experienced weight cyclers; RWL ≤5% BM in 5 d; n = 10)	8 × 15 s all-out arm-crank test – mean power output (W)	Pre-weight loss (baseline) vs post-weight loss (after simulated weigh-in + 4 h ad libitum refeeding/rehydration)	Figure only	Figure only	not reported	'No effects of RWL were shown on mean power, peak power or total work carried out in both groups'; no between-group differences (statistics and p NR)	not reported	No	Null	BOTH groups performed RWL (WC: 77.8 → 73.8 kg; non-WC: 73.5 → 69.5 kg); there is no weight-stable control group. Group allocation by weight-cycling history (non-randomised). 21 enrolled, 18 analysed (3 non-WC drop-outs). Mixed models for repeated measures. Values shown only in Figure 3 (not extracted from graph); SD, F and p values not reported in text.

Study (first author, year) [ref]	Group / condition	Outcome	Time point	Pre value (mean ± SD)	Post value (mean ± SD)	Change (absolute and/or %)	Test statistic and p value	Effect size (95% CI if reported)	Statistically significant (Yes / No)	Direction (Favourable / Unfavourable / Null)	Notes
Mendes et al., 2013 [40]	non-WC group (non-weight cyclers; RWL ≤5% BM in 5 d; n = 8)	8 × 15 s all-out arm-crank test – mean power output (W)	Pre-weight loss (baseline) vs post-weight loss (after simulated weigh-in + 4 h ad libitum refeeding/rehydration)	Figure only	Figure only	not reported	'No effects of RWL were shown on mean power, peak power or total work carried out in both groups'; no between-group differences (statistics and p NR)	not reported	No	Null	BOTH groups performed RWL (WC: 77.8 → 73.8 kg; non-WC: 73.5 → 69.5 kg); there is no weight-stable control group. Group allocation by weight-cycling history (non-randomised). 21 enrolled, 18 analysed (3 non-WC drop-outs). Mixed models for repeated measures. Values shown only in Figure 3 (not extracted from graph); SD, F and p values not reported in text.
Mendes et al., 2013 [40]	WC group (experienced weight cyclers; RWL ≤5% BM in 5 d; n = 10)	8 × 15 s all-out arm-crank test – peak power output (W)	Pre-weight loss (baseline) vs post-weight loss (after simulated weigh-in + 4 h ad libitum refeeding/rehydration)	Figure only	Figure only	not reported	'No effects of RWL were shown on mean power, peak power or total work carried out in both groups'; no between-group differences (statistics and p NR)	not reported	No	Null	BOTH groups performed RWL (WC: 77.8 → 73.8 kg; non-WC: 73.5 → 69.5 kg); there is no weight-stable control group. Group allocation by weight-cycling history (non-randomised). 21 enrolled, 18 analysed (3 non-WC drop-outs). Mixed models for repeated measures. Values shown only in Figure 3 (not extracted from graph); SD, F and p values not reported in text.

Study (first author, year) [ref]	Group / condition	Outcome	Time point	Pre value (mean ± SD)	Post value (mean ± SD)	Change (absolute and/or %)	Test statistic and p value	Effect size (95% CI if reported)	Statistically significant (Yes / No)	Direction (Favourable / Unfavourable / Null)	Notes
Mendes et al., 2013 [40]	non-WC group (non-weight cyclers; RWL ≤5% BM in 5 d; n = 8)	8 × 15 s all-out arm-crank test – peak power output (W)	Pre-weight loss (baseline) vs post-weight loss (after simulated weigh-in + 4 h ad libitum refeeding/rehydration)	Figure only	Figure only	not reported	'No effects of RWL were shown on mean power, peak power or total work carried out in both groups'; no between-group differences (statistics and p NR)	not reported	No	Null	BOTH groups performed RWL (WC: 77.8 → 73.8 kg; non-WC: 73.5 → 69.5 kg); there is no weight-stable control group. Group allocation by weight-cycling history (non-randomised). 21 enrolled, 18 analysed (3 non-WC drop-outs). Mixed models for repeated measures. Values shown only in Figure 3 (not extracted from graph); SD, F and p values not reported in text.
Mendes et al., 2013 [40]	WC group (experienced weight cyclers; RWL ≤5% BM in 5 d; n = 10)	8 × 15 s all-out arm-crank test – total work (kJ)	Pre-weight loss (baseline) vs post-weight loss (after simulated weigh-in + 4 h ad libitum refeeding/rehydration)	Figure only	Figure only	not reported	'No effects of RWL were shown on mean power, peak power or total work carried out in both groups'; no between-group differences (statistics and p NR)	not reported	No	Null	BOTH groups performed RWL (WC: 77.8 → 73.8 kg; non-WC: 73.5 → 69.5 kg); there is no weight-stable control group. Group allocation by weight-cycling history (non-randomised). 21 enrolled, 18 analysed (3 non-WC drop-outs). Mixed models for repeated measures. Values shown only in Figure 3 (not extracted from graph); SD, F and p values not reported in text.

Study (first author, year) [ref]	Group / condition	Outcome	Time point	Pre value (mean ± SD)	Post value (mean ± SD)	Change (absolute and/or %)	Test statistic and p value	Effect size (95% CI if reported)	Statistically significant (Yes / No)	Direction (Favourable / Unfavourable / Null)	Notes
Mendes et al., 2013 [40]	non-WC group (non-weight cyclers; RWL ≤5% BM in 5 d; n = 8)	8 × 15 s all-out arm-crank test – total work (kJ)	Pre-weight loss (baseline) vs post-weight loss (after simulated weigh-in + 4 h ad libitum refeeding/rehydration)	Figure only	Figure only	not reported	'No effects of RWL were shown on mean power, peak power or total work carried out in both groups'; no between-group differences (statistics and p NR)	not reported	No	Null	BOTH groups performed RWL (WC: 77.8 → 73.8 kg; non-WC: 73.5 → 69.5 kg); there is no weight-stable control group. Group allocation by weight-cycling history (non-randomised). 21 enrolled, 18 analysed (3 non-WC drop-outs). Mixed models for repeated measures. Values shown only in Figure 3 (not extracted from graph); SD, F and p values not reported in text.
Mendes et al., 2013 [40]	WC group (experienced weight cyclers; RWL ≤5% BM in 5 d; n = 10)	Second-by-second power output across the 8 bouts (W)	Pre-weight loss (baseline) vs post-weight loss (after simulated weigh-in + 4 h ad libitum refeeding/rehydration)	Figure only	Figure only	not reported	Descriptive (Figure 4); no statistics reported	not reported	not tested	Null	BOTH groups performed RWL (WC: 77.8 → 73.8 kg; non-WC: 73.5 → 69.5 kg); there is no weight-stable control group. Group allocation by weight-cycling history (non-randomised). 21 enrolled, 18 analysed (3 non-WC drop-outs). Mixed models for repeated measures. Data only in Figure 4.

Study (first author, year) [ref]	Group / condition	Outcome	Time point	Pre value (mean ± SD)	Post value (mean ± SD)	Change (absolute and/or %)	Test statistic and p value	Effect size (95% CI if reported)	Statistically significant (Yes / No)	Direction (Favourable / Unfavourable / Null)	Notes
Mendes et al., 2013 [40]	WC group (experienced weight cyclers; RWL ≤5% BM in 5 d; n = 10)	Plasma lactate at rest and 5 min post-exercise (mmol/L)	Pre-weight loss (baseline) vs post-weight loss (after simulated weigh-in + 4 h ad libitum refeeding/rehydration)	Figure only	Figure only	not reported	Main effect of time (rest vs post-exercise) $p < 0.001$; no effect of RWL and no group differences (p NR)	not reported	No (RWL effect)	Null	BOTH groups performed RWL (WC: 77.8 → 73.8 kg; non-WC: 73.5 → 69.5 kg); there is no weight-stable control group. Group allocation by weight-cycling history (non-randomised). 21 enrolled, 18 analysed (3 non-WC drop-outs). Mixed models for repeated measures. Physiological response to the test; data only in Figure 5.
Mendes et al., 2013 [40]	non-WC group (non-weight cyclers; RWL ≤5% BM in 5 d; n = 8)	Second-by-second power output across the 8 bouts (W)	Pre-weight loss (baseline) vs post-weight loss (after simulated weigh-in + 4 h ad libitum refeeding/rehydration)	Figure only	Figure only	not reported	Descriptive (Figure 4); no statistics reported	not reported	not tested	Null	BOTH groups performed RWL (WC: 77.8 → 73.8 kg; non-WC: 73.5 → 69.5 kg); there is no weight-stable control group. Group allocation by weight-cycling history (non-randomised). 21 enrolled, 18 analysed (3 non-WC drop-outs). Mixed models for repeated measures. Data only in Figure 4.

Study (first author, year) [ref]	Group / condition	Outcome	Time point	Pre value (mean ± SD)	Post value (mean ± SD)	Change (absolute and/or %)	Test statistic and p value	Effect size (95% CI if reported)	Statistically significant (Yes / No)	Direction (Favourable / Unfavourable / Null)	Notes
Mendes et al., 2013 [40]	non-WC group (non-weight cyclers; RWL ≤5% BM in 5 d; n = 8)	Plasma lactate at rest and 5 min post-exercise (mmol/L)	Pre-weight loss (baseline) vs post-weight loss (after simulated weigh-in + 4 h ad libitum refeeding/rehydration)	Figure only	Figure only	not reported	Main effect of time (rest vs post-exercise) $p < 0.001$; no effect of RWL and no group differences (p NR)	not reported	No (RWL effect)	Null	BOTH groups performed RWL (WC: 77.8 → 73.8 kg; non-WC: 73.5 → 69.5 kg); there is no weight-stable control group. Group allocation by weight-cycling history (non-randomised). 21 enrolled, 18 analysed (3 non-WC drop-outs). Mixed models for repeated measures. Physiological response to the test; data only in Figure 5.
Morales et al., 2018 [41]	RWL (>3% BM in 48 h before weigh-in; n = 16: 11 M, 5 F)	Balance – area of 95% ellipse (AE), eyes open (mm²)	Pre (1 wk before weigh-in) vs post (immediately after official weigh-in)	not reported	not reported	not reported	Mixed ANOVA time (2) × group (3), Bonferroni post hoc. No interaction or main effect (F and p NR)	not reported	No	Null	Data only in Figure 1. Group allocation was based on the weight loss actually achieved (quasi-experimental). Abstract defines PWL as '<3%', whereas Methods state PWL athletes 'lost more than 3%' starting >1 wk before.
Morales et al., 2018 [41]	PWL (weight loss begun >1 wk before competition; n = 13: 9 M, 4 F)	Balance – area of 95% ellipse (AE), eyes open (mm²)	Pre (1 wk before weigh-in) vs post (immediately after official weigh-in)	not reported	not reported	not reported	Same ANOVA; no pre–post difference in this group (p NR)	not reported	No	Null	Values for PWL and CG shown only in figures (not extracted from graphs).
Morales et al., 2018 [41]	Control group (weight maintenance; n = 9: 6 M, 3 F)	Balance – area of 95% ellipse (AE), eyes open (mm²)	Pre (1 wk before weigh-in) vs post (immediately after official weigh-in)	not reported	not reported	not reported	Same ANOVA; no pre–post difference in this group (p NR)	not reported	No	Null	Values for PWL and CG shown only in figures (not extracted from graphs).

Study (first author, year) [ref]	Group / condition	Outcome	Time point	Pre value (mean ± SD)	Post value (mean ± SD)	Change (absolute and/or %)	Test statistic and p value	Effect size (95% CI if reported)	Statistically significant (Yes / No)	Direction (Favourable / Unfavourable / Null)	Notes
Morales et al., 2018 [41]	RWL (>3% BM in 48 h before weigh-in; n = 16: 11 M, 5 F)	Balance – area of 95% ellipse (AE), eyes closed (mm²)	Pre (1 wk before weigh-in) vs post (immediately after official weigh-in)	4.83 ± 0.87	6.31 ± 1.39	not reported	Mixed ANOVA time (2) × group (3), Bonferroni post hoc. Time × group interaction $F(2,30) = 5.108$, $p = 0.023$; post hoc pre vs post in RWL $p < 0.05$	not reported	Yes (interaction + post hoc within RWL)	Unfavourable	Higher AE = poorer balance. RWL values from Abstract only. Balance variables ln(1+x)-transformed for analysis; unclear whether reported means are raw or transformed. Group allocation was based on the weight loss actually achieved (quasi-experimental). Abstract defines PWL as '<3%', whereas Methods state PWL athletes 'lost more than 3%' starting >1 wk before.
Morales et al., 2018 [41]	PWL (weight loss begun >1 wk before competition; n = 13: 9 M, 4 F)	Balance – area of 95% ellipse (AE), eyes closed (mm²)	Pre (1 wk before weigh-in) vs post (immediately after official weigh-in)	not reported	not reported	not reported	Same ANOVA; no pre–post difference in this group (p NR)	not reported	No (post hoc)	Null	Values for PWL and CG shown only in figures (not extracted from graphs).
Morales et al., 2018 [41]	Control group (weight maintenance; n = 9: 6 M, 3 F)	Balance – area of 95% ellipse (AE), eyes closed (mm²)	Pre (1 wk before weigh-in) vs post (immediately after official weigh-in)	not reported	not reported	not reported	Same ANOVA; no pre–post difference in this group (p NR)	not reported	No (post hoc)	Null	Values for PWL and CG shown only in figures (not extracted from graphs).

Study (first author, year) [ref]	Group / condition	Outcome	Time point	Pre value (mean ± SD)	Post value (mean ± SD)	Change (absolute and/or %)	Test statistic and p value	Effect size (95% CI if reported)	Statistically significant (Yes / No)	Direction (Favourable / Unfavourable / Null)	Notes
Morales et al., 2018 [41]	RWL (>3% BM in 48 h before weigh-in; n = 16: 11 M, 5 F)	Balance – mean anteroposterior velocity (MAPV), eyes open ($mm \cdot s^{-1}$)	Pre (1 wk before weigh-in) vs post (immediately after official weigh-in)	2.25 ± 0.20	2.51 ± 0.32	not reported	Mixed ANOVA time (2) × group (3), Bonferroni post hoc. Main effect $F(2,30) = 4.299$, $p = 0.041$ (factor not specified; no interaction reported); post hoc pre vs post in RWL $p < 0.05$	not reported	No (interaction) / Yes (post hoc within RWL)	Unfavourable	Only a 'main effect' was reported (not an interaction); the post hoc within-RWL difference is what supports the finding. RWL values from Abstract only. Group allocation was based on the weight loss actually achieved (quasi-experimental). Abstract defines PWL as '<3%', whereas Methods state PWL athletes 'lost more than 3%' starting >1 wk before.
Morales et al., 2018 [41]	PWL (weight loss begun >1 wk before competition; n = 13: 9 M, 4 F)	Balance – mean anteroposterior velocity (MAPV), eyes open ($mm \cdot s^{-1}$)	Pre (1 wk before weigh-in) vs post (immediately after official weigh-in)	not reported	not reported	not reported	Same ANOVA; no pre–post difference in this group (p NR)	not reported	No (post hoc)	Null	Values for PWL and CG shown only in figures (not extracted from graphs).
Morales et al., 2018 [41]	Control group (weight maintenance; n = 9: 6 M, 3 F)	Balance – mean anteroposterior velocity (MAPV), eyes open ($mm \cdot s^{-1}$)	Pre (1 wk before weigh-in) vs post (immediately after official weigh-in)	not reported	not reported	not reported	Same ANOVA; no pre–post difference in this group (p NR)	not reported	No (post hoc)	Null	Values for PWL and CG shown only in figures (not extracted from graphs).
Morales et al., 2018 [41]	RWL (>3% BM in 48 h before weigh-in; n = 16: 11 M, 5 F)	Balance – mean anteroposterior velocity (MAPV), eyes closed ($mm \cdot s^{-1}$)	Pre (1 wk before weigh-in) vs post (immediately after official weigh-in)	2.44 ± 0.26	3.06 ± 0.56	not reported	Mixed ANOVA time (2) × group (3), Bonferroni post hoc. Time × group interaction $F(2,30) = 3.403$, $p = 0.042$; post hoc RWL $p < 0.05$	not reported	Yes (interaction + post hoc within RWL)	Unfavourable	RWL values from Abstract only. Group allocation was based on the weight loss actually achieved (quasi-experimental). Abstract defines PWL as '<3%', whereas Methods state PWL athletes 'lost more than 3%' starting >1 wk before.

Study (first author, year) [ref]	Group / condition	Outcome	Time point	Pre value (mean ± SD)	Post value (mean ± SD)	Change (absolute and/or %)	Test statistic and p value	Effect size (95% CI if reported)	Statistically significant (Yes / No)	Direction (Favourable / Unfavourable / Null)	Notes
Morales et al., 2018 [41]	PWL (weight loss begun >1 wk before competition; n = 13: 9 M, 4 F)	Balance – mean anteroposterior velocity (MAPV), eyes closed (mm·s⁻¹)	Pre (1 wk before weigh-in) vs post (immediately after official weigh-in)	not reported	not reported	not reported	Same ANOVA; no pre–post difference in this group (p NR)	not reported	No (post hoc)	Null	Values for PWL and CG shown only in figures (not extracted from graphs).
Morales et al., 2018 [41]	Control group (weight maintenance; n = 9: 6 M, 3 F)	Balance – mean anteroposterior velocity (MAPV), eyes closed (mm·s⁻¹)	Pre (1 wk before weigh-in) vs post (immediately after official weigh-in)	not reported	not reported	not reported	Same ANOVA; no pre–post difference in this group (p NR)	not reported	No (post hoc)	Null	Values for PWL and CG shown only in figures (not extracted from graphs).
Morales et al., 2018 [41]	RWL (>3% BM in 48 h before weigh-in; n = 16: 11 M, 5 F)	Balance – mean mediolateral velocity (MMLV), eyes open (mm·s⁻¹)	Pre (1 wk before weigh-in) vs post (immediately after official weigh-in)	not reported	not reported	not reported	Mixed ANOVA time (2) × group (3), Bonferroni post hoc. No interaction or main effect (F and p NR)	not reported	No	Null	Data only in Figure 1. Group allocation was based on the weight loss actually achieved (quasi-experimental). Abstract defines PWL as '<3%', whereas Methods state PWL athletes 'lost more than 3%' starting >1 wk before.
Morales et al., 2018 [41]	PWL (weight loss begun >1 wk before competition; n = 13: 9 M, 4 F)	Balance – mean mediolateral velocity (MMLV), eyes open (mm·s⁻¹)	Pre (1 wk before weigh-in) vs post (immediately after official weigh-in)	not reported	not reported	not reported	Same ANOVA; no pre–post difference in this group (p NR)	not reported	No	Null	Values for PWL and CG shown only in figures (not extracted from graphs).
Morales et al., 2018 [41]	Control group (weight maintenance; n = 9: 6 M, 3 F)	Balance – mean mediolateral velocity (MMLV), eyes open (mm·s⁻¹)	Pre (1 wk before weigh-in) vs post (immediately after official weigh-in)	not reported	not reported	not reported	Same ANOVA; no pre–post difference in this group (p NR)	not reported	No	Null	Values for PWL and CG shown only in figures (not extracted from graphs).

Study (first author, year) [ref]	Group / condition	Outcome	Time point	Pre value (mean ± SD)	Post value (mean ± SD)	Change (absolute and/or %)	Test statistic and p value	Effect size (95% CI if reported)	Statistically significant (Yes / No)	Direction (Favourable / Unfavourable / Null)	Notes
Morales et al., 2018 [41]	RWL (>3% BM in 48 h before weigh-in; n = 16: 11 M, 5 F)	Balance – mean mediolateral velocity (MMLV), eyes closed ($mm \cdot s^{-1}$)	Pre (1 wk before weigh-in) vs post (immediately after official weigh-in)	2.07 ± 0.2	2.52 ± 0.45	not reported	Mixed ANOVA time (2) × group (3), Bonferroni post hoc. Time × group interaction $F(2,30) = 4.140$, $p = 0.027$; post hoc RWL $p < 0.05$	not reported	Yes (interaction + post hoc within RWL)	Unfavourable	RWL values from Abstract only. Group allocation was based on the weight loss actually achieved (quasi-experimental). Abstract defines PWL as '<3%', whereas Methods state PWL athletes 'lost more than 3%' starting >1 wk before.
Morales et al., 2018 [41]	PWL (weight loss begun >1 wk before competition; n = 13: 9 M, 4 F)	Balance – mean mediolateral velocity (MMLV), eyes closed ($mm \cdot s^{-1}$)	Pre (1 wk before weigh-in) vs post (immediately after official weigh-in)	not reported	not reported	not reported	Same ANOVA; no pre–post difference in this group (p NR)	not reported	No (post hoc)	Null	Values for PWL and CG shown only in figures (not extracted from graphs).
Morales et al., 2018 [41]	Control group (weight maintenance; n = 9: 6 M, 3 F)	Balance – mean mediolateral velocity (MMLV), eyes closed ($mm \cdot s^{-1}$)	Pre (1 wk before weigh-in) vs post (immediately after official weigh-in)	not reported	not reported	not reported	Same ANOVA; no pre–post difference in this group (p NR)	not reported	No (post hoc)	Null	Values for PWL and CG shown only in figures (not extracted from graphs).
Morales et al., 2018 [41]	RWL (>3% BM in 48 h before weigh-in; n = 16: 11 M, 5 F)	Simple reaction time (s)	Pre (1 wk before weigh-in) vs post (immediately after official weigh-in)	0.38 ± 0.04	0.42 ± 0.06	not reported	Mixed ANOVA time (2) × group (3), Bonferroni post hoc. Main effect $F(2,30) = 7.57$, $p = 0.011$; no significant interaction in the multivariate contrast; post hoc pre vs post $p < 0.05$ (text does not name the group; Abstract attributes it to RWL)	not reported	No (interaction) / Yes (main effect + post hoc)	Unfavourable	Main effect without interaction: the group-specific attribution rests on post hoc comparisons (Figure 2). Longer RT = poorer performance. Group allocation was based on the weight loss actually achieved (quasi-experimental). Abstract defines PWL as '<3%', whereas Methods state PWL athletes 'lost more than 3%' starting >1 wk before.

Study (first author, year) [ref]	Group / condition	Outcome	Time point	Pre value (mean ± SD)	Post value (mean ± SD)	Change (absolute and/or %)	Test statistic and p value	Effect size (95% CI if reported)	Statistically significant (Yes / No)	Direction (Favourable / Unfavourable / Null)	Notes
Morales et al., 2018 [41]	PWL (weight loss begun >1 wk before competition; n = 13: 9 M, 4 F)	Simple reaction time (s)	Pre (1 wk before weigh-in) vs post (immediately after official weigh-in)	not reported	not reported	not reported	Same ANOVA; no pre–post difference in this group (p NR)	not reported	No (post hoc)	Null	Values for PWL and CG shown only in figures (not extracted from graphs).
Morales et al., 2018 [41]	Control group (weight maintenance; n = 9: 6 M, 3 F)	Simple reaction time (s)	Pre (1 wk before weigh-in) vs post (immediately after official weigh-in)	not reported	not reported	not reported	Same ANOVA; no pre–post difference in this group (p NR)	not reported	No (post hoc)	Null	Values for PWL and CG shown only in figures (not extracted from graphs).
Morales et al., 2018 [41]	RWL (>3% BM in 48 h before weigh-in; n = 16: 11 M, 5 F)	Isometric hand grip strength (IGS) (kg)	Pre (1 wk before weigh-in) vs post (immediately after official weigh-in)	not reported	not reported	not reported	Mixed ANOVA time (2) × group (3), Bonferroni post hoc. No significant interaction or main effect (F and p NR)	not reported	No	Null	Data only in Figure 3 (text refers to a 'Figure 4' that does not exist). Hand not specified. Group allocation was based on the weight loss actually achieved (quasi-experimental). Abstract defines PWL as '<3%', whereas Methods state PWL athletes 'lost more than 3%' starting >1 wk before.
Morales et al., 2018 [41]	PWL (weight loss begun >1 wk before competition; n = 13: 9 M, 4 F)	Isometric hand grip strength (IGS) (kg)	Pre (1 wk before weigh-in) vs post (immediately after official weigh-in)	not reported	not reported	not reported	Same ANOVA; no pre–post difference in this group (p NR)	not reported	No	Null	Values for PWL and CG shown only in figures (not extracted from graphs).
Morales et al., 2018 [41]	Control group (weight maintenance; n = 9: 6 M, 3 F)	Isometric hand grip strength (IGS) (kg)	Pre (1 wk before weigh-in) vs post (immediately after official weigh-in)	not reported	not reported	not reported	Same ANOVA; no pre–post difference in this group (p NR)	not reported	No	Null	Values for PWL and CG shown only in figures (not extracted from graphs).

Study (first author, year) [ref]	Group / condition	Outcome	Time point	Pre value (mean ± SD)	Post value (mean ± SD)	Change (absolute and/or %)	Test statistic and p value	Effect size (95% CI if reported)	Statistically significant (Yes / No)	Direction (Favourable / Unfavourable / Null)	Notes
Morales et al., 2018 [41]	RWL (>3% BM in 48 h before weigh-in; n = 16: 11 M, 5 F)	Isometric trunk traction strength (ITTS) (kg)	Pre (1 wk before weigh-in) vs post (immediately after official weigh-in)	not reported	not reported	not reported	Mixed ANOVA time (2) × group (3), Bonferroni post hoc. No significant interaction or main effect (F and p NR)	not reported	No	Null	Data only in Figure 3. Group allocation was based on the weight loss actually achieved (quasi-experimental). Abstract defines PWL as '<3%', whereas Methods state PWL athletes 'lost more than 3%' starting >1 wk before.
Morales et al., 2018 [41]	PWL (weight loss begun >1 wk before competition; n = 13: 9 M, 4 F)	Isometric trunk traction strength (ITTS) (kg)	Pre (1 wk before weigh-in) vs post (immediately after official weigh-in)	not reported	not reported	not reported	Same ANOVA; no pre–post difference in this group (p NR)	not reported	No	Null	Values for PWL and CG shown only in figures (not extracted from graphs).
Morales et al., 2018 [41]	Control group (weight maintenance; n = 9: 6 M, 3 F)	Isometric trunk traction strength (ITTS) (kg)	Pre (1 wk before weigh-in) vs post (immediately after official weigh-in)	not reported	not reported	not reported	Same ANOVA; no pre–post difference in this group (p NR)	not reported	No	Null	Values for PWL and CG shown only in figures (not extracted from graphs).
Reljic et al., 2016 [42]	WLG (weight loss group; RBML 5–7 d before competition; n = 14)	Peak running velocity, incremental treadmill test (km/h)	t-1 (ordinary training) vs t-2 (1–2 d before competition, after RBML); t-3 (post-competition) in notes	16.2 ± 1.3	15.9 ± 1.5	not reported	Two-way RM ANOVA (group × time; Sidak post hoc) or Friedman/Wilcoxon when non-normal. No significant effect (Friedman test; statistic and p NR)	not reported	No	Null	t-3 value: 16.2 ± 1.6. Groups did not differ at any time point. Non-randomised allocation (athletes who habitually cut weight vs those who did not).
Reljic et al., 2016 [42]	CON (control group, no body mass loss; n = 14)	Peak running velocity, incremental treadmill test (km/h)	t-1 (ordinary training) vs t-2 (1–2 d before competition, after RBML); t-3 (post-competition) in notes	16.1 ± 1.1	16.1 ± 1.0	not reported	No significant changes in CON for any performance parameter (p NR)	not reported	No	Null	t-3 value: 15.9 ± 1.1.

Study (first author, year) [ref]	Group / condition	Outcome	Time point	Pre value (mean ± SD)	Post value (mean ± SD)	Change (absolute and/or %)	Test statistic and p value	Effect size (95% CI if reported)	Statistically significant (Yes / No)	Direction (Favourable / Unfavourable / Null)	Notes
Reljic et al., 2016 [42]	WLG (weight loss group; RBML 5–7 d before competition; n = 14)	VO2peak, absolute (L/min)	t-1 (ordinary training) vs t-2 (1–2 d before competition, after RBML); t-3 (post-competition) in notes	4.42 ± 0.63	4.22 ± 0.72	not reported	Two-way RM ANOVA (group × time; Sidak post hoc) or Friedman/Wilcoxon when non-normal. Main time effect $p < 0.05$; post hoc: no significant change after RBML	$\eta p^2 = 0.14$ (time effect)	No (post hoc); main time effect Yes	Null	t-3 value: 4.35 ± 0.66. Groups did not differ at any time point. Non-randomised allocation (athletes who habitually cut weight vs those who did not). Change in VO2peak correlated with change in Hbmass ($r = 0.66$, $p < 0.001$).
Reljic et al., 2016 [42]	CON (control group, no body mass loss; n = 14)	VO2peak, absolute (L/min)	t-1 (ordinary training) vs t-2 (1–2 d before competition, after RBML); t-3 (post-competition) in notes	4.41 ± 0.60	4.36 ± 0.69	not reported	No significant changes in CON for any performance parameter (p NR)	not reported	No	Null	t-3 value: 4.22 ± 0.65.
Reljic et al., 2016 [42]	WLG (weight loss group; RBML 5–7 d before competition; n = 14)	VO2peak, relative (mL/kg/min)	t-1 (ordinary training) vs t-2 (1–2 d before competition, after RBML); t-3 (post-competition) in notes	57.9 ± 5.2	57.9 ± 6.5	not reported	Two-way RM ANOVA (group × time; Sidak post hoc) or Friedman/Wilcoxon when non-normal. 'Remained unchanged' (statistic and p NR)	not reported	No	Null	t-3 value: 58.2 ± 6.5. Groups did not differ at any time point. Non-randomised allocation (athletes who habitually cut weight vs those who did not).
Reljic et al., 2016 [42]	CON (control group, no body mass loss; n = 14)	VO2peak, relative (mL/kg/min)	t-1 (ordinary training) vs t-2 (1–2 d before competition, after RBML); t-3 (post-competition) in notes	56.8 ± 6.8	55.9 ± 5.7	not reported	No significant changes in CON for any performance parameter (p NR)	not reported	No	Null	t-3 value: 54.8 ± 6.4.

Study (first author, year) [ref]	Group / condition	Outcome	Time point	Pre value (mean ± SD)	Post value (mean ± SD)	Change (absolute and/or %)	Test statistic and p value	Effect size (95% CI if reported)	Statistically significant (Yes / No)	Direction (Favourable / Unfavourable / Null)	Notes
Reljic et al., 2016 [42]	WLG (weight loss group; RBML 5–7 d before competition; n = 14)	4LT performance – running velocity at 4 mmol/L blood lactate (km/h)	t-1 (ordinary training) vs t-2 (1–2 d before competition, after RBML); t-3 (post-competition) in notes	13.1 ± 1.2	13.7 ± 1.3	not reported	Two-way RM ANOVA (group × time; Sidak post hoc) or Friedman/Wilcoxon when non-normal. Main time effect $p < 0.05$; WLG t-2 vs t-1 $p < 0.05$ (post hoc); no interaction reported	$\eta p^2 = 0.16$ (time effect)	Yes (post hoc within WLG; main time effect)	Favourable (as reported; see notes)	t-3 value: 13.4 ± 1.6. Groups did not differ at any time point. Non-randomised allocation (athletes who habitually cut weight vs those who did not). Authors attribute the higher 4LT velocity to reduced carbohydrate stores / lower lactate production, not to improved aerobic capacity.
Reljic et al., 2016 [42]	CON (control group, no body mass loss; n = 14)	4LT performance – running velocity at 4 mmol/L blood lactate (km/h)	t-1 (ordinary training) vs t-2 (1–2 d before competition, after RBML); t-3 (post-competition) in notes	13.4 ± 1.7	13.6 ± 1.7	not reported	No significant changes in CON for any performance parameter (p NR)	not reported	No	Null	t-3 value: 13.8 ± 1.6.
Reljic et al., 2016 [42]	WLG (weight loss group; RBML 5–7 d before competition; n = 14)	ILT performance – individual lactate threshold (km/h)	t-1 (ordinary training) vs t-2 (1–2 d before competition, after RBML); t-3 (post-competition) in notes	11.7 ± 1.3	12.2 ± 1.2	not reported	Two-way RM ANOVA (group × time; Sidak post hoc) or Friedman/Wilcoxon when non-normal. No difference between trials (statistic and p NR)	not reported	No	Null	t-3 value: 11.9 ± 1.7. Groups did not differ at any time point. Non-randomised allocation (athletes who habitually cut weight vs those who did not).
Reljic et al., 2016 [42]	CON (control group, no body mass loss; n = 14)	ILT performance – individual lactate threshold (km/h)	t-1 (ordinary training) vs t-2 (1–2 d before competition, after RBML); t-3 (post-competition) in notes	12.1 ± 1.6	12.2 ± 1.6	not reported	No significant changes in CON for any performance parameter (p NR)	not reported	No	Null	t-3 value: 12.3 ± 1.5.

Study (first author, year) [ref]	Group / condition	Outcome	Time point	Pre value (mean ± SD)	Post value (mean ± SD)	Change (absolute and/or %)	Test statistic and p value	Effect size (95% CI if reported)	Statistically significant (Yes / No)	Direction (Favourable / Unfavourable / Null)	Notes
Reljic et al., 2016 [42]	WLG (weight loss group; RBML 5–7 d before competition; n = 14)	Heart rate peak (beats/min)	t-1 (ordinary training) vs t-2 (1–2 d before competition, after RBML); t-3 (post-competition) in notes	203 ± 7	201 ± 8	not reported	Two-way RM ANOVA (group × time; Sidak post hoc) or Friedman/Wilcoxon when non-normal. Main time effect $p < 0.001$; WLG t-3 < t-1 ($p < 0.01$) and t-3 < t-2 ($p < 0.05$); t-2 vs t-1 not significant	$\eta p^2 = 0.25$ (time effect)	No (t-2 vs t-1)	Null	t-3 value: 197 ± 6. Groups did not differ at any time point. Non-randomised allocation (athletes who habitually cut weight vs those who did not). Physiological response during the performance test. Significant differences involve t-3 only.
Reljic et al., 2016 [42]	CON (control group, no body mass loss; n = 14)	Heart rate peak (beats/min)	t-1 (ordinary training) vs t-2 (1–2 d before competition, after RBML); t-3 (post-competition) in notes	197 ± 9	197 ± 9	not reported	No significant changes in CON for any performance parameter (p NR)	not reported	No	Null	t-3 value: 196 ± 9.
Reljic et al., 2016 [42]	WLG (weight loss group; RBML 5–7 d before competition; n = 14)	Blood lactate peak (mmol/L)	t-1 (ordinary training) vs t-2 (1–2 d before competition, after RBML); t-3 (post-competition) in notes	10.9 ± 2.2	9.3 ± 2.2	not reported	Two-way RM ANOVA (group × time; Sidak post hoc) or Friedman/Wilcoxon when non-normal. Main time effect $p < 0.05$; WLG t-2 < t-1 and t-2 < t-3 ($p < 0.05$)	$\eta p^2 = 0.14$ (time effect)	Yes (post hoc within WLG)	Unclear (physiological marker)	t-3 value: 10.7 ± 2.2. Groups did not differ at any time point. Non-randomised allocation (athletes who habitually cut weight vs those who did not). Physiological response during the performance test; interpreted by authors as a sign of reduced carbohydrate stores.
Reljic et al., 2016 [42]	CON (control group, no body mass loss; n = 14)	Blood lactate peak (mmol/L)	t-1 (ordinary training) vs t-2 (1–2 d before competition, after RBML); t-3 (post-competition) in notes	10.3 ± 2.2	10.3 ± 2.7	not reported	No significant changes in CON for any performance parameter (p NR)	not reported	No	Null	t-3 value: 9.4 ± 2.7.

Study (first author, year) [ref]	Group / condition	Outcome	Time point	Pre value (mean ± SD)	Post value (mean ± SD)	Change (absolute and/or %)	Test statistic and p value	Effect size (95% CI if reported)	Statistically significant (Yes / No)	Direction (Favourable / Unfavourable / Null)	Notes
Reljic et al., 2016 [42]	WLG (weight loss group; RBML 5–7 d before competition; n = 14)	RERpeak	t-1 (ordinary training) vs t-2 (1–2 d before competition, after RBML); t-3 (post-competition) in notes	1.16 ± 0.08	1.07 ± 0.07	not reported	Two-way RM ANOVA (group × time; Sidak post hoc) or Friedman/Wilcoxon when non-normal. Group × time interaction $p < 0.001$; main time effect $p < 0.001$; WLG t-2 < t-1 ($p < 0.01$) and < t-3 ($p < 0.001$)	$\eta p^2 = 0.29$ (interaction); 0.26 (time)	Yes (interaction + post hoc within WLG)	Unclear (physiological marker)	t-3 value: 1.18 ± 0.08. Groups did not differ at any time point. Non-randomised allocation (athletes who habitually cut weight vs those who did not). Physiological response during the performance test.
Reljic et al., 2016 [42]	CON (control group, no body mass loss; n = 14)	RERpeak	t-1 (ordinary training) vs t-2 (1–2 d before competition, after RBML); t-3 (post-competition) in notes	1.15 ± 0.06	1.15 ± 0.08	not reported	No significant changes in CON for any performance parameter (p NR)	not reported	No	Null	t-3 value: 1.13 ± 0.10.
Zubac et al., 2020 [43]	RWL condition (~3% BM in 3 d by food/fluid restriction; n = 9)	Brief MVC force of knee extensors (MVC1 pre-fatigue / MVC2 post-fatigue) (N)	Pre vs post a sustained 70% MVC fatiguing contraction (within each experimental session)	225 ± 37 (MVC1)	Figure 2A only (MVC2)	~12% lower MVC1 than control (text); MVC2 ~10% below MVC1 in both conditions	Condition main effect $p = 0.001$ ($\eta^2 = 0.71$); time main effect $p = 0.010$ ($\eta^2 = 0.62$); MVC2 < MVC1 $p = 0.003$; Fisher post hoc of interaction: greater MVC loss after RWL $p = 0.001$ ($\eta^2 = 0.31$)	Partial η^2 (see stat column)	Yes (condition main effect; interaction post hoc)	Unfavourable	Randomised counterbalanced crossover (≥2-wk washout); no rehydration before testing. 2-way RM GLM (condition × time) with Fisher's LSD post hoc; significance set at $p < 0.05$ for main effects but $p < 0.10$ for INTERACTION effects (symbol #). MVC2 values only in Figure 2A. Units reported as N in text and Nm in tables.
Zubac et al., 2020 [43]	Control condition (n = 9)	Brief MVC force of knee extensors (MVC1 / MVC2) (N)	Pre vs post a sustained 70% MVC fatiguing contraction (within each experimental session)	257 ± 43 (MVC1)	Figure 2A only (MVC2)	not reported	Reference condition (see RWL row)	see RWL row	n/a (reference)	n/a (reference)	—

Study (first author, year) [ref]	Group / condition	Outcome	Time point	Pre value (mean ± SD)	Post value (mean ± SD)	Change (absolute and/or %)	Test statistic and p value	Effect size (95% CI if reported)	Statistically significant (Yes / No)	Direction (Favourable / Unfavourable / Null)	Notes
Zubac et al., 2020 [43]	RWL and control (values not attributed per condition in text)	Voluntary activation (VA, interpolated twitch) (%)	Pre vs post a sustained 70% MVC fatiguing contraction (within each experimental session)	86.5 ± 5.8 / 85.8 ± 6.2	79.5 ± 8.0 / 80.4 ± 8.2	~7% VA deficit after RWL (text)	Time main effect $p = 0.011$ ($\eta^2 = 0.60$; 6/8 participants decreased); condition main effect $p = 0.058$ ($\eta^2 = 0.23$); Fisher post hoc of interaction: reduction for RWL vs control $p = 0.001$ ($\eta^2 = 0.19$)	Partial η^2 (see stat column)	No (condition main effect) / Yes (interaction post hoc, α = 0.10)	Unfavourable	Randomised counterbalanced crossover (≥2-wk washout); no rehydration before testing. 2-way RM GLM (condition × time) with Fisher's LSD post hoc; significance set at $p < 0.05$ for main effects but $p < 0.10$ for INTERACTION effects (symbol #). Ambiguity: the text gives two pre→post pairs (86.5→79.5 described under the time effect; 85.8→80.4 under the condition effect) without stating which pair belongs to which condition; '6/8 participants' although n = 9. Condition-specific values only in Figure 2B.
Zubac et al., 2020 [43]	RWL condition (~3% BM in 3 d by food/fluid restriction; n = 9)	Time to exhaustion during sustained 70% MVC (s)	During fatiguing protocol	not reported	68.8 ± 20.2	16 s shorter than control	Paired t-test vs control $p = 0.020$	not reported	Yes	Unfavourable	Randomised counterbalanced crossover (≥2-wk washout); no rehydration before testing. 2-way RM GLM (condition × time) with Fisher's LSD post hoc; significance set at $p < 0.05$ for main effects but $p < 0.10$ for INTERACTION effects (symbol #). Abstract rounds to 69 ± 20 vs 86 ± 34 s.
Zubac et al., 2020 [43]	Control condition (n = 9)	Time to exhaustion during sustained 70% MVC (s)	During fatiguing protocol	not reported	85.9 ± 34.8	not reported	Reference condition (see RWL row)	see RWL row	n/a (reference)	n/a (reference)	—

Study (first author, year) [ref]	Group / condition	Outcome	Time point	Pre value (mean ± SD)	Post value (mean ± SD)	Change (absolute and/or %)	Test statistic and p value	Effect size (95% CI if reported)	Statistically significant (Yes / No)	Direction (Favourable / Unfavourable / Null)	Notes
Zubac et al., 2020 [43]	RWL condition (~3% BM in 3 d by food/fluid restriction; n = 9)	Peak blood lactate after fatiguing protocol (mmol/L)	Baseline, immediately post and 5 min post fatigue	not reported	5.1 ± 2.0 (peak)	53% lower than control (text)	$p = 0.001$	$\eta^2 = 0.810$	Yes	Unclear (physiological marker)	Randomised counterbalanced crossover (≥2-wk washout); no rehydration before testing. 2-way RM GLM (condition × time) with Fisher's LSD post hoc; significance set at $p < 0.05$ for main effects but $p < 0.10$ for INTERACTION effects (symbol #). Physiological response; lactate unavailable for one participant in RWL. (5.1 vs 11.4 corresponds to ~55% lower; article states 53%.)
Zubac et al., 2020 [43]	Control condition (n = 9)	Peak blood lactate after fatiguing protocol (mmol/L)	Baseline, immediately post and 5 min post fatigue	not reported	11.4 ± 3.9 (peak)	not reported	Reference condition (see RWL row)	see RWL row	n/a (reference)	n/a (reference)	—
Zubac et al., 2020 [43]	RWL condition (~3% BM in 3 d by food/fluid restriction; n = 9)	Resting doublet (100 Hz) – peak force (Nm)	Pre vs post a sustained 70% MVC fatiguing contraction (within each experimental session)	74.4 ± 11.5	66.9 ± 16.5	not reported	Time main effect $p = 0.001$ ($\eta^2 = 0.72$); Fisher post hoc of interaction: greater decline in RWL $p = 0.050$	Partial η^2 where given	Yes (interaction post hoc, $\alpha = 0.10$)	Unfavourable	Randomised counterbalanced crossover (≥2-wk washout); no rehydration before testing. 2-way RM GLM (condition × time) with Fisher's LSD post hoc; significance set at $p < 0.05$ for main effects but $p < 0.10$ for INTERACTION effects (symbol #). Evoked contractile property.
Zubac et al., 2020 [43]	Control condition (n = 9)	Resting doublet (100 Hz) – peak force (Nm)	Pre vs post a sustained 70% MVC fatiguing contraction (within each experimental session)	79.3 ± 11.1	74.8 ± 9.9	not reported	Comparator (see RWL row)	see RWL row	n/a (comparator)	n/a (comparator)	—

Study (first author, year) [ref]	Group / condition	Outcome	Time point	Pre value (mean ± SD)	Post value (mean ± SD)	Change (absolute and/or %)	Test statistic and p value	Effect size (95% CI if reported)	Statistically significant (Yes / No)	Direction (Favourable / Unfavourable / Null)	Notes
Zubac et al., 2020 [43]	RWL condition (~3% BM in 3 d by food/fluid restriction; n = 9)	Resting doublet – contraction time, CT (ms)	Pre vs post a sustained 70% MVC fatiguing contraction (within each experimental session)	66.2 ± 13.0	75.5 ± 23.2	not reported	Time effect (* post hoc of time main effect, $p < 0.05$); interaction NR	Partial η^2 where given	No (interaction)	Null	Randomised counterbalanced crossover (≥2-wk washout); no rehydration before testing. 2-way RM GLM (condition × time) with Fisher's LSD post hoc; significance set at $p < 0.05$ for main effects but $p < 0.10$ for INTERACTION effects (symbol #). Evoked contractile property.
Zubac et al., 2020 [43]	Control condition (n = 9)	Resting doublet – contraction time, CT (ms)	Pre vs post a sustained 70% MVC fatiguing contraction (within each experimental session)	70.8 ± 17.7	74.8 ± 16.5	not reported	Comparator (see RWL row)	see RWL row	n/a (comparator)	n/a (comparator)	—
Zubac et al., 2020 [43]	RWL condition (~3% BM in 3 d by food/fluid restriction; n = 9)	Resting doublet – half-relaxation time, HRT (ms)	Pre vs post a sustained 70% MVC fatiguing contraction (within each experimental session)	85.0 ± 33.5	64.5 ± 24.9	not reported	Time effect (*); interaction $p = 0.887$	Partial η^2 where given	No (interaction)	Null	Randomised counterbalanced crossover (≥2-wk washout); no rehydration before testing. 2-way RM GLM (condition × time) with Fisher's LSD post hoc; significance set at $p < 0.05$ for main effects but $p < 0.10$ for INTERACTION effects (symbol #). Evoked contractile property.
Zubac et al., 2020 [43]	Control condition (n = 9)	Resting doublet – half-relaxation time, HRT (ms)	Pre vs post a sustained 70% MVC fatiguing contraction (within each experimental session)	105.6 ± 38.9	85.4 ± 32.2	not reported	Comparator (see RWL row)	see RWL row	n/a (comparator)	n/a (comparator)	—

Study (first author, year) [ref]	Group / condition	Outcome	Time point	Pre value (mean ± SD)	Post value (mean ± SD)	Change (absolute and/or %)	Test statistic and p value	Effect size (95% CI if reported)	Statistically significant (Yes / No)	Direction (Favourable / Unfavourable / Null)	Notes
Zubac et al., 2020 [43]	RWL condition (~3% BM in 3 d by food/fluid restriction; n = 9)	Resting doublet – time-to-end, TTE (ms)	Pre vs post a sustained 70% MVC fatiguing contraction (within each experimental session)	168.6 ± 66.0	124.6 ± 57.2	not reported	Time effect (*); interaction p = 0.989	Partial η² where given	No (interaction)	Null	Randomised counterbalanced crossover (≥2-wk washout); no rehydration before testing. 2-way RM GLM (condition × time) with Fisher's LSD post hoc; significance set at p < 0.05 for main effects but p < 0.10 for INTERACTION effects (symbol #). Evoked contractile property.
Zubac et al., 2020 [43]	Control condition (n = 9)	Resting doublet – time-to-end, TTE (ms)	Pre vs post a sustained 70% MVC fatiguing contraction (within each experimental session)	211.2 ± 77.8	159.6 ± 59.9	not reported	Comparator (see RWL row)	see RWL row	n/a (comparator)	n/a (comparator)	—
Zubac et al., 2020 [43]	RWL condition (~3% BM in 3 d by food/fluid restriction; n = 9)	Resting doublet – contractile rate of force development, RFD (N m/s)	Pre vs post a sustained 70% MVC fatiguing contraction (within each experimental session)	1175 ± 131	1086 ± 150	not reported	Time main effect p = 0.020 (η² = 0.71); interaction p = 0.323	Partial η² where given	No (interaction)	Null	Randomised counterbalanced crossover (≥2-wk washout); no rehydration before testing. 2-way RM GLM (condition × time) with Fisher's LSD post hoc; significance set at p < 0.05 for main effects but p < 0.10 for INTERACTION effects (symbol #). Evoked contractile property.
Zubac et al., 2020 [43]	Control condition (n = 9)	Resting doublet – contractile rate of force development, RFD (N m/s)	Pre vs post a sustained 70% MVC fatiguing contraction (within each experimental session)	1218 ± 122	1169 ± 178	not reported	Comparator (see RWL row)	see RWL row	n/a (comparator)	n/a (comparator)	—

Study (first author, year) [ref]	Group / condition	Outcome	Time point	Pre value (mean ± SD)	Post value (mean ± SD)	Change (absolute and/or %)	Test statistic and p value	Effect size (95% CI if reported)	Statistically significant (Yes / No)	Direction (Favourable / Unfavourable / Null)	Notes
Zubac et al., 2020 [43]	RWL condition (~3% BM in 3 d by food/fluid restriction; n = 9)	20 Hz tetanic train – force (Nm)	Pre vs post a sustained 70% MVC fatiguing contraction (within each experimental session)	158.1 ± 33.5	149.2 ± 18.5	not reported	Interaction $p = 0.331$ ($\eta^2 = 0.03$); no effect of RWL or fatigue	Partial η^2 where given	No	Null	Randomised counterbalanced crossover (≥2-wk washout); no rehydration before testing. 2-way RM GLM (condition × time) with Fisher's LSD post hoc; significance set at $p < 0.05$ for main effects but $p < 0.10$ for INTERACTION effects (symbol #). Evoked contractile property.
Zubac et al., 2020 [43]	Control condition (n = 9)	20 Hz tetanic train – force (Nm)	Pre vs post a sustained 70% MVC fatiguing contraction (within each experimental session)	171.1 ± 46.6	162.9 ± 54.1	not reported	Comparator (see RWL row)	see RWL row	n/a (comparator)	n/a (comparator)	—
Zubac et al., 2020 [43]	RWL condition (~3% BM in 3 d by food/fluid restriction; n = 9)	20 Hz tetanic train – contraction time, CT (ms)	Pre vs post a sustained 70% MVC fatiguing contraction (within each experimental session)	570.1 ± 274.6	674.8 ± 387.1	not reported	No significant effect reported	Partial η^2 where given	No	Null	Randomised counterbalanced crossover (≥2-wk washout); no rehydration before testing. 2-way RM GLM (condition × time) with Fisher's LSD post hoc; significance set at $p < 0.05$ for main effects but $p < 0.10$ for INTERACTION effects (symbol #). Evoked contractile property.
Zubac et al., 2020 [43]	Control condition (n = 9)	20 Hz tetanic train – contraction time, CT (ms)	Pre vs post a sustained 70% MVC fatiguing contraction (within each experimental session)	558.9 ± 195.3	646.2 ± 132.1	not reported	Comparator (see RWL row)	see RWL row	n/a (comparator)	n/a (comparator)	—

Study (first author, year) [ref]	Group / condition	Outcome	Time point	Pre value (mean ± SD)	Post value (mean ± SD)	Change (absolute and/or %)	Test statistic and p value	Effect size (95% CI if reported)	Statistically significant (Yes / No)	Direction (Favourable / Unfavourable / Null)	Notes
Zubac et al., 2020 [43]	RWL condition (~3% BM in 3 d by food/fluid restriction; n = 9)	20 Hz tetanic train – half-relaxation time, HRT (ms)	Pre vs post a sustained 70% MVC fatiguing contraction (within each experimental session)	177.0 ± 73.1	118.8 ± 69.2	not reported	Time main effect $p = 0.001$ ($\eta^2 = 0.53$); Fisher post hoc after fatigue following RWL $p = 0.023$	Partial η^2 where given	Yes (interaction post hoc, $\alpha = 0.10$)	Unclear (contractile property)	Randomised counterbalanced crossover (≥2-wk washout); no rehydration before testing. 2-way RM GLM (condition × time) with Fisher's LSD post hoc; significance set at $p < 0.05$ for main effects but $p < 0.10$ for INTERACTION effects (symbol #). Evoked contractile property.
Zubac et al., 2020 [43]	Control condition (n = 9)	20 Hz tetanic train – half-relaxation time, HRT (ms)	Pre vs post a sustained 70% MVC fatiguing contraction (within each experimental session)	223.9 ± 114.9	157.5 ± 82.4	not reported	Comparator (see RWL row)	see RWL row	n/a (comparator)	n/a (comparator)	—
Zubac et al., 2020 [43]	RWL condition (~3% BM in 3 d by food/fluid restriction; n = 9)	20 Hz tetanic train – time-to-end, TTE (ms)	Pre vs post a sustained 70% MVC fatiguing contraction (within each experimental session)	354.1 ± 146.2	237.4 ± 138.5	not reported	Time main effect $p = 0.002$ ($\eta^2 = 0.60$); Fisher post hoc after fatigue following RWL $p = 0.021$	Partial η^2 where given	Yes (interaction post hoc, $\alpha = 0.10$)	Unclear (contractile property)	Randomised counterbalanced crossover (≥2-wk washout); no rehydration before testing. 2-way RM GLM (condition × time) with Fisher's LSD post hoc; significance set at $p < 0.05$ for main effects but $p < 0.10$ for INTERACTION effects (symbol #). Evoked contractile property.
Zubac et al., 2020 [43]	Control condition (n = 9)	20 Hz tetanic train – time-to-end, TTE (ms)	Pre vs post a sustained 70% MVC fatiguing contraction (within each experimental session)	447.8 ± 229.8	315.4 ± 164.8	not reported	Comparator (see RWL row)	see RWL row	n/a (comparator)	n/a (comparator)	—

Study (first author, year) [ref]	Group / condition	Outcome	Time point	Pre value (mean ± SD)	Post value (mean ± SD)	Change (absolute and/or %)	Test statistic and p value	Effect size (95% CI if reported)	Statistically significant (Yes / No)	Direction (Favourable / Unfavourable / Null)	Notes
Zubac et al., 2020 [43]	RWL condition (~3% BM in 3 d by food/fluid restriction; n = 9)	20 Hz tetanic train – RFD (N m/s)	Pre vs post a sustained 70% MVC fatiguing contraction (within each experimental session)	923 ± 459	1099 ± 155	not reported	Interaction $p = 0.090$ ($\eta^2 = 0.33$); no significant post hoc effects	Partial η^2 where given	Yes only at $\alpha = 0.10$ (interaction); No at $\alpha = 0.05$	Unclear (contractile property)	Randomised counterbalanced crossover (≥2-wk washout); no rehydration before testing. 2-way RM GLM (condition × time) with Fisher's LSD post hoc; significance set at $p < 0.05$ for main effects but $p < 0.10$ for INTERACTION effects (symbol #). Evoked contractile property. RFD: group × time interaction ($p < 0.10$)'.
Zubac et al., 2020 [43]	Control condition (n = 9)	20 Hz tetanic train – RFD (N m/s)	Pre vs post a sustained 70% MVC fatiguing contraction (within each experimental session)	716 ± 261	888 ± 207	not reported	Comparator (see RWL row)	see RWL row	n/a (comparator)	n/a (comparator)	—
Zubac et al., 2020 [43]	RWL condition (~3% BM in 3 d by food/fluid restriction; n = 9)	80 Hz tetanic train – force (Nm)	Pre vs post a sustained 70% MVC fatiguing contraction (within each experimental session)	212.5 ± 29.2	199.3 ± 15.9	not reported	No significant interaction effects for any 80 Hz variable	Partial η^2 where given	No	Null	Randomised counterbalanced crossover (≥2-wk washout); no rehydration before testing. 2-way RM GLM (condition × time) with Fisher's LSD post hoc; significance set at $p < 0.05$ for main effects but $p < 0.10$ for INTERACTION effects (symbol #). Evoked contractile property.
Zubac et al., 2020 [43]	Control condition (n = 9)	80 Hz tetanic train – force (Nm)	Pre vs post a sustained 70% MVC fatiguing contraction (within each experimental session)	218.7 ± 35.8	215.6 ± 64.1	not reported	Comparator (see RWL row)	see RWL row	n/a (comparator)	n/a (comparator)	—

Study (first author, year) [ref]	Group / condition	Outcome	Time point	Pre value (mean ± SD)	Post value (mean ± SD)	Change (absolute and/or %)	Test statistic and p value	Effect size (95% CI if reported)	Statistically significant (Yes / No)	Direction (Favourable / Unfavourable / Null)	Notes
Zubac et al., 2020 [43]	RWL condition (~3% BM in 3 d by food/fluid restriction; n = 9)	80 Hz tetanic train – CT (ms)	Pre vs post a sustained 70% MVC fatiguing contraction (within each experimental session)	758.8 ± 262.7	730.1 ± 292.9	not reported	No significant interaction	Partial η² where given	No	Null	Randomised counterbalanced crossover (≥2-wk washout); no rehydration before testing. 2-way RM GLM (condition × time) with Fisher's LSD post hoc; significance set at p < 0.05 for main effects but p < 0.10 for INTERACTION effects (symbol #). Evoked contractile property.
Zubac et al., 2020 [43]	Control condition (n = 9)	80 Hz tetanic train – CT (ms)	Pre vs post a sustained 70% MVC fatiguing contraction (within each experimental session)	769.2 ± 254.6	735.1 ± 278.6	not reported	Comparator (see RWL row)	see RWL row	n/a (comparator)	n/a (comparator)	—
Zubac et al., 2020 [43]	RWL condition (~3% BM in 3 d by food/fluid restriction; n = 9)	80 Hz tetanic train – HRT (ms)	Pre vs post a sustained 70% MVC fatiguing contraction (within each experimental session)	250.4 ± 113.5	244.8 ± 136.7	not reported	No significant interaction	Partial η² where given	No	Null	Randomised counterbalanced crossover (≥2-wk washout); no rehydration before testing. 2-way RM GLM (condition × time) with Fisher's LSD post hoc; significance set at p < 0.05 for main effects but p < 0.10 for INTERACTION effects (symbol #). Evoked contractile property.
Zubac et al., 2020 [43]	Control condition (n = 9)	80 Hz tetanic train – HRT (ms)	Pre vs post a sustained 70% MVC fatiguing contraction (within each experimental session)	272.6 ± 162.7	229.9 ± 155.8	not reported	Comparator (see RWL row)	see RWL row	n/a (comparator)	n/a (comparator)	—

Study (first author, year) [ref]	Group / condition	Outcome	Time point	Pre value (mean ± SD)	Post value (mean ± SD)	Change (absolute and/or %)	Test statistic and p value	Effect size (95% CI if reported)	Statistically significant (Yes / No)	Direction (Favourable / Unfavourable / Null)	Notes
Zubac et al., 2020 [43]	RWL condition (~3% BM in 3 d by food/fluid restriction; n = 9)	80 Hz tetanic train – TTE (ms)	Pre vs post a sustained 70% MVC fatiguing contraction (within each experimental session)	497.6 ± 224.0	489.6 ± 273.3	not reported	No significant interaction	Partial η² where given	No	Null	Randomised counterbalanced crossover (≥2-wk washout); no rehydration before testing. 2-way RM GLM (condition × time) with Fisher's LSD post hoc; significance set at $p < 0.05$ for main effects but $p < 0.10$ for INTERACTION effects (symbol #). Evoked contractile property.
Zubac et al., 2020 [43]	Control condition (n = 9)	80 Hz tetanic train – TTE (ms)	Pre vs post a sustained 70% MVC fatiguing contraction (within each experimental session)	550.2 ± 325.6	459.8 ± 311.7	not reported	Comparator (see RWL row)	see RWL row	n/a (comparator)	n/a (comparator)	—
Zubac et al., 2020 [43]	RWL condition (~3% BM in 3 d by food/fluid restriction; n = 9)	80 Hz tetanic train – RFD (N m/s)	Pre vs post a sustained 70% MVC fatiguing contraction (within each experimental session)	1405 ± 241	1417 ± 277	not reported	No significant interaction	Partial η² where given	No	Null	Randomised counterbalanced crossover (≥2-wk washout); no rehydration before testing. 2-way RM GLM (condition × time) with Fisher's LSD post hoc; significance set at $p < 0.05$ for main effects but $p < 0.10$ for INTERACTION effects (symbol #). Evoked contractile property.
Zubac et al., 2020 [43]	Control condition (n = 9)	80 Hz tetanic train – RFD (N m/s)	Pre vs post a sustained 70% MVC fatiguing contraction (within each experimental session)	1491 ± 261	1353 ± 441	not reported	Comparator (see RWL row)	see RWL row	n/a (comparator)	n/a (comparator)	—

Study (first author, year) [ref]	Group / condition	Outcome	Time point	Pre value (mean ± SD)	Post value (mean ± SD)	Change (absolute and/or %)	Test statistic and p value	Effect size (95% CI if reported)	Statistically significant (Yes / No)	Direction (Favourable / Unfavourable / Null)	Notes
Zubac et al., 2020 [43]	RWL condition (~3% BM in 3 d by food/fluid restriction; n = 9)	20/80 Hz ratio (low-frequency fatigue index)	Pre vs post a sustained 70% MVC fatiguing contraction (within each experimental session)	74 ± 8	75 ± 9	not reported	Time p = 0.931; interaction p = 0.827	Partial η^2 where given	No	Null	Randomised counterbalanced crossover (≥2-wk washout); no rehydration before testing. 2-way RM GLM (condition × time) with Fisher's LSD post hoc; significance set at p < 0.05 for main effects but p < 0.10 for INTERACTION effects (symbol #). Evoked contractile property.
Zubac et al., 2020 [43]	Control condition (n = 9)	20/80 Hz ratio (low-frequency fatigue index)	Pre vs post a sustained 70% MVC fatiguing contraction (within each experimental session)	77 ± 15	76 ± 12	not reported	Comparator (see RWL row)	see RWL row	n/a (comparator)	n/a (comparator)	—
Zubac et al., 2020 [43]	RWL condition (~3% BM in 3 d by food/fluid restriction; n = 9)	Post-fatigue doublet – peak twitch torque (Nm)	After the 70% MVC fatigue protocol	not reported	55.6 ± 12.4	not reported	RWL vs control p = 0.551	not reported	No	Null	Randomised counterbalanced crossover (≥2-wk washout); no rehydration before testing. 2-way RM GLM (condition × time) with Fisher's LSD post hoc; significance set at p < 0.05 for main effects but p < 0.10 for INTERACTION effects (symbol #).
Zubac et al., 2020 [43]	Control condition (n = 9)	Post-fatigue doublet – peak twitch torque (Nm)	After the 70% MVC fatigue protocol	not reported	54.2 ± 11.8	not reported	Reference condition (see RWL row)	see RWL row	n/a (reference)	n/a (reference)	—

Study (first author, year) [ref]	Group / condition	Outcome	Time point	Pre value (mean ± SD)	Post value (mean ± SD)	Change (absolute and/or %)	Test statistic and p value	Effect size (95% CI if reported)	Statistically significant (Yes / No)	Direction (Favourable / Unfavourable / Null)	Notes
Zubac et al., 2020 [43]	RWL condition (~3% BM in 3 d by food/fluid restriction; n = 9)	Post-fatigue doublet – CT (ms)	After the 70% MVC fatigue protocol	not reported	85.6 ± 24.4	not reported	RWL vs control p = 0.479	not reported	No	Null	Randomised counterbalanced crossover (≥2-wk washout); no rehydration before testing. 2-way RM GLM (condition × time) with Fisher's LSD post hoc; significance set at $p < 0.05$ for main effects but $p < 0.10$ for INTERACTION effects (symbol #).
Zubac et al., 2020 [43]	Control condition (n = 9)	Post-fatigue doublet – CT (ms)	After the 70% MVC fatigue protocol	not reported	76.6 ± 17.2	not reported	Reference condition (see RWL row)	see RWL row	n/a (reference)	n/a (reference)	—
Zubac et al., 2020 [43]	RWL condition (~3% BM in 3 d by food/fluid restriction; n = 9)	Post-fatigue doublet – HRT (ms)	After the 70% MVC fatigue protocol	not reported	137.1 ± 57.1	not reported	RWL vs control p = 0.279	not reported	No	Null	Randomised counterbalanced crossover (≥2-wk washout); no rehydration before testing. 2-way RM GLM (condition × time) with Fisher's LSD post hoc; significance set at $p < 0.05$ for main effects but $p < 0.10$ for INTERACTION effects (symbol #).
Zubac et al., 2020 [43]	Control condition (n = 9)	Post-fatigue doublet – HRT (ms)	After the 70% MVC fatigue protocol	not reported	188.2 ± 95.0	not reported	Reference condition (see RWL row)	see RWL row	n/a (reference)	n/a (reference)	—

Study (first author, year) [ref]	Group / condition	Outcome	Time point	Pre value (mean ± SD)	Post value (mean ± SD)	Change (absolute and/or %)	Test statistic and p value	Effect size (95% CI if reported)	Statistically significant (Yes / No)	Direction (Favourable / Unfavourable / Null)	Notes
Zubac et al., 2020 [43]	RWL condition (~3% BM in 3 d by food/fluid restriction; n = 9)	Post-fatigue doublet – TTE (ms)	After the 70% MVC fatigue protocol	not reported	250.0 ± 96.8	not reported	RWL vs control p = 0.140	not reported	No	Null	Randomised counterbalanced crossover (≥2-wk washout); no rehydration before testing. 2-way RM GLM (condition × time) with Fisher's LSD post hoc; significance set at p < 0.05 for main effects but p < 0.10 for INTERACTION effects (symbol #).
Zubac et al., 2020 [43]	Control condition (n = 9)	Post-fatigue doublet – TTE (ms)	After the 70% MVC fatigue protocol	not reported	376.3 ± 190.1	not reported	Reference condition (see RWL row)	see RWL row	n/a (reference)	n/a (reference)	—
Ceylan et al., 2022 [44]	RWL group (5% BM in 48 h, self-selected methods; weight-cyclers; n = 9)	SJFT total throw number, 1st of 3 consecutive SJFTs (10-min rest)	M1 baseline (48 h before simulated weigh-in) vs M2 (after RWL, at simulated weigh-in); M3 after 15 h recovery (RWG) in notes	27 ± 1 (95% CI 25–29)	27 ± 2 (23–29)	not reported	Total throws: time F(1.9,30.4) = 7.70, p < 0.001, η² = 0.33; 'RWL and measurement times did not affect total throws during each SJFT' (F(1.8,29.2) = 1.66, p = 0.21; F(1,16) = 1.15, p = 0.30); changes similar among measurements F(3.1,49.5) = 2.12, p = 0.11; one-way RM ANOVA within RWL: fewer throws after RWL (M2) than after 15 h recovery (M3), p < 0.05 (exact p NR; not specified per SJFT)	η² reported for ANOVA effects only	No vs baseline; Yes (post hoc M2 vs M3 only)	Unfavourable (vs recovery only)	Non-randomised allocation (weight-cyclers vs non-cyclers). Split-plot ANOVA group × measurement day × test (2 × 3 × 3). Body mass change was not statistically significant (time F(1.2,19.6) = 0.93, p = 0.37; interaction F = 1.06); RWL 71.2 ± 9.3 → 67.7 ± 9.0 kg. M3 value: 27 ± 2 (25–31). Table 4 labels the three consecutive tests 'SJFT a, b, c' and the footnote calls them 'parts of the SJFT', which conflicts with the values (~27 throws = whole-test totals). The significant post hoc difference is M2 vs M3 (recovery), not M2 vs baseline.

Study (first author, year) [ref]	Group / condition	Outcome	Time point	Pre value (mean ± SD)	Post value (mean ± SD)	Change (absolute and/or %)	Test statistic and p value	Effect size (95% CI if reported)	Statistically significant (Yes / No)	Direction (Favourable / Unfavourable / Null)	Notes
Ceylan et al., 2022 [44]	Control group (no weight loss; n = 9)	SJFT total throw number, 1st of 3 consecutive SJFTs	M1 baseline (48 h before simulated weigh-in) vs M2 (after RWL, at simulated weigh-in); M3 after 15 h recovery (RWG) in notes	27 ± 1 (95% CI 25–30)	28 ± 1 (26–30)	not reported	Same ANOVA; no significant within-group change reported for control	not reported	No	Null	M3 value: 28 ± 2 (26–32).
Ceylan et al., 2022 [44]	RWL group (5% BM in 48 h, self-selected methods; weight-cyclers; n = 9)	SJFT total throw number, 2nd of 3 consecutive SJFTs (10-min rest)	M1 baseline (48 h before simulated weigh-in) vs M2 (after RWL, at simulated weigh-in); M3 after 15 h recovery (RWG) in notes	27 ± 1 (25–27)	26 ± 2 (23–28)	not reported	Total throws: time $F(1.9,30.4) = 7.70$, $p < 0.001$, $\eta^2 = 0.33$; 'RWL and measurement times did not affect total throws during each SJFT' ($F(1.8,29.2) = 1.66$, $p = 0.21$; $F(1,16) = 1.15$, $p = 0.30$); changes similar among measurements $F(3.1,49.5) = 2.12$, $p = 0.11$; one-way RM ANOVA within RWL: fewer throws after RWL (M2) than after 15 h recovery (M3), $p < 0.05$ (exact p NR; not specified per SJFT)	η^2 reported for ANOVA effects only	No vs baseline; Yes (post hoc M2 vs M3 only)	Unfavourable (vs recovery only)	Non-randomised allocation (weight-cyclers vs non-cyclers). Split-plot ANOVA group × measurement day × test (2 × 3 × 3). Body mass change was not statistically significant (time $F(1.2,19.6) = 0.93$, $p = 0.37$; interaction $F = 1.06$); RWL 71.2 ± 9.3 → 67.7 ± 9.0 kg. M3 value: 28 ± 2 (26–31). Table 4 labels the three consecutive tests 'SJFT a, b, c' and the footnote calls them 'parts of the SJFT', which conflicts with the values (~27 throws = whole-test totals). The significant post hoc difference is M2 vs M3 (recovery), not M2 vs baseline.

Study (first author, year) [ref]	Group / condition	Outcome	Time point	Pre value (mean ± SD)	Post value (mean ± SD)	Change (absolute and/or %)	Test statistic and p value	Effect size (95% CI if reported)	Statistically significant (Yes / No)	Direction (Favourable / Unfavourable / Null)	Notes
Ceylan et al., 2022 [44]	Control group (no weight loss; n = 9)	SJFT total throw number, 2nd of 3 consecutive SJFTs	M1 baseline (48 h before simulated weigh-in) vs M2 (after RWL, at simulated weigh-in); M3 after 15 h recovery (RWG) in notes	28 ± 1 (26–30)	27 ± 2 (26–32)	not reported	Same ANOVA; no significant within-group change reported for control	not reported	No	Null	M3 value: 28 ± 2 (26–32).
Ceylan et al., 2022 [44]	RWL group (5% BM in 48 h, self-selected methods; weight-cyclers; n = 9)	SJFT total throw number, 3rd of 3 consecutive SJFTs (10-min rest)	M1 baseline (48 h before simulated weigh-in) vs M2 (after RWL, at simulated weigh-in); M3 after 15 h recovery (RWG) in notes	27 ± 2 (25–29)	26 ± 2 (24–29)	not reported	Total throws: time $F(1.9,30.4) = 7.70$, $p < 0.001$, $\eta^2 = 0.33$; 'RWL and measurement times did not affect total throws during each SJFT' ($F(1.8,29.2) = 1.66$, $p = 0.21$; $F(1,16) = 1.15$, $p = 0.30$); changes similar among measurements $F(3.1,49.5) = 2.12$, $p = 0.11$; one-way RM ANOVA within RWL: fewer throws after RWL (M2) than after 15 h recovery (M3), $p < 0.05$ (exact p NR; not specified per SJFT)	η^2 reported for ANOVA effects only	No vs baseline; Yes (post hoc M2 vs M3 only)	Unfavourable (vs recovery only)	Non-randomised allocation (weight-cyclers vs non-cyclers). Split-plot ANOVA group × measurement day × test (2 × 3 × 3). Body mass change was not statistically significant (time $F(1.2,19.6) = 0.93$, $p = 0.37$; interaction $F = 1.06$); RWL 71.2 ± 9.3 → 67.7 ± 9.0 kg. M3 value: 29 ± 2 (26–33). Table 4 labels the three consecutive tests 'SJFT a, b, c' and the footnote calls them 'parts of the SJFT', which conflicts with the values (~27 throws = whole-test totals). The significant post hoc difference is M2 vs M3 (recovery), not M2 vs baseline.

Study (first author, year) [ref]	Group / condition	Outcome	Time point	Pre value (mean ± SD)	Post value (mean ± SD)	Change (absolute and/or %)	Test statistic and p value	Effect size (95% CI if reported)	Statistically significant (Yes / No)	Direction (Favourable / Unfavourable / Null)	Notes
Ceylan et al., 2022 [44]	Control group (no weight loss; n = 9)	SJFT total throw number, 3rd of 3 consecutive SJFTs	M1 baseline (48 h before simulated weigh-in) vs M2 (after RWL, at simulated weigh-in); M3 after 15 h recovery (RWG) in notes	28 ± 2 (24–30)	28 ± 2 (26–31)	not reported	Same ANOVA; no significant within-group change reported for control	not reported	No	Null	M3 value: 28 ± 2 (27–32).
Ceylan et al., 2022 [44]	RWL group (5% BM in 48 h, self-selected methods; weight-cyclers; n = 9)	SJFT index (bpm·throws^{-1}), 3 consecutive SJFTs	M1 baseline (48 h before simulated weigh-in) vs M2 (after RWL, at simulated weigh-in); M3 after 15 h recovery (RWG) in notes	Baseline (single SJFT, Table 1): 12.7 ± 0.9 (95% CI 12.0–13.4)	After RWL: SJFT1 12.47 ± 1.13; SJFT2 12.84 ± 1.18; SJFT3 12.90 ± 1.07	not reported	Group × time × test $F(2.9,46.3) = 1.24$, $p = 0.31$; group $F(1,16) = 6.43$, $p = 0.02$ ($\eta^2 = 0.29$); time $F(1.7,26.4) = 8.05$, $p = 0.01$ ($\eta^2 = 0.33$); test $F(1.3,21.4) = 2.01$, $p = 0.17$. One-way RM ANOVA within RWL $F(2.3,24.6) = 4.87$, $p = 0.02$ ($\eta^2 = 0.38$). Post hoc after RWL vs after recovery: p = 0.00–0.05 (Table 5 matrix)	$\eta^2 = 0.38$ (within-RWL effect)	No (3-way interaction); Yes (within-RWL, M2 vs M3)	Unfavourable (vs recovery only)	Non-randomised allocation (weight-cyclers vs non-cyclers). Split-plot ANOVA group × measurement day × test (2 × 3 × 3). Body mass change was not statistically significant (time $F(1.2,19.6) = 0.93$, $p = 0.37$; interaction F = 1.06); RWL 71.2 ± 9.3 → 67.7 ± 9.0 kg. After-recovery (M3) index: SJFT1 11.86 ± 0.89; SJFT2 11.61 ± 1.01; SJFT3 11.45 ± 1.00. Higher index = worse. Groups already differed at baseline (control 11.4 ± 0.7 vs RWL 12.7 ± 0.9, p = 0.01). Comparison of M2 with baseline (M1) not reported. Table 5 p values include 'p = 0.05' treated as significant by the authors.

Study (first author, year) [ref]	Group / condition	Outcome	Time point	Pre value (mean ± SD)	Post value (mean ± SD)	Change (absolute and/or %)	Test statistic and p value	Effect size (95% CI if reported)	Statistically significant (Yes / No)	Direction (Favourable / Unfavourable / Null)	Notes
Ceylan et al., 2022 [44]	Control group (no weight loss; n = 9)	SJFT index (bpm·throws⁻¹), 3 consecutive SJFTs	M1 baseline (48 h before simulated weigh-in) vs M2 (after RWL, at simulated weigh-in); M3 after 15 h recovery (RWG) in notes	Baseline (Table 1): 11.4 ± 0.7 (95% CI 10.9–12.0)	not reported	not reported	Same ANOVA; control values at M2/M3 not reported	not reported	not reported	not reported	Control index after baseline not tabulated.
Ceylan et al., 2022 [44]	RWL group (5% BM in 48 h, self-selected methods; weight-cyclers; n = 9)	Heart rate 1 min after SJFT (bpm)	M2 (after RWL)	not reported	After 2nd SJFT: 150; after 3rd SJFT: 151 (SD NR)	not reported	Group main effect on HR $F(1,16) = 10.50$, $p = 0.01$ ($\eta^2 = 0.40$); group × time $F(1.98,31.67) = 1.42$, $p = 0.26$; group × time × test $F(5.15,82.43) = 0.97$, $p = 0.44$. Between-group post hoc at M2: after 2nd SJFT $p = 0.04$; after 3rd SJFT $p = 0.03$	$\eta^2 = 0.40$ (group effect)	No (interaction); Yes (between-group post hoc at M2)	Unfavourable	Non-randomised allocation (weight-cyclers vs non-cyclers). Split-plot ANOVA group × measurement day × test (2 × 3 × 3). Body mass change was not statistically significant (time $F(1.2,19.6) = 0.93$, $p = 0.37$; interaction $F = 1.06$); RWL 71.2 ± 9.3 → 67.7 ± 9.0 kg. Remaining HR data only in Figure 3. Groups already differed in HR at baseline (Table 1: SJFT B, C and 1-min HR higher in RWL, $p \leq 0.04$).
Ceylan et al., 2022 [44]	Control group (no weight loss; n = 9)	Heart rate 1 min after SJFT (bpm)	M2	not reported	After 2nd SJFT: 143; after 3rd SJFT: 137 (SD NR)	not reported	Comparator (see RWL row)	see RWL row	n/a (comparator)	n/a (comparator)	—
Ceylan et al., 2022 [44]	RWL group (5% BM in 48 h, self-selected methods; weight-cyclers; n = 9)	Blood lactate after SJFTs (mmol/L)	M1, M2, M3 (1st and 6th min after each SJFT; 1st, 6th, 15th min after the last)	not reported	Figure/text only	not reported	No time × group interaction $F(1.54,24.63) = 0.64$, $p = 0.50$; no group effect $F(1,16) = 0.04$, $p = 0.90$; time $F(1.54,24.63) = 1.55$, $p = 0.23$	$\eta^2 = 0.04$ (interaction)	No	Null	Descriptive lactate values not tabulated.

Study (first author, year) [ref]	Group / condition	Outcome	Time point	Pre value (mean ± SD)	Post value (mean ± SD)	Change (absolute and/or %)	Test statistic and p value	Effect size (95% CI if reported)	Statistically significant (Yes / No)	Direction (Favourable / Unfavourable / Null)	Notes
Rossi et al., 2022 [45]	BL → P1 (RWL phase: −5% BM in 3 d + HISST; n = 10)	Handgrip strength, right hand (HG right) (kg)	BL (baseline) vs P1 (after 5% BM loss in 3 d + HISST on day 3); P2 (7 d later, HISST without RWL) in notes	55.55 ± 5.81	56.66 ± 7.28	not reported	One-way RM ANOVA across BL, P1, P2: F = 0.104, p = 0.857; no significant pairwise difference	Partial η^2 = 0.013	No	Null	Single group of 10 male national-level Greco-Roman wrestlers measured at three time points (described by the authors as 'cross-over', but phases were not randomised or counterbalanced). One-way RM ANOVA with LSD post hoc; significance set at p ≤ 0.05. The PDF is Annales Kinesiologiae 12(2), 2021 (received 2022-04-01). P2 value: 56.44 ± 8.35.
Rossi et al., 2022 [45]	BL → P1 (RWL phase: −5% BM in 3 d + HISST; n = 10)	Handgrip strength, left hand (HG left) (kg)	BL (baseline) vs P1 (after 5% BM loss in 3 d + HISST on day 3); P2 (7 d later, HISST without RWL) in notes	54.55 ± 6.06	57.55 ± 4.44	not reported	One-way RM ANOVA across BL, P1, P2: F = 1.845, p = 0.199 (no main effect). LSD post hoc: P1 > P2, p = 0.039; P1 vs BL not significant (p NR)	Partial η^2 = 0.187	No (main effect) / Yes (post hoc P1 vs P2 only)	Favourable (P1 vs P2 only)	Single group of 10 male national-level Greco-Roman wrestlers measured at three time points (described by the authors as 'cross-over', but phases were not randomised or counterbalanced). One-way RM ANOVA with LSD post hoc; significance set at p ≤ 0.05. The PDF is Annales Kinesiologiae 12(2), 2021 (received 2022-04-01). P2 value: 54 ± 4. The only significant comparison is between the RWL phase (P1) and the subsequent no-RWL phase (P2), not versus baseline.

Abbreviations: AE, area of 95% ellipse; BL, baseline; BM, body mass; BRUMS, Brunel Mood Scale; CG, control group; CMJ, countermovement jump; CT, contraction time; DALDA, Daily Analysis of Life Demands in Athletes; DHY/DEHY, dehydration condition; EG, experimental group; FTP, functional threshold power; FWB/SWB, fresh/salt water bathing; GPAI, Game Performance Assessment Instrument; HG/HGS, handgrip strength; HISST, high-intensity sport-specific training; HR, heart rate; HRT, half-relaxation time; IGS, isometric grip strength; ILT, individual lactate threshold; IMTP, isometric mid-thigh pull; ITTS, isometric trunk traction strength; JGST, judogi grip strength test; 4LT, 4 mmol/L lactate threshold; MAPV/MMLV, mean anteroposterior/mediolateral velocity; MVC, maximal voluntary contraction; NR, not reported; NWL, no rapid weight loss; POMS, Profile of Mood States; PWL, progressive weight loss; RER, respiratory exchange ratio; RESTQ-Sport, Recovery-Stress Questionnaire for Athletes; RFD, rate of force development; RPE, rating of perceived exertion; RWG, rapid weight gain; RWL, rapid weight loss; SEI, skill execution index; SJ, squat jump; SJFT, Special Judo Fitness Test; TMD, total mood disturbance; TTE, time to end; TW-5/TW-30, tokui-waza repetitions in 5/30 s; VA, voluntary activation; WC, weight cyclers; WL, weight loss; WR, weight reduction; WS, weight stable
